# The synergistic role of moisture and the silver–lead(ii) oxide interface in room-temperature catalytic oxidation of gaseous formaldehyde

**DOI:** 10.1039/d6ra01138a

**Published:** 2026-08-13

**Authors:** Kumar Vikrant, Ki-Hyun Kim, Philippe M. Heynderickx, Changho Yeon, Chan-Woo Lee

**Affiliations:** a Center for Materials Innovation and Technology, VinUniversity Vinhomes Ocean Park, Gia Lâm Hanoi 100000 Vietnam; b College of Engineering and Computer Science, VinUniversity Vinhomes Ocean Park, Gia Lâm Hanoi 100000 Vietnam; c Department of Civil and Environmental Engineering, Hanyang University 222 Wangsimni-Ro Seoul 04763 Republic of Korea kkim61@hanyang.ac.kr kkim61@nate.com; d Center for Green Chemistry and Environmental Biotechnology (GREAT), Ghent University Global Campus 119-5 Songdo Munhwa-ro Yeonsu-gu Incheon 406-840 Republic of Korea; e Department of Green Chemistry and Technology, Faculty of Bioscience Engineering, Ghent University Coupure Links 653 B-9000 Ghent Belgium; f Department of Energy and Materials Engineering, Dongguk University 30 Pildong-ro 1-gil Jung-gu Seoul 04620 Republic of Korea

## Abstract

Formaldehyde (FA), a carcinogenic oxygenated volatile organic compound, represents a principal challenge for indoor air quality management efforts. This study investigates the oxidation potential of lead(ii) oxide (PbO)-supported silver (Ag) catalysts against FA under dark and low-temperature conditions, including room temperature (RT) conditions. FA (100 ppm) conversion (*X*_FA_) on Ag/PbO increases with increasing Ag content while being lowered with reduction pre-treatment (R). The RT *X*_FA_ of these catalysts decreases (at gas hourly space velocity: 9436 h^−1^) as follows: 0.9%-Ag/PbO (83%) > 0.9%-Ag/PbO-R (56%) > 0.2%-Ag/PbO (39%) > 0.02%-Ag/PbO (35%). Interestingly, the 0.9%-Ag/PbO catalyst achieves 100% *X*_FA_ at RT above 60% relative humidity, demonstrating exceptional FA removal efficiency. While complete mineralization to carbon dioxide requires a mild temperature increase to 80 °C, the catalyst effectively sequesters FA as surface-bound intermediates (*e.g.*, dioxymethylene and carbonates) at RT, functioning as a transformative adsorptive sink. Density functional theory calculations and characterization results reveal that the Ag/PbO interface effectively activates molecular oxygen (O_2_) and water (H_2_O) to generate reactive oxygen species (*O) and hydroxyl (OH) groups for the oxidation of FA molecules. Compared to Ag/PbO-R, the Ag/PbO surface exhibits a higher concentration of *O under oxidative operational conditions, as oxygen species preferentially accumulate at interfacial sites rather than being consumed to restore the lattice structure. Additionally, moisture plays a crucial role by facilitating the formation of *OH as well as the key *CH_2_(OH)_2_ intermediate. This study mechanistically dissects the charge-transfer dynamics at the Ag–PbO interface and the cooperative O_2_/H_2_O chemistry that arises from this junction.

## Abstract 2

Moisture-promotes oxidation of formaldehyde (FA) on a PbO-supported Ag catalyst.

## Graphical abstract

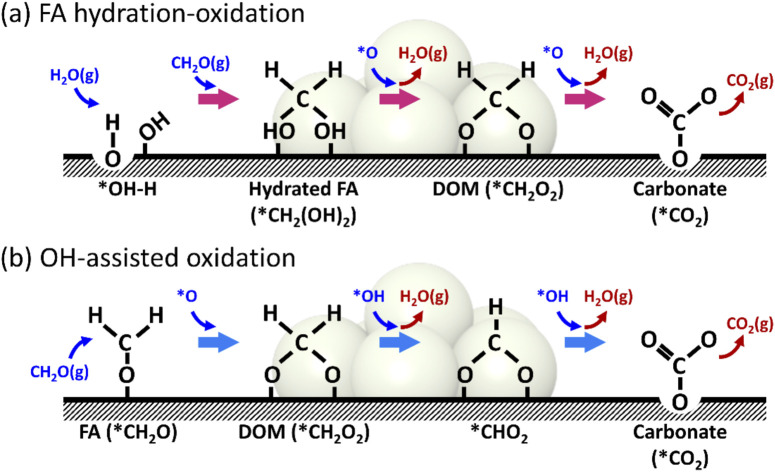

## 1. Introduction

Formaldehyde (FA) is a typical volatile organic compound often encountered in indoor air. It has harmful effects on multiple physiological systems, including the neurological and pulmonary systems, and has also been linked to cancer.^1,2^ A permissible FA exposure limit of 750 ppb (8 h time-weighted average) has been set by the United States Occupational Safety and Health Administration.^3^ In this regard, developing effective FA removal technologies is crucial for indoor air quality management. Among various technical options, catalytic FA oxidation into relatively harmless compounds (*e.g.*, water (H_2_O) and carbon dioxide (CO_2_)) under dark and low-temperature (DLT) situations has been recognized as an effective strategy due to the favorable cost economics and process stability.^2,4^

The supported noble metal (*e.g.*, palladium (Pd), platinum (Pt), gold (Au), rhodium (Rh), silver (Ag), and iridium (Ir)) catalysts (SNMCs) and transition metal oxides (*e.g.*, manganese dioxide (MnO_2_), cerium(iv) oxide (CeO_2_), and cobalt(ii,iii) oxide (Co_3_O_4_)) have frequently been utilized for the oxidative removal of FA from indoor air.^2,4–7^ Despite the economic merits, transition metal oxides have a much lower ability to activate molecular oxygen (O_2_) and H_2_O under DLT conditions, leading to poorer FA oxidation activity than SNMCs.^7,8^ Ag is much cheaper than other noble metals, while highly active for the FA oxidation reaction at low temperatures. Consequently, the utility of supported Ag catalysts has been studied intensively for the oxidative removal of FA from indoor air.^5,9–11^

Support and noble metal species can play multiple synergistic roles with high catalytic activities to cover O_2_ (and H_2_O) activation and FA adsorption.^12^ Noble metal site activity is usually governed by its dispersion and particle size, which can be attained by adding alkali metals or by complicated synthesis routes.^13,14^ The strong metal–support interaction could weaken the noble metal–noble metal bond and result in the formation of the noble metal–support or noble metal–oxygen–support bond.^15,16^ Such a process would lower the particle size and increase the dispersion of the noble metal species. Furthermore, supports with active surface sites can promote FA adsorption and activation to boost overall catalytic activity. Noticeably, supports rich in surface hydroxyls (OH) have been utilized for oxidative removal of FA due to favorable FA adsorption *via* hydrogen bonding.^17,18^ Dehydrogenation of formate (HCOO*), a typical intermediate formed during the oxidation of FA molecules, into CO_2_ can also be catalyzed by surface OH.^17^

Although OH-rich supports like titanium dioxide (TiO_2_) and aluminum oxide (Al_2_O_3_) are common, lead(ii) oxide (PbO) has been reported to be a uniquely effective support.^12^ It was found that PbO adsorbs FA more favorably than Al_2_O_3_*via* a distinct chemisorption pathway where hydrated FA (methylene glycol (CH_2_(OH)_2_)) forms a ring-like PbCH_2_O_2_ species.^12^ This unique reactivity enabled a Pt/PbO catalyst to achieve 100% FA conversion (*X*_FA_) at 25 °C, dramatically outperforming a conventional Pt/Al_2_O_3_ catalyst, which only reached 45% conversion under identical conditions.^12^ This precedent establishes PbO as a scientifically compelling, albeit toxic, model support for fundamental catalytic studies. Acknowledging the toxicity of PbO, the authors do not intend to propose this material for direct practical application; rather, the objective is to conduct a fundamental investigation into the Ag–PbO interface. This approach, which seeks foundational insights from unconventional materials, is consistent with prior work on systems like mercury (Hg)/TiO_2_ for hydrogen sulfide (H_2_S) removal.^19^ Indeed, this focus on fundamental mechanisms over immediate applicability is a vital approach in catalysis, as evidenced by recent studies utilizing supports based on depleted uranium oxides to explore novel pathways for CO_2_ methanation.^20,21^ Building on the findings for Pt/PbO, this study aims to further elucidate the FA–PbO interaction and the critical role of moisture by exploring a more abundant and cost-effective noble metal, Ag.

Ag valence state plays a crucial role in the oxidative removal of FA from indoor air.^5,9^ Nevertheless, information on Ag valence state-mediated promotion of catalytic activity in FA oxidation remains scarce.^9^ Metallic Ag (Ag^0^) has often been reported to be more favorable for O_2_ activation to enhance the FA oxidation activity.^22–26^ In contrast, silver oxide (Ag_2_O (Ag^+^)) has also been reported to dominate the activity of catalysts.^27,28^ A recent study also proposed the co-function mechanism to help understand the role of the Ag valence state in the FA oxidation reaction.^5^ Ag^+^ species primarily contribute to FA oxidation at lower temperatures (<140 °C), while Ag^0^ becomes the prominent contributor at temperatures above 140 °C per the co-function mechanism.^5^ Nevertheless, Ag^0^ and Ag^+^ species achieved complete FA oxidation below 80 °C.^29^ Also, partially oxidized Ag (Ag^*δ*+^ (0 < *δ* < 1)) species were reported to be more active than Ag^0^.^30–32^ On the other hand, the following FA oxidation activity order was suggested for the supported Ag catalysts:^9^ Ag^+^ + Ag^0^ + Ag^*δ*+^ > Ag^*δ*+^ > Ag^0^ > Ag^+^. Due to these contrasting results, it is imperative to learn more about the role of supported Ag catalysts in the oxidative removal of FA from indoor air.

In this study, the potential of Ag/PbO in oxidative removal of FA from indoor air under DLT conditions was explored in association with the impact of various process variables on catalytic activity (*e.g.*, Ag loading and composition, flow rate, catalyst mass (*m*_cat_), FA concentration, time-on-stream (TOS), and relative humidity (RH)). Temperature-programmed reduction (TPR), density functional theory (DFT) calculations, and *in situ* diffuse reflectance infrared Fourier transform spectroscopy (DRIFTS) were used to provide theoretical and experimental insights into the FA oxidation mechanism and pathway. We believe this study to be the first report on the application of Ag/PbO for FA oxidation under DLT situations.

## 2. Experimental

### 2.1. Chemicals

Lead(ii) acetate trihydrate (Pb(CH_3_CO_2_)_2_·3H_2_O; 99.999% trace metal basis), paraformaldehyde (pFA (HO(CH_2_O_*n*_H)); 95%), quartz sand (SiO_2_), sodium hydroxide (NaOH) pellets (≥98%), and silver nitrate (AgNO_3_; ≥99%) were procured from Sigma-Aldrich (St. Louis, MO, USA). Nitrogen (N_2_; 99.999%), hydrogen (H_2_; 99.999%), and air (99.999%) cylinders were purchased from Union Gas Co., Ltd (Yongin, Republic of Korea).

### 2.2. Synthesis of the catalysts

PbO was synthesized using a precipitation approach, as reported earlier.^33^ First, 17 g of Pb(CH_3_CO_2_)_2_·3H_2_O was dissolved in 50 mL of deionized (DI) H_2_O in a glass beaker and stirred at 500 rpm for 15 min while maintaining a temperature of 90 °C. Next, 6 g of NaOH was introduced into the mixture, followed by continuous stirring at the same speed for an additional 15 min. The precipitates formed were collected using centrifugation and washed three times using DI H_2_O. The obtained solid was then dried in a convection oven for 15 h at 85 °C to obtain PbO. Ag was loaded onto the synthesized PbO following the conventional wet impregnation approach. A precise amount of AgNO_3_, corresponding to ∼1%, 0.2%, and 0.02% Ag catalysts, was dissolved in 20 mL of DI H_2_O in a glass beaker and stirred at 500 rpm for 15 min at room temperature (RT). Afterward, PbO was added to the Ag solution, and the mixture was stirred at 500 rpm for 180 min at RT. The suspension was then transferred to a convection oven, set to 85 °C, for 15 h to evaporate excess H_2_O, yielding dry catalyst powder. The actual Ag content in the synthesized catalysts (∼1%, 0.2%, and 0.02% Ag) was determined by inductively coupled plasma optical emission spectrometry (ICP-OES) and found to be 0.9%, 0.2%, and 0.02%, respectively (Table S1). Further instrumental characterization and TPR measurements of the PbO and Ag/PbO catalysts are provided in the SI.

### 2.3. Catalytic experiments

FA oxidation reactions were carried out at atmospheric pressure in a tubular quartz fixed-bed reactor with dimensions of 0.3 cm in diameter and 16 cm in length. A mixture of catalyst powder (50–150 mg) and 600 mg of SiO_2_ was placed inside the reactor and held in position by quartz wool plugs (Ohio Valley Specialty Company, Marietta, OH, USA) at both ends. The temperature of the catalyst bed was controlled *via* a heating block connected to an electric tube heater (TC200P [K], Misung Scientific Co., Ltd, Yangju, Republic of Korea). Before conducting the FA oxidation reactions, the catalyst bed underwent pre-treatment at 300 °C for 180 min, exposed to a flow of 50 mL min^−1^ of either N_2_ or a mixture of 10% H_2_ and N_2_. If the catalyst underwent reduction pre-treatment, an “-R” suffix was added to its name. For clarity in the subsequent characterization and discussion sections, the following nomenclature is used. Samples labeled without a suffix (*e.g.*, 0.9%-Ag/PbO) refer to the as-prepared catalysts, as synthesized and before any in-reactor pre-treatment. The suffix ‘-C’ (for ‘conditioned’) is used to designate a catalyst that has been characterized after undergoing the standard N_2_ pre-treatment at 300 °C. The suffix ‘-R’ (for ‘reduced’) designates a catalyst pre-treated under H_2_/N_2_. It should be noted that prior to all catalytic activity tests, the as-prepared catalysts were pre-treated in the reactor (with either N_2_ or H_2_/N_2_), as described previously. The gas flow rate was maintained with a vacuum pump (DOA-P704-AC, Gast Manufacturing Inc., Fair Plain, MI, USA), and the flow rate was controlled between 50 and 250 mL min^−1^ using a valve and flow calibrator (FC-M1, Sibata Scientific Technology Ltd, Saitama, Japan).

The gaseous primary standard (G-PS) of FA was generated using the thermal degradation technique, as described earlier.^34^ Briefly, pFA was heated to 130 °C in a quartz tube under a N_2_ flow (100 mL min^−1^) for 50 min, during which the FA-rich gas was captured in a 5 L polyester aluminum (PEA) bag (Top Trading Co., Ltd, Seoul, Republic of Korea). The FA concentration in the G-PS was measured using a high-performance liquid chromatography method utilizing 2,4-dinitrophenylhydrazine. To prepare the gaseous working standard (G-WS) of FA, the G-PS was diluted with air in a 100 L PEA bag to achieve concentrations in the 100–250 ppm range. The RH in the FA G-WS was minimal. For moisture-included experiments, a volume of 680–2000 µL of DI H_2_O (corresponding to 30–90% RH) was introduced into the 100 L G-WS bag using a 500 µL syringe (Trajan Scientific and Medical, Ringwood, Australia).

The temperature of the catalyst bed was gradually increased in 25 °C increments, starting from 30 °C (RT), until 100% *X*_FA_ was achieved, as described by eqn (1). The concentrations of FA at the reactor inlet and outlet were represented as [FA]_in_ and [FA]_out_, respectively. Each temperature was maintained for a minimum of 30 min to allow the system to reach a steady state. Both FA and CO_2_ were measured simultaneously using a gas chromatography (GC) system equipped with a methanizer and flame ionization detector (FID) (iGC-7200, DS Science Inc., Gwangju, Republic of Korea). The temperatures of the GC oven, methanizer, and FID were set at 150 °C, 320 °C, and 280 °C, respectively. For analysis, 500 µL gas samples were injected into the GC using a gas-tight syringe (Trajan Scientific and Medical, Ringwood, Australia). It is important to note that the methanizer-FID signal detected both carbon monoxide (CO) and CO_2_ together, as the system was not configured to separate these gases selectively. However, due to the excess O_2_ (21 vol%) used in the experiments, the methanizer-FID signal can be assumed to primarily reflect CO_2_. The absence of CO signals in the *in situ* DRIFTS spectra (see Section 3.5.1) further supports this assumption under the experimental conditions.

The CO_2_ yeld (*Y*_CO_2__) was calculated using eqn (2), with [CO_2_] denoting the concentration of CO_2_ in the reactor effluent. The steady-state reaction rate (*r* (µmol g^−1^ h^−1^)) was determined based on eqn (3), where *F*_FA_ represents the FA molar flow rate. The value for *r* derived from eqn (3) is applicable under differential conditions (*X*_FA_ < 20%), as the FA disappearance rate (−*r*_FA_) remains constant across the packed bed under these conditions. This is because the partial pressures of the reactant change little when *X*_FA_ is below 20%. Consequently, the *r* values obtained under integral conditions (*X*_FA_ > 20%) are intended solely for performance comparison rather than representing the actual reaction rate. In the FA adsorption experiments conducted at RT using a physically mixed PbO (120 mg)–SiO_2_ (600 mg) bed, the breakthrough (BT) level (%) and adsorption capacity (*q* (mg g^−1^)) were calculated using the methods described earlier.^35^ Prior to the adsorption experiment, the adsorbent bed was pre-conditioned at 300 °C for 180 min under an N_2_ flow (50 mL min^−1^).

*In situ* DRIFTS was performed to investigate the reaction pathway using a Spectrum™ 3 spectrometer (PerkinElmer, Inc., Waltham, MA, USA) coupled with an *in situ* diffuse-reflectance cell (DiffusIR™, PIKE Technologies, Madison, WI, USA).^36^ A mixture of 100 mg pFA and 200 mg SiO_2_ was packed into a quartz tube with quartz wool end plugs. N_2_ was passed through the tube at RT to produce gaseous FA. The FA produced in N_2_ was then diluted with air and directed into the diffuse-reflectance cell. If necessary, moisture was introduced into the FA stream. Before starting the DRIFTS analysis, the catalyst inside the diffuse-reflectance cell was flushed with N_2_.1
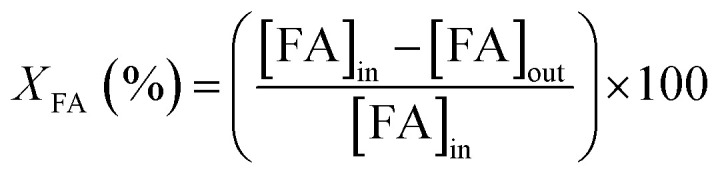
2
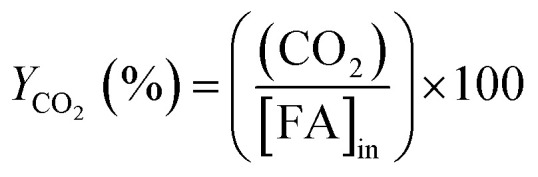
3
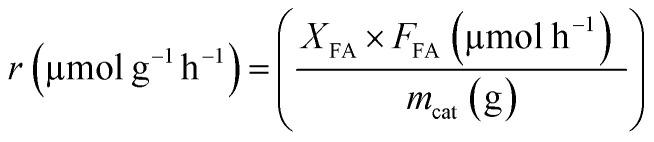


The external and internal mass transfer, external and internal heat transfer, and radial heat transfer gradient criteria were assessed and found to be nonsignificant at the reactor and catalyst particle scale.^37^ The relevant calculations are presented in the SI. The intrinsic kinetic data were collected by confirming the establishment of nonsignificant mass and heat transfer gradients within the reactor at the maximum *r*, *i.e.*, zero *X*_FA_ (catalyst bed entrance). Such an assessment was made by considering an FA, O_2_, and N_2_ mixture (see the SI).

### 2.4. DFT calculations

The theoretical spin-polarized simulations were performed to pinpoint the FA oxidation mechanism and surface phenomena on Ag/PbO and Ag/PbO-R using DFT with the Vienna *Ab initio* Simulation Package code by applying generalized gradient approximation for exchange–correlation functionals.^38–40^ The plane wave cutoff energy of 450 eV and *Γ*-only *k*-point of the Brillouin zone was set for all surface calculations.^41^ The convergence criteria of each electronic and ionic iteration were set to 10^−6^ eV and 0.02 eV Å^−1^, respectively. Electron distribution in the Ag cluster during the catalytic reaction was assessed using the Bader charge analysis.^42,43^ A slab model for PbO (100) was constructed according to the description provided in an earlier study on FA oxidation using Pt/PbO.^12^ The PbO (100) surface was specifically modeled using a 4-layer slab with a vacuum spacing of 17 Å inserted along the *z*-direction to prevent spurious periodic interactions. During structural relaxation, the bottom 2 layers were kept fixed at their bulk positions, while the upper layers and adsorbates were allowed to fully relax.

**Fig. 1:**
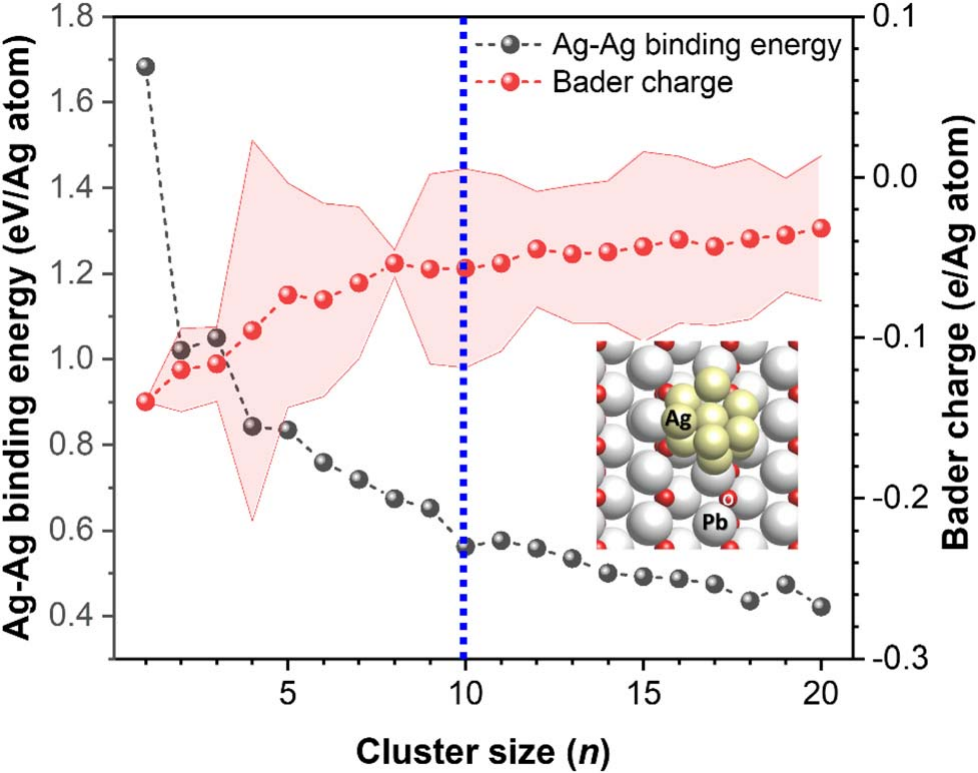
Determination of the optimal Ag nanocluster size on the PbO (100) surface using the GOFEE algorithm. The graph illustrates the Ag–Ag binding energy (black circles, left axis) and the average Bader charge per Ag atom (red circles, right axis) as a function of the cluster size (*n* = 1 to 20). The red shaded area represents the charge distribution variance within the cluster. The vertical blue dashed line at *n* = 10 indicates the selected representative cluster model (Ag_10_), where both the energetic stability and electronic properties are sufficiently converged. The inset shows the top view of the relaxed three-dimensional morphology for the optimal cluster model.

To determine the most representative nanoparticle (NP) catalyst model bridging computational feasibility and experimental relevance, a global structure search for Ag_*n*_ clusters (*n* = 1 to 20) on the PbO (100) slab was performed using the Gaussian process-based GOFEE (Global Optimization with First-principles Energy Evaluation) algorithm.^44^ Furthermore, Bader charge analysis was employed not only to track the charge transfer during the catalytic reaction but also to evaluate the convergence of the electronic charge state as a function of the cluster size.^42^ As illustrated in Fig. 1, both the Ag–Ag binding energy and the average Bader charge converge at *n* = 10, indicating that the Ag_10_ cluster provides a converged model for the local electronic environment. We acknowledge that this cluster is a necessary simplification of the larger 8.7 nm NPs observed experimentally and cannot fully represent their bulk electronic or strain properties. However, its size is sufficient to move beyond the extreme quantum confinement effects of smaller clusters. The model therefore offers a robust, computationally tractable method for probing the atom-scale reaction events at the metal–support perimeter, which are believed to govern the overall catalytic activity and are the primary focus of this mechanistic study. Notably, this computational determination strongly aligns with previous literature, which highlights that the Ag_10_ nanocluster exhibits exceptional structural stability and high symmetry when forming bonds on oxide surfaces.^45^ Therefore, based on our systematic search and its consistency with established studies, the Ag_10_/PbO (100) model was rigorously selected as the optimal minimum structural unit. Additionally, to model the reduced catalyst surface (Ag/PbO-R), oxygen vacancies (V_O_) were systematically introduced into this baseline structure by varying the vacancy positions at the metal–support interface. The adsorption energies (*E*_ads_) were calculated using eqn (4).

4 *E*_ads_ (eV) = *E*_slab+adsorbate_ − *E*_slab_ − *E*_adsorbate_

## 3. Results and discussion

### 3.1. Physical characterization

The physical characteristics of PbO and Ag/PbO were analyzed using thermogravimetric analysis (TGA), powder X-ray diffraction (PXRD) patterns, pore size distribution, N_2_ adsorption–desorption isotherms, particle size distribution, scanning electron microscope (SEM) images, and transmission electron microscope (TEM) images. The PbO PXRD pattern matched well with the simulated α-PbO^33^ (Fig. S1). The characteristic peaks at 17.7, 28.6, 31.9, 35.8, 48.6, 54.8, 59.9, and 66.5° were comparable to the (100), (101), (110), (002), (112), (211), (202), and (220) lattice planes of α-PbO, respectively^46,47^ (Fig. S1). The characteristic Ag, *i.e.*, (111), (200), and (220), peaks were observed for the Ag/PbO catalysts at 38.1, 44.5, and 64.7°, respectively^48^ (Fig. S1). The Ag peaks became more prominent at higher Ag loadings (Fig. S1). AgNO_3_ impurities were also observed in the 15–40° range for the Ag/PbO catalysts, which also underwent increases in the peak intensities with incremental Ag loading^49^ (Fig. S1). AgNO_3_ impurity peaks were observed consistently in the conditioned catalyst, although they disappeared in the reduced catalyst sample (Fig. S1).

The PbO TGA profile revealed three weight loss regions: (i) 0.4% in the RT-346 °C range, (ii) 0.7% in the 346–681 °C range, and (iii) 0.9% in the 681–800 °C range (Fig. S2). The occurrence of such weight loss events may reflect the involvement of complicated processes (*e.g.*, removal of adsorbed moisture, loss of the H_2_O of crystallization, phase transitions, and structural breakdown) in the thermal degradation of PbO.^50,51^ Similarly, all Ag/PbO catalysts displayed three prominent weight loss events in their individual TGA profiles (Fig. S2). For 0.02%-Ag/PbO, the three weight loss regions were: (i) 1.7% in the RT-383 °C range, (ii) 0.8% in the 383–610 °C range, and (iii) 0.5% in the 610–800 °C range (Fig. S2). For 0.2%-Ag/PbO, the three weight loss regions were: (i) 2.5% in the RT-399 °C range, (ii) 1.9% in the 399–541 °C range, and (iii) 0.4% in the 541–800 °C range (Fig. S2). For 0.9%-Ag/PbO, the three weight loss regions were: (i) 3.3% in the RT-400 °C range, (ii) 4.3% in the 400–515 °C range, and (iii) 0.6% in the 515–800 °C range (Fig. S2). As seen above, the weight loss events were much more pronounced for Ag/PbO catalysts than for PbO (Fig. S2). Also, the weight loss of Ag/PbO catalysts with increasing Ag loading may result from the decomposition and transformation of AgNO_3_ on the catalyst surfaces (Fig. S2). It is crucial to contextualize this weight loss. The TGA was performed under an inert N_2_ atmosphere, and the observed mass loss, particularly in the 100–500 °C range, is primarily attributed to the decomposition of residual AgNO_3_ precursors from the wet impregnation synthesis. This is consistent with the presence of AgNO_3_ impurity peaks in the PXRD patterns of the as-prepared catalysts (Fig. S1). This thermal decomposition under inert conditions does not reflect the structural stability of the final Ag/PbO catalyst under the oxidative atmosphere of the FA oxidation reaction. The true operational stability of the catalyst is instead confirmed by the long-term, 50 h TOS experiment (see Section 3.4), which demonstrates maintained 100% conversion and mineralization under reaction conditions.

All analyzed samples demonstrated type-IV adsorption–desorption isotherms for N_2_, a characteristic feature of mesoporous materials^52^ (Fig. S3). The low/moderate *P*/*P*_0_ regions suggest the complete micropore/mono-/multilayered filling of the mesopores by the N_2_ molecules. The presence of the type-H3 hysteresis loop at high *P*/*P*_0_ for all the N_2_ adsorption–desorption isotherms may imply capillary condensation into the mesopores^52^ (Fig. S3). The presence of a type-H3 hysteresis loop indicates the formation of slit-shaped pores, likely resulting from loosely packed aggregates of plate-like particles.^53^ The presence of mesopores in all the materials was further confirmed by plotting their pore size distribution (Fig. S4). The majority of pores in all the tested materials had diameters greater than 2 nm, although some micropores did exist in the 0.6–2 nm range (Fig. S4). A summary of the Brunauer–Emmett–Teller (BET) surface area and pore volume measurements for the examined materials is provided in Table S1. The surface areas (BET) of the materials were in the 1.5–2.4 m^2^ g^−1^ range, while the values showed a decreasing trend with increasing Ag loading (Table S1). The pore volume of PbO was 0.003 cm^3^ g^−1^, while those of all the Ag/PbO catalysts were identical to 0.002 cm^3^ g^−1^ (Table S1).

SEM of PbO revealed well-faceted crystals, which is in line with those reported earlier^33^ (Fig. S5(a)). For Ag/PbO catalysts, PbO crystals were observed to be covered with irregular Ag aggregates (Fig. S5(b)–(d)). The amount of Ag aggregates on the PbO surface tended to increase progressively with the Ag loading (Fig. S5(b)–(d)). The average particle sizes of the Ag/PbO catalysts (2.1–2.4 µm) were more prominent than PbO (1.9 µm) to show an increasing trend with increasing Ag loading (Fig. S6(a)–(d)). TEM demonstrated that thin PbO sheets were transparent to electrons (Fig. S7). TEM also indicated some stacked PbO sheets (Fig. S7). The PbO sheets were polygonal in shape with smooth edges^54^ (Fig. S7). Well-distributed Ag NPs were observed on the PbO surface for the 0.9%-Ag/PbO catalyst (Fig. S8). The average Ag NP size for 0.9%-Ag/PbO was 8.7 nm (Fig. S9). The observed lattice fringe spacing of 0.151 nm corresponds to the (220) crystallographic plane of Ag^55^ (Fig. S10). Similarly, the lattice fringe spacing of 0.277 nm corresponds to the (200) crystallographic plane of PbO^12^ (Fig. S10).

### 3.2. Surface chemistry

Energy-dispersive X-ray spectroscopy (EDX), Fourier transform infrared spectroscopy (FTIR), and X-ray photoelectron spectroscopy (XPS) were utilized to assess the surface chemistry of the tested materials. In the PbO FTIR spectrum, the characteristic Pb–O stretching and the asymmetric Pb–O–Pb bending vibration bands appeared at 453 and 646 cm^−1^, respectively^46^ (Fig. S11). The minor band at 1411 cm^−1^ was ascribed to the OH bending vibration in adsorbed H_2_O^46^ (Fig. S11). As all the above-described bands were also observed in FTIR spectra of 0.02, 0.2, and 0.9%-Ag/PbO, Ag loading should not have significantly altered the functional groups on the PbO surface (Fig. S11). Interestingly, as the Pb–O–Pb band displayed a blueshift for 0.02, 0.2, and 0.9%-Ag/PbO (compared to PbO), it may possibly indicate the occurrences of interactions with the loaded Ag species (Fig. S11). An AgNO_3_ band was also observed for the Ag/PbO catalysts at ∼1317 cm^−1^ (ref. 56) (Fig. S11). In contrast, the Pb–O–Pb band disappeared for the conditioned and reduced 0.9%-Ag/PbO catalysts (Fig. S11). Also, the OH bending vibration in the adsorbed H_2_O band disappeared for the 0.9%-Ag/PbO catalyst following the reduction pre-treatment (Fig. S11).

EDX mapping was employed to illustrate the elemental distribution across the surfaces of PbO and Ag/PbO catalysts (Fig. S12). As generally expected, the elements Pb, oxygen, and sodium (Na) were detected in all the examined samples (Fig. S12). The usage of NaOH in PbO synthesis was responsible for the observed Na in all samples (Fig. S12). Consistent with expectations, Ag and nitrogen were prominently mapped on the surfaces of the 0.02%, 0.2%, and 0.9%-Ag/PbO samples (Fig. S12). The usage of AgNO_3_ in Ag/PbO synthesis was responsible for the observed nitrogen in all catalyst samples (Fig. S12).

**Fig. 2:**
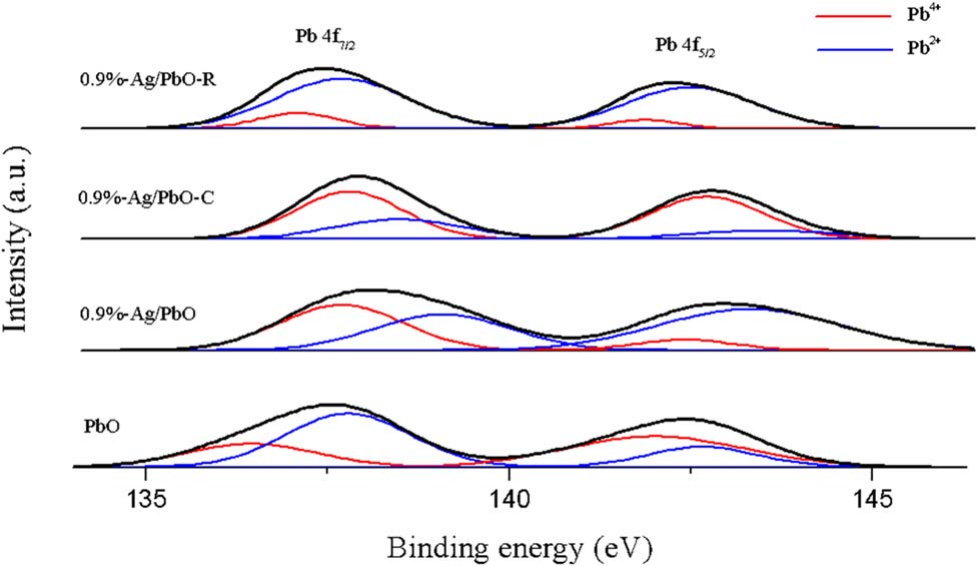
Deconvoluted core-level Pb 4f XPS spectra of the tested materials. Note that 0.9%-Ag/PbO-C is the conditioned catalyst.

The Pb 4f_7/2_ core-level XPS spectrum revealed the presence of Pb^4+^ (136.5 eV) and Pb^2+^ (137.8 eV) species in PbO^57^ (Fig. 2). The proportion of Pb^4+^ in the core-level Pb 4f_7/2_ XPS spectrum rose from 17.4% for PbO to 29.6% in the 0.9%-Ag/PbO sample (Fig. 2). The above observation indicates that the Pb atoms at the Ag–PbO interface lost electrons with Ag loading.^12^ The Pb^4+^ contribution to the Pb 4f_7/2_ core-level XPS spectrum further increased to 37.4% for the conditioned 0.9%-Ag/PbO catalyst (*i.e.*, the sample characterized after N_2_ pre-treatment) (Fig. 2). In contrast, the contribution of Pb^4+^ to the core-level Pb 4f_7/2_ XPS spectrum was only 9% for 0.9%-Ag/PbO-R since electrons were supplied to the Pb atoms at the Ag–PbO interface during the reduction pre-treatment (Fig. 2). Furthermore, the overall Pb 4f spectral envelope for 0.9%-Ag/PbO is shifted to a higher binding energy relative to that of pure PbO. This shift, along with the increased relative contribution of the Pb^4+^ species, indicates a net loss of electron density from the Pb atoms at the Ag–PbO interface (Fig. 2). However, the Pb^4+^ peak in the core-level 0.9%-Ag/PbO-R Pb 4f_7/2_ XPS spectrum underwent a redshift (−0.6 eV) compared to 0.9%-Ag/PbO (Fig. 2). The presence of Pb^4+^ species does not inherently conflict with the Pb^2+^ (PbO) structure identified by PXRD, as XPS only analyzes the surface of the materials under investigation. The above observation was consistent with previous reports.^12^

The analysis of the O 1s core-level XPS spectrum indicated the existence of surface lattice oxygen (O_S-L_ (528.9 eV)), surface oxygen (O^2−^, CO_3_^2−^, and –OH (O_S_ (531.4 eV))), and adsorbed oxygen (H_2_O and organic impurities (O_ads_ (535.7 eV))) in PbO^12^ (Fig. S13). The contribution of O_S-L_ to the O 1s core-level XPS spectrum increased from 18.6% in PbO to 61.6% in the conditioned 0.9%-Ag/PbO catalyst (Fig. S13). In contrast, the contribution of O_S-L_ to the O 1s core-level XPS spectrum was only 39.5% for 0.9%-Ag/PbO-R (Fig. S13). O_S-L_ species have been reported to primarily determine the FA oxidation activity of the Pt/PbO catalysts.^12^ The conditioned 0.9%-Ag/PbO catalyst will likely showcase superior FA oxidation performance over 0.9%-Ag/PbO-R. The O–Ag interactions are suspected to increase the number of O_S-L_ species rather than the O–Pb interactions, as confirmed by the higher O_S-L_ concentrations in the conditioned 0.9%-Ag/PbO and 0.9%-Ag/PbO-R catalysts than PbO (*i.e.*, activation of a significant number of O_S-L_ species near the Ag centers) (Fig. S13).

**Fig. 3:**
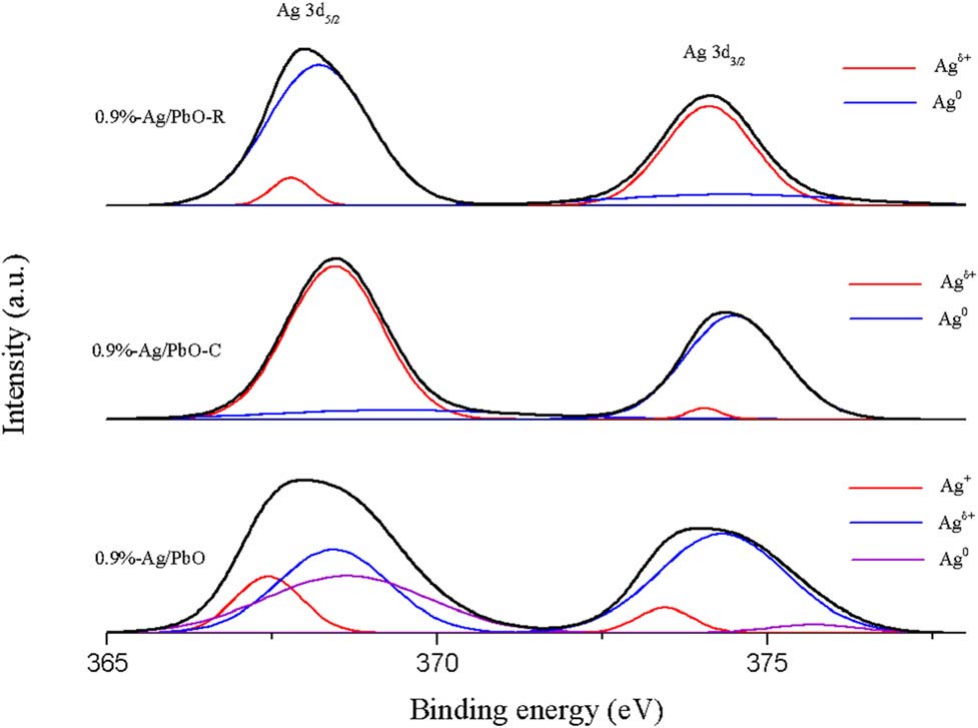
Deconvoluted core-level Ag 3d XPS spectra of the tested materials.

Based on the evaluation of core-level Ag 3d_5/2_ XPS spectrum, the presence of Ag^+^ (367.4 eV), Ag^*δ*+^ (0 < *δ* < 1 (368.4 eV)), and Ag^0^ (368.6 eV) species was identified in 0.9%-Ag/PbO^9^ (Fig. 3). The contributions of Ag^+^, Ag^*δ*+^, and Ag^0^ to the core-level Ag 3d_5/2_ XPS spectrum of 0.9%-Ag/PbO were 10.2, 24.9, and 24.9%, respectively (Fig. 3). In contrast, only the Ag^*δ*+^ (368.4 eV) and Ag^0^ (369.4 eV) species were observed in the core-level Ag 3d_5/2_ XPS spectrum of conditioned 0.9%-Ag/PbO with their contributions of 53.5 and 8%, respectively (Fig. 3). Similarly, both the Ag^0^ (368.2 eV) and Ag^*δ*+^ (367.8 eV) species were observed in the Ag 3d_5/2_ core-level XPS spectrum of 0.9%-Ag/PbO-R with their contributions of 4.1 and 53.2%, respectively (Fig. 3).

The Ag^*δ*+^ peak position in the core-level Ag 3d_5/2_ XPS spectrum of 0.9%-Ag/PbO did not display any shift even after the conditioning (Fig. 3). However, a red-shift (−0.6 eV) was observed in the Ag^*δ*+^ peak position in the core-level Ag 3d_5/2_ XPS spectrum of 0.9%-Ag/PbO-R, likely due to the reduction pre-treatment (Fig. 3). A blue shift (+0.8 eV) was observed in the position of the Ag^0^ peak in the Ag 3d_5/2_ core-level XPS spectrum of the conditioned 0.9%-Ag/PbO (compared to 0.9%-Ag/PbO) to indicate the transfer of electrons from the Ag^0^ sites (Fig. 3). However, a red-shift (−0.4 eV) was observed in the Ag^0^ peak position (compared to 0.9%-Ag/PbO) in the core-level Ag 3d_5/2_ XPS spectrum of 0.9%-Ag/PbO-R (Fig. 3). The results above suggest that both Pb and certain Ag atoms function as electron donors, implying that oxygen at the metal–support interface may act as an electron acceptor to preserve charge balance and play a role in the FA oxidation reaction.^12^

### 3.3. TPR results

The amount of O_S_ used during reduction was 181.9 ± 11.0, 181.8 ± 14.3, and 211.1 ± 3.6 µmol_H2_ for PbO, Ag/PbO, and Ag/PbO-R (averaged with three heating rates), respectively (Table S2). PbO and Ag/PbO had the same oxygen concentration (∼3 mmol_O*_ kg_cat_^−1^). In contrast, Ag/PbO-R had an oxygen concentration of 3.51 mmol_O*_ kg_cat_^−1^, 17% higher than PbO and Ag/PbO. The above results, showing a higher oxygen concentration in Ag/PbO-R *via* TPR, may seem to contradict the XPS data, which indicated lower O_S-L_. However, this apparent discrepancy is resolved by considering the different states of the catalyst during each analysis. Prior to a TPR experiment, each catalyst is pretreated with O_2_. The reduced catalyst (Ag/PbO-R), with its abundance of V_O_ and electron-rich sites, has a much higher affinity for O_2_ than the non-reduced sample. It is therefore likely that the reduced catalyst takes up significantly more O_2_ during this pre-treatment step, yielding an enhanced reserve of highly reducible oxygen species. This explains the higher H_2_ consumption measured by TPR, which reflects the re-oxidation capacity of the catalyst rather than its initial, post-reduction surface state measured by XPS. It is important to note the significant differences in reducing agents between FA oxidation and TPR experiments. FA molecules serve as the reducing agents in the former, whereas H_2_ molecules fulfill this role in the latter. Therefore, it is reasonable to expect varying degrees of catalyst reduction between FA oxidation and H_2_-TPR experiments. A total of nine parameters were estimated to model the experimental TPR data for each heating rate (1.6, 4, and 10 K min^−1^), with three peaks per profile (*N* = 3) and three parameters assigned to each peak, as detailed in the SI. The overall modeling for the TPR data yielded a maximum average error (*e*_max_) of 2.25%, observed for PbO at a heating rate of 1.6 K min^−1^ (eqn (5)). *n* indicates the number of data points per experiment, while *y*_max_ is the maximum output value (a.u.) for each TPR experiment. Due to its complexity, the parameter *S* is defined and explained in greater detail in the SI.5
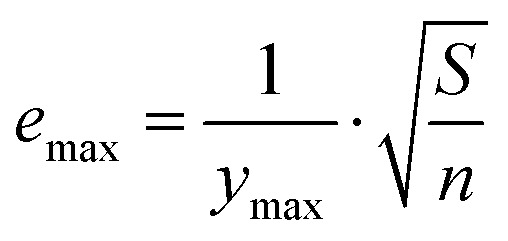


**Fig. 4:**
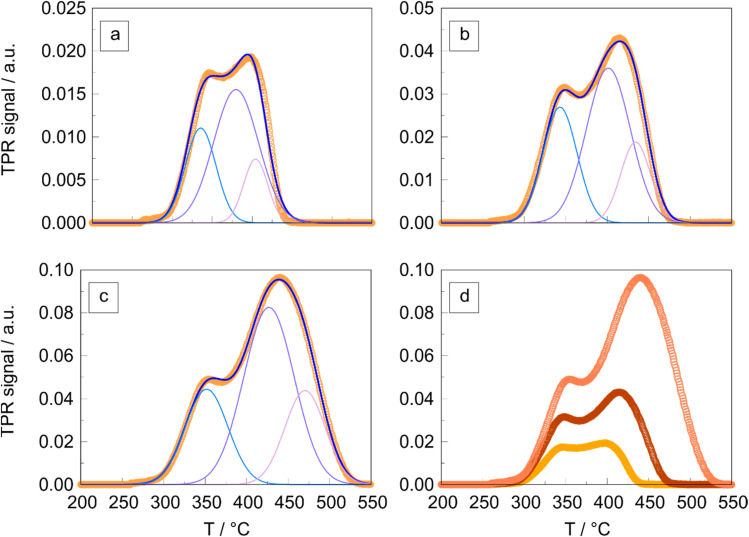
H_2_-TPR profiles of PbO recorded at different heating rates: (a) *β* = 1.6 K min^−1^, (b) *β* = 4 K min^−1^, (c) *β* = 10 K min^−1^, and (d) comparison of the experimental profiles at all three heating rates. The open circles represent the experimental data. In (a)–(c), the solid curves show the Gaussian deconvolution, including three individual Gaussian components and the overall fitted profile.

**Fig. 5:**
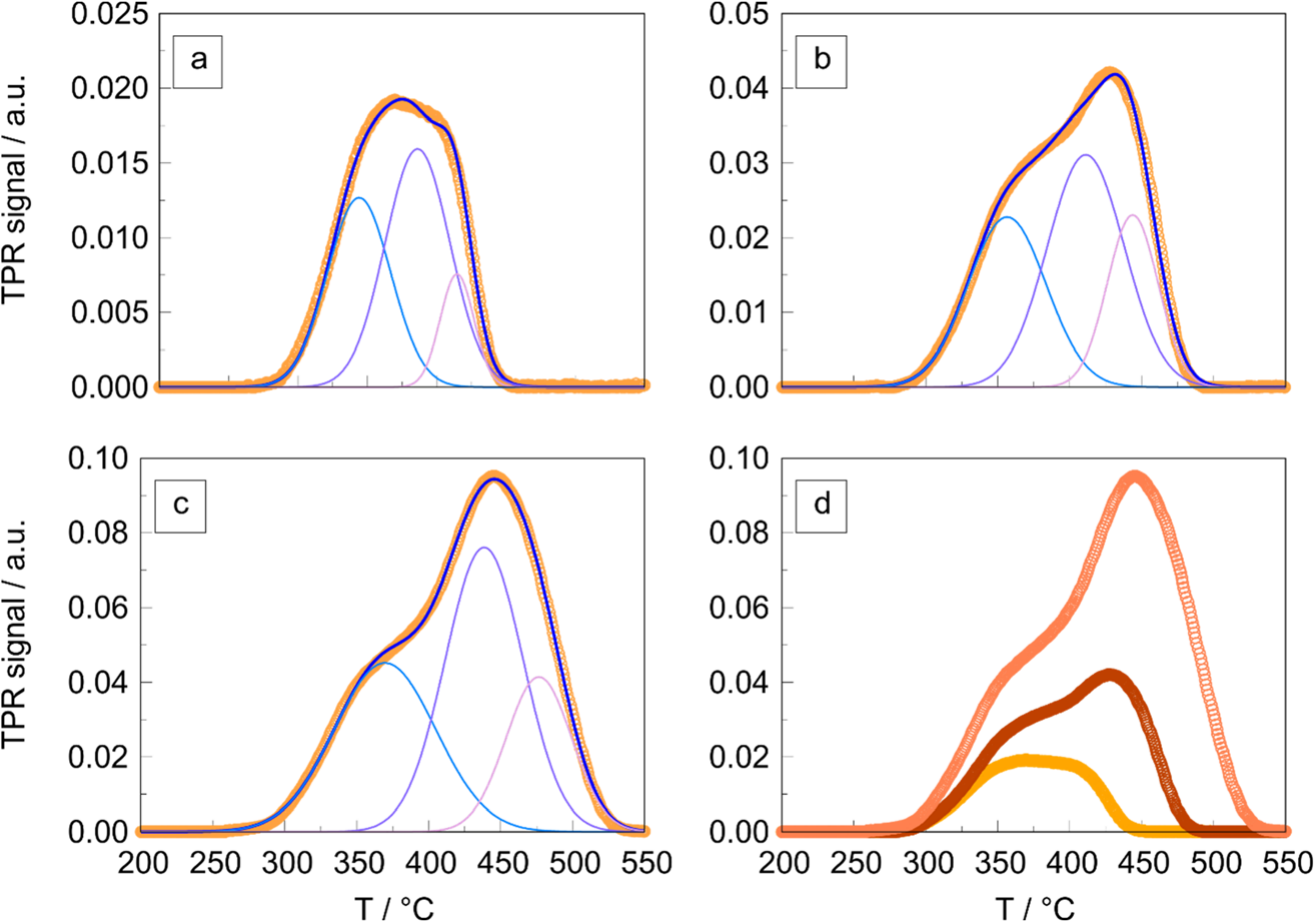
H_2_-TPR profile for 0.9%-Ag/PbO at different heating rates: (a) *β* = 1.6 K min^−1^, (b) *β* = 4 K min^−1^, (c) *β* = 10 K min^−1^, and (d) comparison of the experimental profiles at all three heating rates. The open circles represent the experimental data. In (a)–(c), the solid curves show the Gaussian deconvolution, including three individual Gaussian components and the overall fitted profile.

Table S3 outlines the kinetic parameters for the reduction of PbO, where peaks 1, 2, and 3 accounted for 26.6 ± 3%, 52.5 ± 0.7%, and 20.9 ± 2.3% of the total signal, respectively. Table S4 provides the kinetic parameters for the reduction of Ag/PbO, with peaks 1, 2, and 3 representing 34.4 ± 0.8%, 44.8 ± 0.1%, and 21 ± 0.8% of the total signal, respectively. The first peak for Ag/PbO showed a greater contribution compared to the support, while peak 2 was notably smaller, and the third peak showed no significant change. Table S5 summarizes the kinetic parameters for Ag/PbO-R, where peaks 1, 2, and 3 contributed 50.3 ± 0.4%, 43.5 ± 0.4%, and 6.3 ± 0.7% of the total signal, respectively. The first peak accounted for half of the reducible O_S_, while the third peak, observed at the highest reduction temperature, had a substantially reduced contribution. Fig. 4 presents the H_2_-TPR profile for PbO at the specified heating rates, along with the data fitted using the Gaussian model. Strong agreement was observed between the experimental data and the predicted values. Fig. 5 and S14 show the H_2_-TPR profiles for the Ag/PbO and Ag/PbO-R catalysts, respectively. The regression of the pairs (*T*_max_, *β*) (refer to SI) was used to calculate the activation energy (*E*) and pre-exponential factor (*A*) for each reduction peak (Fig. S15). The *E* values for PbO were ranked as follows: 105.1 ± 2.7 kJ mol^−1^ (peak 1) > 45.7 ± 0.8 kJ mol^−1^ (peak 2) > 36.6 ± 0.6 kJ mol^−1^ (peak 3). It was surprising to observe that the first peak (at lower reduction temperatures) exhibited the highest *E*. The highest *A* (6.97 × 10^13^ s^−1^) was also obtained for the same peak, giving rise to the highest reduction rate (at the lowest temperature).

For Ag/PbO, the *E* for H_2_ reduction followed this order: 69.8 ± 0.4 kJ mol^−1^ (peak 1) > 42.5 ± 0.4 kJ mol^−1^ (peak 2) ≈ 41.3 ± 0.1 kJ mol^−1^ (peak 3). Peaks 2 and 3 showed similar *E*, with *A* being ∼4.2 times higher than peak 1. Based on full kinetic analysis, the reduction coefficient for H_2_-TPR was calculated using the estimates from Tables S4 and S5. This coefficient represents the oxidizing action on the catalyst surface, indicating the availability of O_S_. For the Ag/PbO catalyst, the calculated reduction coefficients at RT were 4.35 × 10^−5^ and 1.82 × 10^−5^ s^−1^ for the first and second O_S_ species, respectively. For Ag/PbO-R, the same values were 5.77 10^−6^ and 8.36 10^−7^ s^−1^. Based on the above results, it can be inferred that the Ag/PbO catalyst demonstrates a higher oxidation rate due to comparable O_S_ availability under operational conditions (FA oxidation in the presence of O_2_), which contributes to improved FA oxidation performance. If a reduction coefficient is represented using the activated complex theory, the ratio of the two coefficients with comparable *E* values represents the entropy difference (Δ*S*^≠^)^58^ (eqn (6) and (7)).6
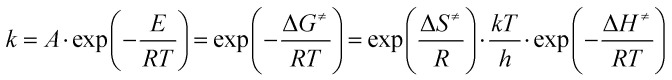
7
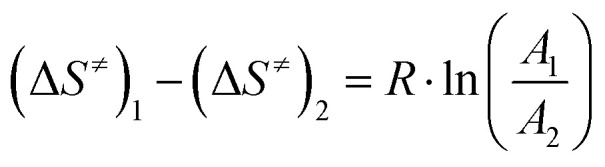


Δ*S*^≠^ denotes the difference in entropy between the activated complex and the reactants (H_2_ and O*). The significance of *x*^≠^ lies in its role in influencing the equilibrium shift between the reactants and activated complexes, as described by the standard thermodynamic equation (Δ*G*^≠^ = Δ*H*^≠^ − *T*·Δ*S*^≠^). The *n*^≠^ value can be approximated by the *E*, with a maximal deviation of 10%. An increase in Δ*S*^≠^ results in a decrease in *b*^≠^, thereby shifting the equilibrium toward the formation of the activated complex in the surface reduction reaction. Using the results for the *A* presented in Table S4, the difference in Δ*S*^≠^ for the surface reduction reaction amounts to 11.9 J mol^−1^ K^−1^. For Ag/PbO, a higher Δ*S*^≠^ value was obtained for peak 2 (compared to peak 3). Hence, it can be assumed that the corresponding activated complex for the surface H_2_ reduction reaction is more loosely bonded to the Ag/PbO catalyst surface, giving rise to higher activity in reduction processes when peak 2 is compared to peak 3. In other words, the intermediate complex is easily converted into the final product (H_2_O), resulting in high catalytic activity.

The relative ordering of *E* values for Ag/PbO-R can be listed as 58.4 ± 0.3 (peak 1) ≈ 61.5 ± 0.9 (peak 2) > 36.1 ± 0.5 (peak 3) kJ mol^−1^. Peaks 1 and 2 had a similar *E*. However, *A* for the former peak was two times higher than the latter, with their difference in Δ*S*^≠^ as 5.7 J mol^−1^ K^−1^. In catalysis, a linear relationship between ln *A* and the corresponding *E* is often observed for the same class of reactions or the same reaction over similar materials (compensation effect). Such a concept helps express interrelated kinetic behavior within a group of rate processes.^59^ The plot between ln A and *E* for the analyzed catalysts confirms a well-fitting linear relationship (Fig. S16). As the compensation effect is widely studied and has relatively little explanation in the literature, the above observation is reported without further explanation. It is noted that the integrated peak areas in the H_2_-TPR profiles (Fig. 4 and 5) appear to increase with the heating rate. This is a known experimental artifact in TPR analysis, often attributed to heat transfer limitations and baseline correction complexities at faster temperature ramps.

### 3.4. Catalytic activity measurements

**Fig. 6:**
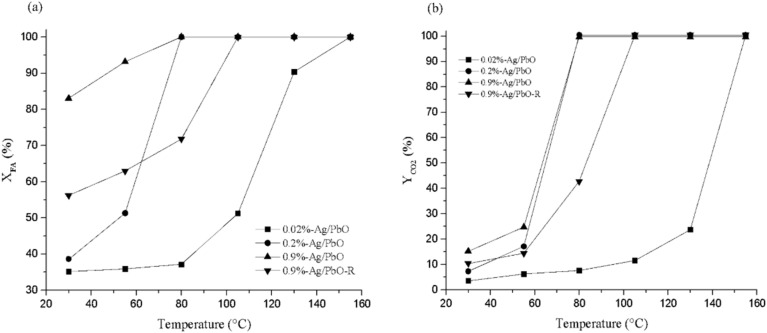
FA removal performance of Ag/PbO catalysts: (a) light-off curve (FA concentration: 100 ppm, catalyst mass = 150 mg, flow rate = 50 mL min^−1^, and RH = 0%). (b) *Y*_CO_2__ as a function of temperature (FA concentration: 100 ppm, catalyst mass = 150 mg, flow rate = 50 mL min^−1^, and RH = 0%). The *T*_50_ value for 0.9%-Ag/PbO is an extrapolated estimate, as conversion at the lowest experimental temperature (30 °C) was 83%.

The steady-state performance of all the tested catalysts for FA oxidation at RT followed this order of decreasing efficiency: 0.9%-Ag/PbO > 0.9%-Ag/PbO-R > 0.2%-Ag/PbO > 0.02%-Ag/PbO (Fig. 6(a)). At RT (30 °C), the initial *X*_FA_ for the 0.9%-Ag/PbO catalyst was already 83%, significantly higher than for the other catalysts (56%, 39%, and 35%) (Fig. 6(a)). The temperatures required to reach 50% conversion (*T*_50_) were 93, 52, and 103 °C for the 0.9%-Ag/PbO-R, 0.2%-Ag/PbO, and 0.02%-Ag/PbO catalysts, respectively. For the highly active 0.9%-Ag/PbO catalyst, since conversion already exceeded 50% at the starting temperature, the *T*_50_ is estimated by extrapolation to be approximately 18 °C (Fig. 6(a)). Their reaction temperature values corresponding to 90% *X*_FA_ (*T*_90_) were 47, 96, 75, and 130 °C, respectively (Fig. 6(a)). Therefore, catalytic activity improves with an increase in Ag loading from 0.02% to 0.9%. Also, the conditioned catalyst outperformed the reduced catalyst, likely due to the abundance of O_S-L_ and Ag^*δ*+^ species in the former (see Section 3.2).

Ag^*δ*+^ was reported to outperform Ag^0^ in the FA oxidation reaction,^9^ which is consistent with the findings of the present work. A steady rise in both the *X*_FA_ and *Y*_CO_2__ values with temperature is also in line with general expectations (Fig. 6(a) and (b)). Furthermore, the attainment of 100% *X*_FA_ and *Y*_CO_2__ values at the same temperature indicates complete FA mineralization per the carbon balance (Fig. 6(a) and (b)). However, FA mineralization proceeded partially at lower temperatures, as the *Y*_CO_2__ values were smaller than *X*_FA_ (Fig. 6(a) and (b)). This discrepancy is particularly notable at lower temperatures. For example, the 0.9%-Ag/PbO catalyst under dry, RT conditions showed an *X*_FA_ of 83% but a *Y*_CO_2__ of only 15%. This substantial ∼68% point gap in carbon balance strongly indicates that the majority of converted FA is being retained on the catalyst surface as strongly-bound intermediates. This is fully consistent with our *in situ* DRIFTS results (see Section 3.5.1), which show the rapid formation and accumulation of dioxymethylene (DOM) and carbonate (CO_3_^2−^) species at low temperatures. These surface species are evidently stable under these conditions and only undergo complete oxidation to CO_2_ as the temperature increases, which is why the *X*_FA_ and *Y*_CO_2__ curves converge at higher temperatures.

It is also important to address the possibility of other gas-phase byproducts, such as CO. Our GC-methanizer-FID setup co-detects CO and CO_2_, meaning any CO produced would be counted in the *Y*_CO_2__ value. However, we assume CO formation to be negligible for several reasons. First, no gas-phase or significant surface-adsorbed CO was detected as a stable product during any of our *in situ* DRIFTS experiments. Second, the reaction is conducted in a large excess of O_2_ (21 vol%), which thermodynamically favors complete oxidation. Finally, post-reaction XPS analysis confirms a drastic consumption of O_S-L_ (see Section 3.5.1), indicating that the catalyst maintains a highly oxidative surface state during the reaction, which would be expected to rapidly oxidize any potential CO intermediate to CO_2_. Therefore, the observed carbon gap is confidently attributed to the accumulation of surface-bound intermediates rather than the formation of gas-phase CO. The 0.9%-Ag/PbO catalyst, which showed the best performance, was chosen for further investigation to analyze how process variables influence FA oxidation efficiency.

**Fig. 7:**
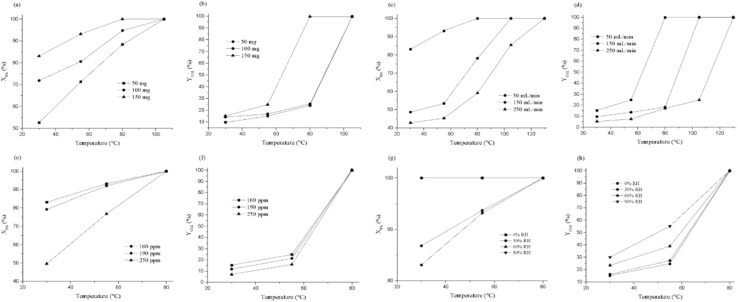
FA removal performance of 0.9%-Ag/PbO: (a) light-off curve (FA concentration: 100 ppm, catalyst mass = 50–150 mg, flow rate = 50 mL min^−1^, and RH = 0%). (b) *Y*_CO_2__ as a function of temperature (FA concentration: 100 ppm, catalyst mass = 50–150 mg, flow rate = 50 mL min^−1^, and RH = 0%). (c) light-off curve (FA concentration: 100 ppm, catalyst mass = 150 mg, flow rate = 50–150 mL min^−1^, and RH = 0%). (d) *Y*_CO_2__ as a function of temperature (FA concentration: 100 ppm, catalyst mass = 150 mg, flow rate = 50–150 mL min^−1^, and RH = 0%). (e) Light-off curve (FA concentration: 100–250 ppm, catalyst mass = 150 mg, flow rate = 50 mL min^−1^, and RH = 0%). (f) *Y*_CO_2__ as a function of temperature (FA concentration: 100–250 ppm, catalyst mass = 150 mg, flow rate = 50 mL min^−1^, and RH = 0%). (g) Light-off curve (FA concentration: 100 ppm, catalyst mass = 150 mg, flow rate = 50 mL min^−1^, and RH = 0–90%). It is crucial to note that this 100% conversion at RT does not represent complete oxidation. As shown in (h), the corresponding CO_2_ yield at RT is only 24–30%, indicating that the majority of removed FA remains on the surface as intermediates (confirmed by DRIFTS in Section 3.5.1). (h) *Y*_CO_2__ as a function of temperature (FA concentration: 100 ppm, catalyst mass = 150 mg, flow rate = 50 mL min^−1^, and RH = 0–90%).

RT *X*_FA_ decreased as a function of *m*_cat_ in the following order: 83% (150 mg) > 72% (100 mg) > 53% (50 mg) (Fig. 7(a)). Similar patterns were also seen from the *Y*_CO_2__ at RT: 15% (150 mg) > 14% (100 mg) > 10% (50 mg) (Fig. 7(b)). The decrease in FA oxidation performance with a reduction in *m*_cat_ was expected, as it directly correlates with diminished availability of active surface sites for the reaction. With an increase in the flow rate (mL min^−1^), the *X*_FA_ at RT declined in the following sequence: 50 (83%) > 150 (49%) > 250 (43%) (Fig. 7(c)). Likewise, the *Y*_CO_2__ at RT also exhibited comparable patterns with the flow rate (mL min^−1^): 50 (15%) > 150 (10%) > 250 (5%) (Fig. 7(d)). The decline in FA oxidation performance with a higher flow rate was reasonable, as it directly reduces the residence time (*τ*) or the contact duration between FA molecules and the catalyst within the reactor.

RT *X*_FA_ lowered in the following sequence with the rise in FA concentration: 83% (100 ppm) > 79% (190 ppm) > 50% (250 ppm) (Fig. 7(e)). Similarly, *Y*_CO_2__ at RT exhibited comparable patterns with the changes in FA concentration: 15% (100 ppm) > 12% (190 ppm) > 7% (250 ppm) (Fig. 7(f)). The decline in FA oxidation performance with rising FA concentration was logical, as active surface sites can become saturated more quickly due to the higher influx of FA molecules. RT *X*_FA_ increased in the following sequence with increasing RH level: 0% RH (83%) > 30% RH (87%) > 60% RH (100%) = 90% RH (100%) (Fig. 7(g)). *Y*_CO_2__ at RT also shared a similar pattern: 0% RH (15%) > 30% RH (16%) > 60% RH (24%) > 90% RH (30%) (Fig. 7(h)). Enhanced catalytic activity in the co-presence of moisture makes the Ag/PbO catalyst robust for practical applications due to the significant presence of H_2_O vapor under practical conditions.

Although 100% *X*_FA_ could be attained at RT for RH levels ≥60%, 100% *Y*_CO_2__ could only be reached at 80 °C (under dry and moist conditions) (Fig. 7(g) and (h)). Such observations indicate that although moisture enhances FA mineralization slightly at lower temperatures, enhanced *X*_FA_ may be ascribable to heightened transformative capture of the FA molecules by the PbO surface. FA adsorption experiments conducted at RT using PbO in the absence and presence of moisture (90% RH) were useful in verifying such a hypothesis, as described earlier (see Section 2.3). Fig. S17(a) illustrates that FA adsorption is more pronounced in the presence of moisture, as evidenced by the gentler slope of the BT profile compared to dry conditions. A sharper BT profile suggests insufficient intraparticle resistance to adsorbate mass transfer, leading to faster saturation of active surface sites and reduced adsorption efficiency.

Enhanced adsorption of FA molecules in the presence of moisture by PbO is also supported by the observation that the 10% BT volume value was 3.3 times higher under the moist condition than under the dry condition (0.1 *versus* 0.03 L atm g^−1^) (Fig. S17(a)). Similarly, the maximum *q* doubled under the moist condition than under the dry condition (0.06 *versus* 0.03 mg g^−1^) to indicate enhanced FA adsorption in the co-presence of H_2_O vapor by PbO (Fig. S17(b)). In the co-presence of moisture, FA molecules should interact with H_2_O on the PbO surface to become hydrated (CH_2_(OH)_2_) for reactive adsorption through the formation of PbCH_2_O_2_ (ring-like species).^60^ The remarkable stability of the Ag/PbO catalyst is demonstrated by maintaining 100% *X*_FA_ to CO_2_ at RT for an extended testing period of 50 h TOS under optimized process conditions (90% RH) (Fig. S18).

### 3.5. FA oxidation mechanism and surface phenomena

#### 3.5.1. *In situ* DRIFTS and physicochemical characterization

The main function of the support is to promote FA adsorption, enabling the subsequent oxidation process.^16,61^ The FA–PbO interaction was studied using the FTIR spectrum after FA adsorption under dry and moist conditions (Fig. S19). New bands appeared at 778, 882, 1329, 1427, and 1647 cm^−1^ after FA adsorption under both conditions compared to fresh PbO (Fig. S11 and S19). The 778 and 882 cm^−1^ bands were ascribed to Pb–O–C stretching vibration^60^ (Fig. S19). The 1329 and 1427 cm^−1^ bands were attributed to the bending vibration of the CH_2_ group^60^ (Fig. S19). The 1647 cm^−1^ band was attributed to the stretching vibration of the C=O group^62^ (Fig. S19). Interestingly, the 1572 cm^−1^ band (COO) was only observed in the case of FA adsorption under moist conditions^60^ (Fig. S19). The appearance of the COO band under such conditions suggests that FA should adsorb on the PbO surface through the formation of ring-like PbCH_2_O_2_*via* CH_2_(OH)_2_ (ref. 12 and 60) (Fig. S19). In contrast, the disappearance of the Pb–O–Pb band (651 cm^−1^) after FA adsorption under the dry condition indicates that the FA molecules could have been reactively adsorbed on the surface of PbO (Fig. S19).

**Fig. 8:**
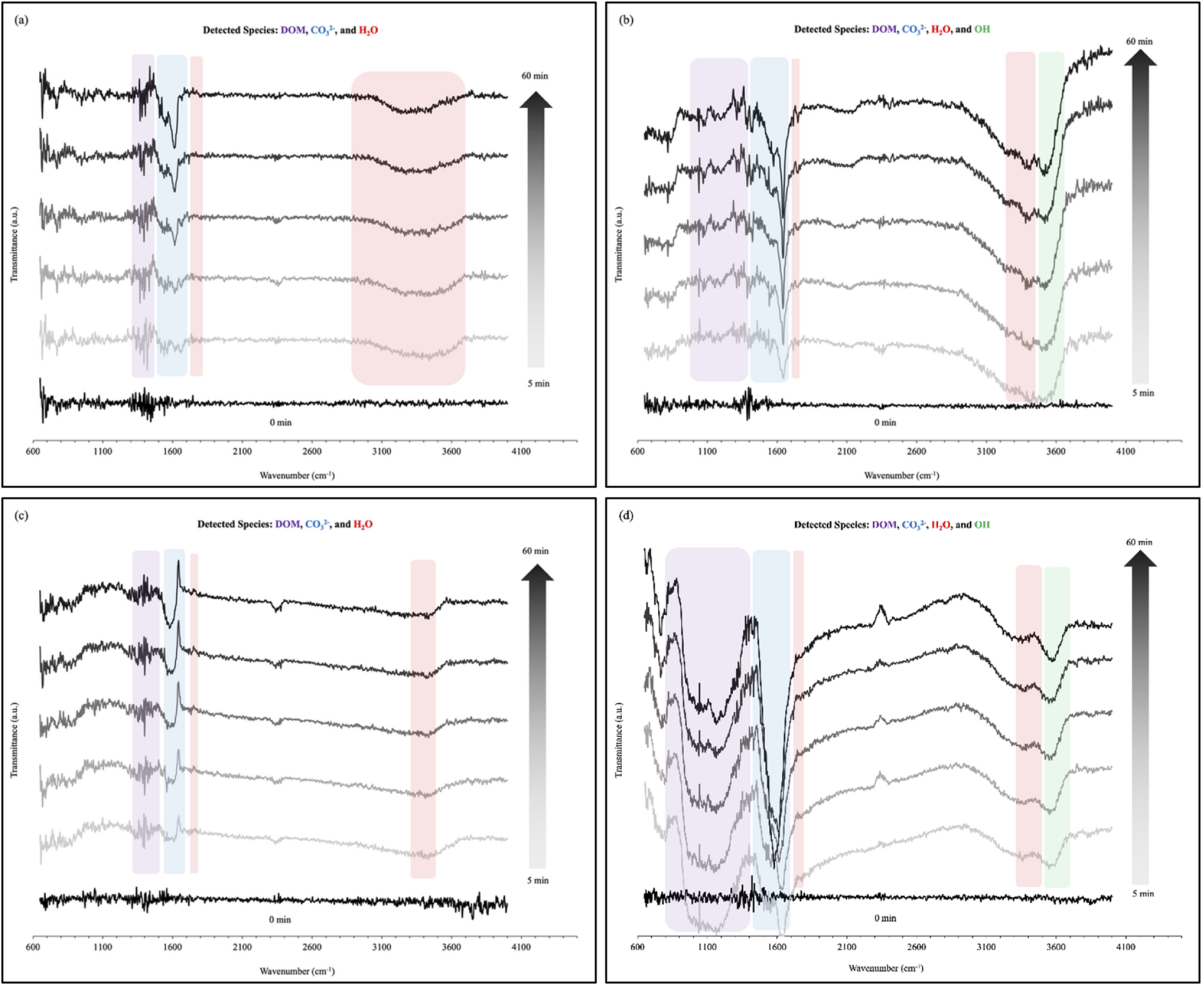
*In situ* DRIFTS spectrum (100 °C) for 0.9%-Ag/PbO. Panel (a) FA + air. Panel (b) FA + air + H_2_O. Panel (c) FA + N_2_. Panel (d) FA + N_2_ + H_2_O.

The FA oxidation pathway on the Ag/PbO (Fig. 8) and Ag/PbO-R (Fig. S20) surfaces was determined using *in situ* DRIFTS. *In situ* DRIFTS spectra for Ag/PbO during FA oxidation under different atmospheric conditions (air (Fig. 8(a)), air + H_2_O (Fig. 8(b)), N_2_ (Fig. 8(c)), and N_2_ + H_2_O (Fig. 8(d))) revealed the presence of the following species: (i) DOM (1110/1117 cm^−1^ (*ν*(CO))), 1310/1324/1360/1382/1386/1396 cm^−1^ (*ν*(CO)/*ω*(CH_2_)/*τ*(CH_2_)), and 1415/1447/1458/1485 cm^−1^ (*δ*(CH_2_)), (ii) CO_3_^2−^ (1574/1580/1582/1608/1638/1644 cm^−1^), (iii) H_2_O (1664/1730/1748/1753 cm^−1^ (*δ*(H_2_O)) and 3401/3415/3421/3444 cm^−1^ (*ν*(OH))), and (iv) OH (3531 and 3579 cm^−1^). Likewise, *in situ* DRIFTS spectra for Ag/PbO-R during FA oxidation under different atmospheric conditions (air (Fig. S20(a)) and N_2_ (Fig. S20(b))) exhibited the presence of the following species: (i) DOM (1271/1327/1330 cm^−1^ (*ν*(CO)/*ω*(CH_2_)/*τ*(CH_2_))) and 1419/1427 cm^−1^ (*δ*(CH_2_)), (ii) CO_3_^2−^ (1491/1508/1561/1564 cm^−1^), and (iii) H_2_O (196/1735 cm^−1^ (*δ*(H_2_O)) and 3388/3458 cm^−1^ (*ν*(OH))).

These findings suggest that FA molecules initially adhere to the PbO surface before undergoing conversion into DOM. DOM then breaks down into CO_3_^2−^ and H_2_O by interacting with the O_S-L_ species. The formed CO_3_^2−^ was further decomposed into the CO_2_ end product. The appearance of a broad band (3415 cm^−1^) for adsorbed H_2_O at higher wavenumbers without the signals for OH groups indicates non-OH-assisted FA adsorption in the absence of moisture in the air (Fig. 8(a)). Also, the absence of surface OH bands further indicates that the formed H_2_O end-product was not dissociated into surface OH species (Fig. 8(a)). The CO_3_^2−^ bands became more prominent when moisture was present in the air (Fig. 8(b)). Also, a distinct band for surface OH species was observed in the co-presence of moisture at 3531 cm^−1^ (Fig. 8(b)). The above results indicate that moisture is decomposed into surface OH groups. The adsorbed FA molecules then hydrate into CH_2_(OH)_2_ by combining with the surface OH groups to yield PbCH_2_O_2_ (ring-like species).^60^ Also, PbCH_2_O_2_ may help enhance FA oxidation performance through the formation of CO_3_^2−^ species.

The occurrence of DOM, CO_3_^2−^, and H_2_O bands in the N_2_ atmosphere suggests that the O_S-L_ species in Ag/PbO catalysts remain sufficiently active, facilitated by strong Ag–PbO interactions, to take part in the FA oxidation reaction (Fig. 8(c)). However, the absence of O_2_ in the N_2_ atmosphere will eventually deactivate the catalyst as consumed O_S-L_ cannot be replenished. The appearance of the surface OH band (3579 cm^−1^) and increased CO_3_^2−^ band intensity in the moist N_2_ atmosphere further indicate the promotional role of moisture in FA adsorption and conversion through the formation of ring-like PbCH_2_O_2_ (Fig. 8(d)). Furthermore, the negative transmittance bands for surface OH species confirm their consumption during the reaction in the presence of moisture. While this observation experimentally verifies the direct involvement of OH, it does not by itself distinguish their role in hydration *versus* direct oxidation. However, when viewed in conjunction with our DFT calculations (see Section 3.5.2), which demonstrate a clear energetic preference for the FA hydration–oxidation pathway, the evidence strongly supports a mechanism where moisture facilitates the formation of a key CH_2_(OH)_2_ intermediate rather than participating in direct oxidation.^14,60^ Also, the absence of any new species in Ag/PbO-R DRIFTS spectra under all conditions (O_2_ present (Fig. S20(a)) and O_2_ absent (Fig. S20(b))) indicates that Ag species only activate O_2_ and do not alter the FA oxidation pathway. Furthermore, O_2_ activation replenishes the consumed O_S-L_ to sustain the catalytic cycle. Based on the FA oxidation performance (see Section 3.4) and previous results,^5,9^ it is assumed that the Ag^*δ*+^ species has the potential to activate O_2_ at lower temperatures. In contrast, Ag^0^ species require higher temperatures for activating the O_2_ molecules.

Consistency in the Ag/PbO PXRD pattern and FTIR spectrum (*i.e.*, comparison before and after the catalytic reaction) supports its high stability (Fig. S1, S11, S21, and S22). The Pb^4+^ contribution to the core-level Pb 4f_7/2_ Ag/PbO XPS spectrum, when compared between before and after the FA oxidation reaction, was 37.4 and 15.9%, respectively (Fig. 2 and S23). The O_S-L_ contribution to the core-level O 1s XPS spectrum of Ag/PbO was 61.6% before and 12.9% after the FA oxidation reaction, indicating its significant consumption during the process (Fig. S13 and S24). O_S_/O_ads_ contributions to the O 1s core-level Ag/PbO spectrum, when compared before and after the FA oxidation reaction, were 1.7/36.7 and 45.2/41.9%, respectively (Fig. S13 and S24). These findings suggest that O_2_ and H_2_O molecules adsorb and activate on the catalyst surface. The O_S-L_ peak in the core-level O 1s XPS spectrum of Ag/PbO exhibited a redshift of −2.8 eV following the FA oxidation reaction (Fig. S13 and S24), indicating electron gain by the O_S-L_ species during the process. Additionally, the Ag^*δ*s^ contribution to the core-level Ag 3d_5/2_ XPS spectrum of Ag/PbO decreased from 53.5% before the reaction to 24.3% after FA oxidation (Fig. 3 and S25). The Ag^0^ contribution to the core-level Ag 3d_5/2_ Ag/PbO XPS spectrum before and after the FA oxidation reaction was 8 and 34.3%, respectively (Fig. 3 and S25).

#### 3.5.2. DFT simulation

**Fig. 9:**
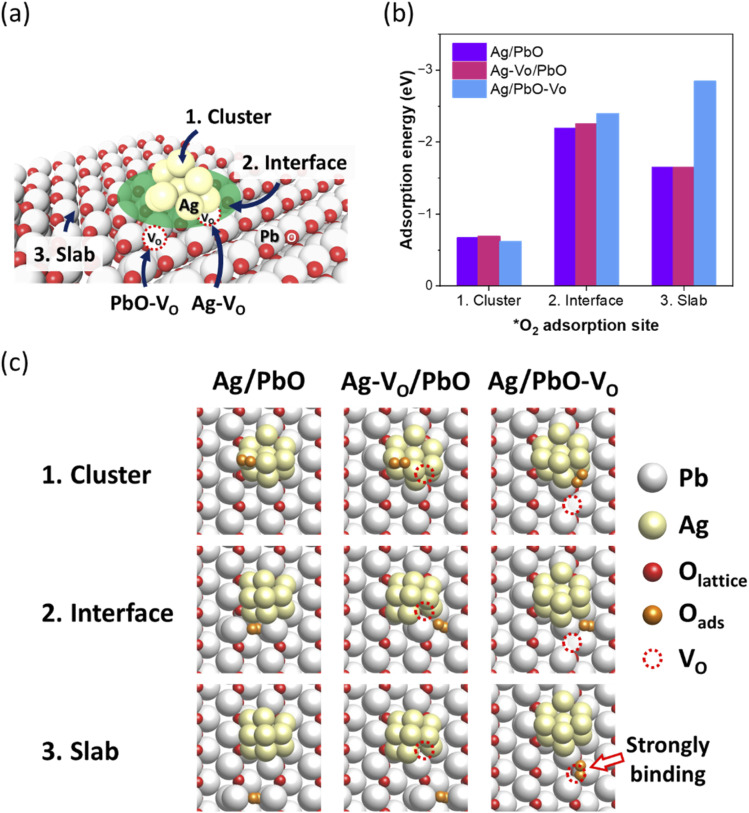
DFT simulation results. Panel (a) presents a schematic of cluster models for Ag/PbO and Ag/PbO-R and the designated adsorption site. Panel (b) shows the adsorption trends in O_2_. Panel (c) depicts the geometries at each reaction site. The interface site represents the region where oxygen can bind simultaneously to both Ag and Pb.

DFT simulations were conducted to gain insight into the surface interactions involved in FA oxidation on Ag/PbO and Ag/PbO-R. The Ag_10_ nanocluster on a PbO (100) slab was utilized as a validated, computationally efficient minimum structural unit to investigate the interfacial phenomena observed experimentally *via* TEM for the supported Ag NPs (see Section 2.4). Based on this representative Ag_10_/PbO baseline structure, the contribution of V_O_ (present on the Ag/PbO-R surface) in O_2_ adsorption was first studied. V_O_ was assumed to be uniformly distributed across the catalyst surface and was categorized based on its location relative to the Ag cluster (Fig. 9(a) and S26(a)–(c)): (i) regions where the V_O_ and cluster do not interact (PbO slab), (ii) regions where V_O_ is buried below the Ag cluster and can only affect the charge state of the cluster (Ag-V_O_/PbO), and (iii) regions where V_O_ is exposed near the cluster, providing an active site to directly affect the charge state of the cluster (Ag/PbO-V_O_). The above category was designed to explain the performance difference between Ag/PbO and Ag/PbO-R based on the distribution of O_S_ as associated with adsorption trends.

At the interface site, there was strong binding between Ag and oxygen, which led to an abundant generation of active oxygen (*O) in the vicinity of Ag clusters (Fig. 8(c), 9(b), and S27). As a result, reactions involving *O and reactive mobile gases are expected to occur mainly in the interfacial region (Fig. 9(c) and S26(d)). Oxygen species (O_2_ and O) strongly bind to exposed V_O_ located at the slab site to yield a defect-free PbO lattice (Fig. 9(b) and (c)). Oxygen species acted as acceptors for excess electrons generated by V_O_ (thus restoring Ag/PbO-V_O_ to Ag/PbO) (Fig. S26(d)). For the Ag-V_O_/PbO case in which V_O_ was located below the cluster, Ag atoms with more electrons (reduced by V_O_) interact more strongly with oxygen species at the cluster and interfacial sites. However, the above bond between the Ag and oxygen species was significantly weaker than the direct occupation of the V_O_ site, indicating that oxygen species are preferentially utilized to compensate for V_O_ defects in the PbO lattice. Consequently, in an environment with a limited supply of O_2_, *O is more prevalent in Ag/PbO than in Ag/PbO-R. This observation suggests a higher concentration of *O on Ag/PbO, as is predominantly required for the FA oxidation process, which aligns with the characterization results (see Section 3.2).

**Fig. 10:**
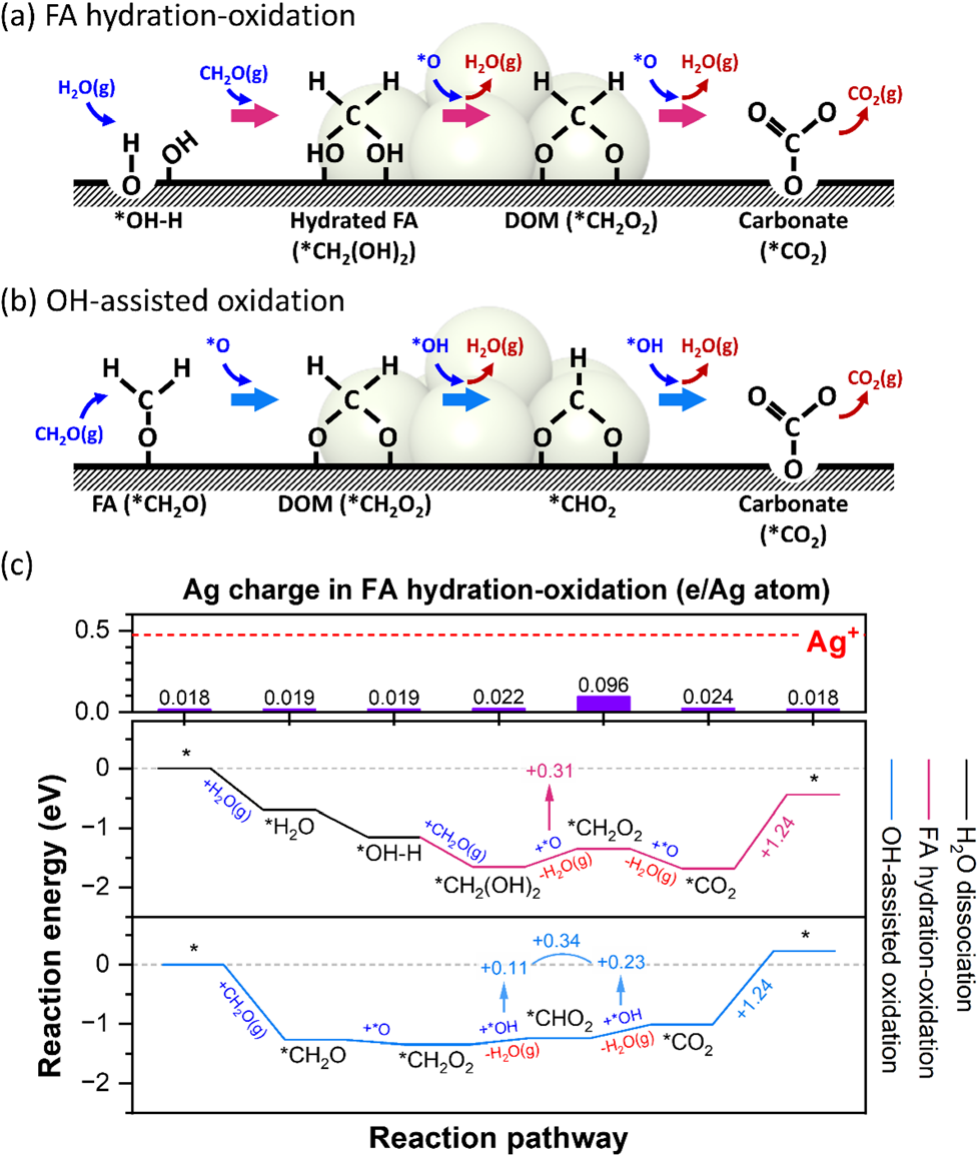
DFT simulation results. Panel (a) is a schematic diagram of FA oxidation on the Ag/PbO surface with hydration supported by moisture. Panel (b) is a schematic diagram of direct FA oxidation without hydration. Panel (c) is the corresponding reaction diagram and Bader charge profile of the Ag cluster at each step of the FA hydration–oxidation pathway.

The reaction preference was identified to investigate how FA oxidation energetics change in the presence of moisture. Two possible pathways involving *OH (generated from H_2_O decomposition) were considered: one where *OH is utilized to form hydrated FA to subsequently undergo oxidation (FA hydration–oxidation pathway) and the other where *OH directly participates in the oxidation reaction (OH-assisted oxidation pathway). It was assumed that *O and *OH were abundant around the Ag cluster (Fig. 10(a) and (b)), resulting from the spontaneous activation of O_2_ and H_2_O on the Ag/PbO surface, as indicated by the downhill black line in Fig. 10(c). Similar to how, as previously described, O_2_ activation occurred favorably around the Ag cluster, the FA oxidation reaction was also expected to occur predominantly at the interfacial site. The FA molecule was strongly bound to *OH at the interfacial sites to form *CH_2_(OH)_2_ (Fig. S28). Subsequently, a reaction occurs with *O to yield *CH_2_O_2_ (DOM) by releasing H_2_O(g) with a minor energy barrier of 0.31 eV. If *OH was directly involved in the reaction, FA oxidation would have proceeded *via* *CHO_2_ (blue line in Fig. 10(c)). However, the above reaction was unfavorable, with a barrier of 0.34 eV. Therefore, the generated *OH spontaneously hydrated FA but did not directly participate in FA oxidation. Based on the computed energy profile for the favored FA hydration–oxidation pathway, the apparent activation energy for the overall FA oxidation is determined to be 1.24 eV, which corresponds to the rate-determining step of the final CO_2_ desorption (*CO_2_ → * + CO_2_(g)). In addition, no significant changes in the oxidation state of the Ag cluster were observed with the progression of the FA hydration–oxidation pathway. The experimental results (Fig. S25) support the reduction of the Ag cluster by excess electrons generated by V_O_. The reduction was more significant when V_O_ was located close to Ag (Ag-V_O_/PbO) due to the attraction of electrons by the *O acceptor from neighboring cations (Pb and Ag). Hence, *O consumption (desorption) in the interfacial region during FA oxidation should reduce the oxidation state of Ag.

## 4. FA oxidation performance comparison

The *r* determined at 80 °C served as the key benchmark for evaluating the performance of different supported Ag catalysts reported for FA oxidation in the presence of air (Table 1). The use of *r* to assess catalytic performance is meaningful as it quantitatively integrates *X*_FA_, *F*_FA_, and *m*_cat_ in a single term. In this regard, an Ag-potassium (K)/Al_2_O_3_ catalyst was observed to be the best performer with the *r* of 379 µmol g^−1^ h^−1^ (Table 1). For the other catalysts, the *r* ranged from 26 (0.02%-Ag/PbO) to 322 µmol g^−1^ h^−1^ (Ag/TiO_2_ (anatase)) (Table 1). The 0.2 and 0.9%-Ag/PbO catalysts displayed the same *r* of 69 µmol g^−1^ h^−1^ (Table 1). The 0.9%-Ag/PbO-R displayed an *r* of 50 µmol g^−1^ h^−1^ (Table 1).

**Table 1:** FA removal performance comparison of selected supported Ag catalysts under DLT conditions

Order	Catalyst	Ag (wt%)	*m*_cat_ (mg)	BET surface area (m^2^ g^−1^)	Catalyst activation	Reactant composition	FA concentration (ppm)	Flow rate (mL min^−1^)	*F*_FA_ (mol s^−1^)	Space velocity	*T*_50_ (°C)	*T*_90_ (°C)	*X*_FA_ (%) at 80 °C	*r* a (µmol g^−1^ h^−1^)	Ag normalized *r* (mmol g^−1^ h^−1^)	*r*_BET_ (µmol m^−2^ h^−1^)	TOF (mmol mol^−1^ s^−1^)	References
1	Ag–K/Al_2_O_3_	7.77	60	127.9	Calcination at 450 °C for 3 h	FA + O_2_ (20 vol%) + He (balance)	110	100	6.3 × 10^−9^	100 000 mL h^−1^ g^−1^	38	54	100	379	4.9	2.96	22.2	24
2	Ag/TiO_2_ (anatase)	8	60	35.3	Calcination at 450 °C (5 °C min^−1^) in static air for 3 h	FA + O_2_ (20 vol%) + N_2_ (balance)	110	100	6.3 × 10^−9^	100 000 mL h^−1^ g^−1^	65	87	85	322	4	9.12	11.6	63
3	Ag/Al_2_O_3_	8	60	154.2	Calcination at 450 °C (5 °C min^−1^) in static air for 3 h	FA + O_2_ (20 vol%) + N_2_ (balance)	110	100	6.3 × 10^−9^	100 000 mL h^−1^ g^−1^	73	103	65	247	3.1	1.6	30.2	63
4	Ag/TiO_2_ (rutile)	8	60	20	Calcination at 450 °C for 3 h	FA + O_2_ (20 vol%) + He (balance)	130	100	7.5 × 10^−9^	100 000 mL h^−1^ g^−1^	88	212	40	179	2.2	8.95	5.3	5
5	Ag/TiO_2_ (anatase)	8	60	35.3	Calcination at 450 °C for 3 h	FA + O_2_ (20 vol%) + He (balance)	130	100	7.5 × 10^−9^	100 000 mL h^−1^ g^−1^	91	110	30	135	1.7	3.82	10.9	5
6	Ag/PbO	0.02	150	2.1	Thermal treatment at 300 °C using N_2_ for 3 h (50 mL min^−1^)	FA + air (balance)	100	50	2.9 × 10^−9^	9436 h^−1^	103	130	37	26	128	12.4	128	This study
7	Ag/PbO	0.2	150	1.6	Thermal treatment at 300 °C using N_2_ for 3 h (50 mL min^−1^)	FA + air (balance)	100	50	2.9 × 10^−9^	9436 h^−1^	52	75	100	69	34	43.1	34	This study
8	Ag/PbO	0.9	150	1.5	Thermal treatment at 300 °C using N_2_ for 3 h (50 mL min^−1^)	FA + air (balance)	100	50	2.9 × 10^−9^	9436 h^−1^	18^b^	47	100	69	7.7	46	7.7	This study
9	Ag/PbO	0.9	150	1.5	Reduction at 300 °C using H_2_ (10%) + N_2_ mixture for 3 h (50 mL min^−1^)	FA + air (balance)	100	50	2.9 × 10^−9^	9436 h^−1^	93	96	72	50	5.5	33.3	5.5	This study

a Calculated at 80 °C.

b Value estimated by extrapolation, as conversion at the lowest measured temperature (30 °C) already exceeded 50%.

*r* was also compared after normalization to the Ag content for each study to reduce the bias associated with different noble metal loadings. In this context, all the catalysts examined in this study exhibited superior performance compared to previously reported catalysts for FA oxidation in the air under DLT conditions (Table 1). In terms of normalized *r* (128 mmol g^−1^ h^−1^), 0.02%-Ag/PbO was the best performer (Table 1). The efficiency of the other catalysts evaluated in this study followed the descending trend based on the normalized *r* (mmol g^−1^ h^−1^): 0.2%-Ag/PbO (34) > 0.9%-Ag/PbO (7.7) > 0.9%-Ag/PbO-R (5.5) (Table 1). The next-best performer was Ag–K/Al_2_O_3_ (normalized *r* of 4.9 mmol g^−1^ h^−1^) (Table 1). The lowest normalized *r* (1.7 mmol g^−1^ h^−1^) was seen for Ag/TiO_2_ (anatase) (Table 1). Enhanced performance of the Ag/PbO catalysts can be ascribed to the reactive adsorption of FA molecules on the PbO support and the favorable oxidation state (^*δ*+^) of Ag sites.

The mass-based rates can be skewed by large differences in catalyst surface area. To evaluate the intrinsic activity of the catalyst surface, we also compared the BET-normalized rate (*r*_BET_), which accounts for these differences. This analysis revealed a dramatically different performance landscape. Our Ag/PbO catalysts, despite having very low BET surface areas (1.5–2.1 m^2^ g^−1^), demonstrated exceptionally high intrinsic activities. The 0.9%-Ag/PbO catalyst achieved the highest *r*_BET_ of 46 µmol m^−2^ h^−1^, followed closely by the 0.2%-Ag/PbO catalyst (43.1 µmol m^−2^ h^−1^) (Table 1). These values are nearly five times higher than those of the best-performing literature catalysts on this metric, Ag/TiO_2_ (anatase) (9.12 µmol m^−2^ h^−1^) (Table 1). In contrast, the Ag/Al_2_O_3_ catalyst, which has a very high surface area (154 m^2^ g^−1^), showed the lowest intrinsic activity (1.6 µmol m^−2^ h^−1^). This comparison highlights two key points: (i) the Ag/PbO catalysts possess a surface that is intrinsically far more active for FA oxidation than conventional TiO_2_- or Al_2_O_3_-supported Ag catalysts, reinforcing the conclusion of a unique and highly efficient Ag–PbO interface. (ii) The overall mass-based performance of the Ag/PbO system is currently limited by its low surface area, suggesting that future improvements could be realized by synthesizing high-surface-area PbO-based materials. In terms of Ag-mass-normalized rate (*r* normalized to Ag content), the 0.02%-Ag/PbO was the most efficient catalyst (Table 1), which is consistent with the well-known principle that lower metal loadings often lead to better dispersion and higher per-atom efficiency.

To further quantify the per-atom efficiency of the catalysts, we estimated the turnover frequency (TOF) by dividing the Ag-mass-normalized *r* by the Ag dispersion. For literature catalysts with reported particle sizes, dispersion was derived from the volume-area mean diameter. Since experimental dispersion data were not available for our Ag/PbO catalysts, a conservative assumption of 100% dispersion was made. This approach provides a lower-bound estimate of the TOF, as any realistic dispersion below 100% would result in an even higher calculated value. As shown in Table 1, this analysis highlights the remarkable performance of our system, with the 0.02%-Ag/PbO catalyst exhibiting an estimated TOF of 128 mmol mol^−1^ s^−1^, a value significantly greater than the literature benchmarks. This exceptionally high TOF, even with a conservative assumption, reinforces that the enhanced performance of the Ag/PbO catalysts is driven by the high intrinsic activity of the active sites, which can be ascribed to the reactive adsorption of FA molecules on the PbO support and the favorable oxidation state of the Ag species.

## 5. Conclusions

In the present research, Ag/PbO catalysts were utilized for FA oxidation in air under DLT (including RT) conditions. Higher Ag loading (0.02 to 0.9 wt%) improved *X*_FA_, as the increased number of Ag sites facilitated O_2_ activation on the catalyst surface. The reduction pre-treatment decreased *X*_FA_ since Ag^0^ requires higher temperatures for activating O_2_ than the Ag^*δ*+^ species. The RT *X*_FA_ for the tested catalysts decreased as follows: 0.9%-Ag/PbO (83%) > 0.9%-Ag/PbO-R (56%) > 0.2%-Ag/PbO (39%) > 0.02%-Ag/PbO (35%). Under humid RT conditions, 0.9%-Ag/PbO exhibited 100% *X*_FA_. Nevertheless, the low *Y*_CO_2__ (≤30%) at this temperature confirms that the primary fate of FA is surface sequestration as intermediates rather than complete oxidation. True mineralization (100% *Y*_CO_2__) was only realized at 80 °C, highlighting a critical temperature threshold for catalytic destruction. 0.02%-Ag/PbO was identified as the most efficient supported Ag catalyst for FA oxidation under DLT conditions, based on normalized *r*, when compared to similar catalysts. DFT simulations and experimental characterization demonstrate that the Ag/PbO surface, particularly at interfacial regions near Ag clusters, efficiently activates O_2_ and H_2_O, generating abundant *O species that drive FA oxidation. In comparison to Ag/PbO-R, Ag/PbO maintains a higher concentration of *O, as these oxygen species tend to accumulate at interfacial sites rather than being depleted to restore the lattice structure. Furthermore, moisture enhances the reaction by promoting *OH formation, which facilitates FA hydration into the key intermediate *CH_2_(OH)_2_, leading to a more efficient oxidation process governed by nearby *O species. This study presents a detailed mechanistic investigation of a highly active Ag/PbO catalyst. We have demonstrated a moisture-promoted pathway where FA hydration to a key CH_2_(OH)_2_ intermediate is crucial for the efficient, low-temperature oxidation process. These findings provide a new framework for the rational design of future catalysts by highlighting the powerful synergistic effects possible at the metal–support interface in humid conditions.

## Conflicts of interest

There are no conflicts to declare.

## Supplementary Material

RA-OLF-D6RA01138A-s001

## Data Availability

The data supporting the findings of this study are available within the article and its supplementary information (SI). Additional data can be provided by the corresponding author upon reasonable request. Supplementary information is available. See DOI: https://doi.org/10.1039/d6ra01138a.
