# Supplementary material for: The synergistic role of moisture and the silver–lead(ii) oxide interface in room-temperature catalytic oxidation of gaseous formaldehyde
Source: RSC Adv. 2026 Aug 13;16(40):47320–37. doi: 10.1039/d6ra01138a (PMC13471811; doi:10.1039/d6ra01138a)
Supplement: Supplementary file 1 [file RA-016-D6RA01138A-s001.pdf]

Supplementary Information (SI) for RSC Advances.
This journal is © The Royal Society of Chemistry 2026

Supplementary Information

The synergistic role of moisture and the silver-lead(II) oxide interface in room-temperature catalytic oxidation of gaseous formaldehyde

Kumar Vikrant [a, b, c], Ki-Hyun Kim [c, *], Philippe M. Heynderickx [d, e], Changho Yeon [f], Chan-Woo Lee [f]

[a] College of Engineering and Computer Science, VinUniversity, Vinhomes Ocean Park, Gia Lâm, Hanoi 100000, Vietnam

[b] Center for Materials Innovation and Technology, VinUniversity, Vinhomes Ocean Park, Gia Lâm, Hanoi 100000, Vietnam

[c] Department of Civil and Environmental Engineering, Hanyang University, 222 Wangsimni-Ro, Seoul 04763, Republic of Korea

[d] Center for Green Chemistry and Environmental Biotechnology (GREAT), Ghent University Global Campus, 119-5 Songdo Munhwa-ro, Yeonsu-gu, Incheon 406-840, Republic of Korea

[e] Department of Green Chemistry and Technology, Faculty of Bioscience Engineering, Ghent University, Coupure Links 653, B-9000 Ghent, Belgium

[f] Department of Energy and Materials Engineering, Dongguk University, 30 Pildong-ro 1-gil, Jung-gu, Seoul 04620, Republic of Korea

***Correspondence: kkim61@hanyang.ac.kr (K.-H. Kim)**

Table of Contents

Characterization instrumentation………………………………………………………………………….…S3

Temperature-programmed reduction ……………………………………………………………………..…S5

Experimental…………………………………………………………………………………………….…S5

Model description: Gaussian deconvolution and peak analysis…………………………………………S7

Reduction kinetics determination…………………………………………………………………………....S8

Standard deviation calculation…..………………………………………………………………………......S9

External mass transfer limitation……………………………………………………………….………......S11

Internal mass transfer limitation…………………………………………………………………….……S14

External heat transfer limitation…………………………………………………………………….……S15

Internal heat transfer limitation……………………………………………………………….…………..S17

Radial heat transfer limitation………………………………………………………………………….……S18

Calculation of turnover frequency……………………………………………………………………….....S19

Tables and Figures……………………………………………………………………………………….…...S20

References…………………………………………………………………………………………….……..S53

S1. Characterization instrumentation

The crystal structures of the test materials were analyzed using powder X-ray diffraction (PXRD) patterns (D8 ADVANCE (Bruker Corp., Billerica, MA, USA)). The PXRD data were obtained in the 15-80° range using a scan speed and step size of 4° min^{-1} and 0.02°, respectively. The simulated α-lead(II) oxide (PbO) PXRD pattern was drawn using a suitable crystallographic information file (obtained from the crystallography open database) and Visualization for Electronic and Structural Analysis (*VESTA*) software [1]. The powder samples were placed on an alumina pan for conducting the thermogravimetric analysis (TGA (SDT Q600 Auto-DSCQ20 system, TA Instruments, Inc., New Castle, DE, USA)). The samples were heated from room temperature (RT) to ~ 800°C (10°C min^{-1}) under nitrogen (N_2) flow (100 mL min^{-1}). The N_2 adsorption-desorption isotherms of the tested materials were acquired at 77 K (Micromeritics ASAP 2010, Micromeritics Instrument Corp., Norcross, GA, USA) for assessing their Brunauer–Emmett–Teller surface areas and pore size distributions. The actual silver (Ag) content in the tested materials was assessed using ICP-OES (iCAP™ 7000 Series, Thermo Fisher Scientific, Waltham, MA, USA). The morphologies of the test materials and their surface elemental distributions were assessed using a field emission scanning electron microscope (SEM) equipped with an energy-dispersive X-ray spectroscopy (EDX) probe (Verios™ 5 XHR, Thermo Fisher Scientific, Waltham, MA, USA). The high-resolution transmission electron microscope (TEM) images were obtained using JEM-2100F (JEOL Ltd., Tokyo, Japan). Particle size distribution based on SEM images was measured using ImageJ software [2]. The Ag nanoparticle (NP) size distribution based on the TEM images was also measured using the ImageJ software. A Gaussian function was utilized to fit the particle size distribution data and to measure the average value. Fourier-transform infrared spectroscopy (FTIR; Nicolet™ iS™ 50, Thermo Fisher Scientific, Waltham, MA, USA) was used to assess the surface functional groups of the test materials

in the 400-4000 cm^{-1} range. FTIR spectra were obtained using the attenuated total reflectance (ATR) sampling technique. X-ray photoelectron spectroscopy (XPS; K-alpha™ system, Thermo Fisher Scientific, Waltham, MA, USA) was used to assess the surface chemistry of the test materials. All acquired XPS data were charge-corrected to adventitious carbon (285 eV) [3].

S2. Temperature-programmed reduction

S2.1. Experimental

Hydrogen (H_2)-temperature-programmed reduction (TPR) experiments were performed using AutoChem™ II (Micromeritics Instrument Corp., Norcross, GA, USA) at heating rates of 1.6, 4, and 10 K min^{-1} up to 873.2 K under a flow of H_2 (10% in argon (Ar)) with a total volumetric flow rate of 50 mL min^{-1} (2.04 mmol min^{-1}), using 60.4 ± 0.3 mg of the support (PbO) or the catalyst (Ag/PbO or Ag/PbO-R). During analysis, the effluent was subcooled using isopropyl alcohol at -80°C so that the formed water (H_2O) did not distort the thermal conductivity detector (TCD) signal, and only the H_2 consumption was measured. Prior to every TPR experiment, the loaded sample was maximally oxidized by a flow (total molar flow rate of 50 mL min^{-1}) of molecular oxygen (O_2 (5% in Ar)). The temperature was raised using a heating rate of 10 K min^{-1} from RT to 383.2 K to remove possible adsorbates (e.g., H_2O and CO_2). The sample was cooled down to 303.2 K under a 50 mL min^{-1} Ar flow to remove weakly adsorbed oxygen species. The criterion from Malet and Caballero was evaluated to secure the resolution in multistep reduction processes in the TPR data (Equation S1) [4]. P, β, S_0, and $F{\cdot}C_0$ represent the upper limit for the criterion (normally 20 K), the heating rate (K min^{-1}), the number of reducible species (mol), and the H_2 fed to the reactor (mol min^{-1}).

$$P > \frac{\beta S_0}{FC_0} \qquad \text{(S1)}$$

From the experimental TPR data and calibration for H_2, the number of oxygen atoms taken out of PbO, 0.9%-Ag/PbO, and 0.9%-Ag/PbO-R were 181.9 ± 11, 181.8 ± 14.3, and 211.1 ± 3.6 μmol_{H2}, respectively. According to the stoichiometric surface reduction reaction $H_2 + O^* \rightarrow H_2O + *$, these

values correspond to an available oxygen concentration of 3.01 ± 0.19, 3 ± 0.23, and 3.51 ± 0.06 mol_{O*} kg_{cat}^{-1}, respectively. Using the values P = 20 K, β = 10 K min^{-1} (highest value), S_0 = 211 μmol, and $F \cdot C_0$ = 2.04 mmol min^{-1} × 0.10 = 200 μmol min^{-1}, the right-hand side of Equation S1 equals 10.6 K at the most. Hence, the obtained TPR data were considered not to lose resolution for further modeling.

S2.2. Model description: Gaussian deconvolution and peak analysis

The experimental H_2-TPR data were modeled using the sum of N Gaussian distribution functions (Equation S2). x and y represent the desorption/reduction temperature (K) and the TCD output signal, respectively.

$$y(x)=\frac{1}{\sqrt{2\pi}}\cdot\sum_{i=1}^{N}\frac{a_i}{\sigma_i}\cdot\exp\left(-\frac{(x-\mu_i)^2}{2\sigma_i^2}\right) \tag{S2}$$

a represents the scaling factor for each Gaussian profile, μ the average (where the peak temperature, T_{max}, was located), and σ^2 the variance. The model parameters (a, μ, and σ)$_{i=1...N}$ were obtained by minimizing the objective function S for n data points (Equation S3).

$$S=\sum_{i=1}^{n}\left(y_{i,\exp}-y_{i,calc}\right)^2 \xrightarrow[(a_i,\mu_i,\sigma_i)_{i=1...N}]{} \min \tag{S3}$$

$\frac{a_i}{\sum_{i=j}^{N} a_j}$ represents the fraction of surface oxygen (O_S) atoms in each peak. The total oxygen consumption was calculated from the total profile area.

S2.3. Reduction kinetics determination

Kinetic parameters of the TPR process for peak (i), the pre-exponential factor (A_i), and activation energy (E_i) were supplied by the Redhead equation [5] (Equation S4). T_{max} represents peak maximum temperature. The relation originates from a power law rate expression for reduction, with the initial condition for a fraction of oxidized sites $\theta_{O^*} = 1$ (fully oxidized in the pretreatment) at T = T_0 (= 303.15 K) (Equation S5). For first-order dependency on the fractional oxygen coverage, n equals unity, i.e., an irreversible catalyst reduction reaction (H_2 + O* → H_2O + *), which can be considered a reactive desorption step.

$$\frac{E_i}{RT_{\max,i}^2} = \frac{A_i}{\beta}\exp\left(-\frac{E_i}{RT_{\max,i}}\right) \tag{S4}$$

$$\frac{d\theta_{O^*}}{dT} = A\cdot\exp\left(-\frac{E}{RT}\right)\cdot\frac{\theta_{O^*}^n}{\beta} \tag{S5}$$

Linearization results in Equation S6. Plotting the left-hand side versus the inverse of the maximal temperature per peak yields a linear relationship from which the activation energy and the pre-exponential factor can be determined from the couples (T_{max}, β)$_{©}$, with i = 1…N (N = 3) [6-7].

$$\ln\frac{T_{\max,i}^2}{\beta} = \ln\left(\frac{E_i}{R\,A_i}\right) + \frac{E_i}{RT_{\max,i}} \tag{S6}$$

S2.4. Standard deviation calculation

The standard deviation for the parameters in the Gaussian function was calculated using the covariance matrix [8-9]. Under the assumption that experimental errors were normally distributed with zero mean and constant variance and were statistically independent, the random variable $\frac{|b_i - \beta_i|}{s(b_i)}$ $\sim t(n-p)$ with $s(b_i) = \sqrt{\underline{\underline{\hat{V}}}(b)_{i,i}}$ was distributed like t with n-p degrees of freedom. The above was used to test hypothesis H_0: $\beta_i = \bar{b}_i$, provided that all other parameters were kept constant at their optimal estimate. The value for $\underline{\underline{\hat{V}}}(b)_{i,i}$, the diagonal elements of $\underline{\underline{\hat{V}}}(b)$, was obtained using Equations S7, S8, and S9, where $\underline{\underline{J}}$ was evaluated at the converged b values and $\hat{F}$ were the output data j = 1…n (*F* represents the experimental data). The partial derivatives were calculated using Equations S10, S11, and S12. y_i was the ith peak in the total signal.

$$\underline{\underline{\hat{V}}}(b) = \left(\underline{\underline{J}}^T \underline{\underline{J}}\right)^{-1} s^2 \tag{S7}$$

$$\left(\underline{\underline{J}}\right)_{j,i} = \left(\frac{\partial F_j}{\partial \beta_i}\right)_{\underline{\beta}=\underline{b}} \tag{S8}$$

$$s^2 = \frac{1}{n-p} \cdot \sum_{j=1}^{n} \left(F_j - \hat{F}_j\right)^2 \tag{S9}$$

$$\frac{\partial y}{\partial a_i} = \frac{y_i}{a_i} \tag{S10}$$

$$\frac{\partial y_i}{\partial \mu_i} = \frac{x - \mu_i}{\sigma_i^2} \cdot y_i \tag{S11}$$

$$\frac{\partial y_i}{\partial \sigma_i} = -\frac{y_i}{\sigma_i} + \frac{\left(x - \mu_i\right)^2}{\sigma_i^3} \cdot y_i \tag{S12}$$

S3. External mass transfer limitation

For a particle Reynolds number (Re) between 0.1 and 100, the Sherwood number (Sh) in a packed bed is related to the Re and the Schmidt number (Sc) using Equation S13. The Sh is defined in Equation S14. The mass transfer coefficient, particle diameter, and the bulk diffusion coefficient of FA in air are represented by k_f, d_p, and $D_{FA,air}$, respectively. A d_p of 40 µm was used for 0.9%-Ag/PbO. $D_{FA,air}$ was calculated as 3.3E-05 m^2 s^{-1} using the Fuller equation at the reaction temperature (T (155°C)) and pressure (P (1 atm)) (Equation S15). M_{FA} (30.031 g mol^{-1}), M_{air} (28.96 g mol^{-1}), $\Sigma\nu_{FA}$, and $\Sigma\nu_{air}$ represent the FA molecular weight, air molecular weight, the sum of the diffusion volume for FA, and the sum of the diffusion volume for air, respectively. The Re of 0.103 was calculated using Equation S16. The superficial velocity (μ_0) was calculated as 0.29 m s^{-1} using Equation S17. $F_{v,0}$, d_t, and ε_b represent the total inlet volumetric flow rate (8E-07 m^3 s^{-1}), reactor inner tube diameter (0.3 cm), and bed porosity (0.4 m^3 m^{-3}), respectively. The fluid density (ρ_f) was calculated as 0.83 kg m^{-3} using Equation S18. M_{av} (29 g mol^{-1}) and R (0.0821 L atm mol^{-1} K^{-1}) represent the average molar mass and the universal gas constant, respectively.

The fluid viscosity (μ_f) was calculated as 9.5E-05 Ns m^{-2} using Equations S19-S21. The A, B, and C coefficients for O_2 and N_2 are listed in [10]. The A, B, and C coefficients for FA were -4.0407, 0.368, and -5.9E-05, respectively. Also, x and m represent the mole fraction and molar mass, respectively. The Sc of 3.53 was calculated using Equation S22. The Sh and k_f were then calculated as 2.43 and 1.973 m s^{-1} using Equations S13 and S14, respectively. The specific external surface area (a_v) was calculated as 150,000 m^{-1} ($6/d_p$). The bulk FA concentration ($C_{FA,b}$) was calculated as 2.8E-03 mol m^{-3} (FA inlet mole fraction ($x_{FA,0}$ = 9.9E-05)) using Equation S23. The observed volumetric reaction rate ($r_{v,B}^{obs}$) was calculated as 1.5E-02 mol m^{-3} s^{-1} under specific reaction conditions (FA inlet molar flow rate ($F_{FA,0}$) = 2.4E-09 mol s^{-1}, catalyst mass (m_{cat}) = 0.15

g, and FA conversion (X_B) = 1). Note that ρ_p indicates the bulk particle density. Using the above-calculated values, the left-hand-side (LHS) value (1.8E-05) of the external mass transfer criterion was less than the right-hand-side (RHS) value (0.05), satisfying the criterion (the reaction order (n) was assumed as 1) (Equation S24). Hence, the external mass transfer gradients do not significantly occur for the given experimental conditions.

$$Sh = 2 + 1.1 \cdot Re^{0.6} Sc^{0.33} \quad \text{(S13)}$$

$$Sh = \frac{k_f d_p}{D_{FA,air}} \quad \text{(S14)}$$

$$D_{FA,air} = \frac{10^{-3} T^{1.75} \left(\frac{1}{M_{FA}} + \frac{1}{M_{air}}\right)^{\frac{1}{2}}}{P\left((\Sigma \nu_{FA})^{\frac{1}{3}} + (\Sigma \nu_{air})^{\frac{1}{3}}\right)^2} \quad \text{(S15)}$$

$$Re = \frac{\mu_0 d_p \rho_f}{\mu_f} \quad \text{(S16)}$$

$$\mu_0 = \frac{F_{v,0}}{\frac{\pi d_t^2}{4} \cdot \varepsilon_b} \quad \text{(S17)}$$

$$\rho_f = \frac{M_{av} P}{RT} \quad \text{(S18)}$$

$$\mu_i = A_i + B_i \cdot T + C_i \cdot T^2 \quad \text{(S19)}$$

$$u_f = \sum_{i=1}^{n} \frac{x_i \mu_i}{\sum_{j=1}^{n} x_j \Phi_{ij}} \quad \text{(S20)}$$

$$\Phi_{ij} = \frac{\left(1 + \sqrt{\frac{\mu_i}{\mu_j}} \cdot \left(\frac{m_j}{m_i}\right)^{\frac{1}{4}}\right)^2}{\sqrt{8} \cdot \sqrt{1 + \frac{m_i}{m_j}}} \quad \text{(S21)}$$

$$Sc = \frac{\mu_f}{\rho_f D_{FA,air}} \quad \text{(S22)}$$

$$C_{FA,b} = \frac{P \cdot x_{FA,0}}{RT} \tag{S23}$$

$$\frac{r_{v,FA}^{obs}}{k_f a_v C_{FA,b}} < \frac{0.05}{n} \tag{S24}$$

S4. Internal mass transfer limitation

FA concentration on the catalyst particle surface ($C_{FA,s}$) was assumed to be equal to $C_{FA,b}$ as the external mass transfer limitations were insignificant under the given experimental condition. The Knudsen diffusivity for benzene ($D_{FA,K}$) was calculated as 1.7E-08 m^2 s^{-1} using Equation S25. The average pore radius ($r_{p,av}$) of 0.9%-Ag/PbO was taken as 3 nm. The effective diffusivity for FA ($D_{FA,eff}$) was calculated as 3.5E-09 m^2 s^{-1} using Equation S26. The particle porosity (ε_p) and tortuosity (τ_p) were taken as 0.6 m^3 m^{-3} and 3 m m^{-1}, respectively. Using the above-calculated values, the LHS value (0.067) of the internal mass transfer criterion (Weisz-Prater criterion) was less than the RHS value (0.083) needed to satisfy it (Equation S27). Hence, internal mass transfer gradients do not occur significantly for the given experimental conditions.

$$D_{FA,K} = \frac{2}{3} \cdot r_{p,av} \cdot \sqrt{\frac{8RT}{\pi M_{FA}}} \tag{S25}$$

$$D_{FA,eff} = \frac{\varepsilon_p}{\tau_p} \cdot \left(\frac{1}{D_{FA,air}} + \frac{1}{D_{FA,K}}\right)^{-1} \tag{S26}$$

$$\frac{r_{v,FA}^{obs}}{D_{FA,eff} C_{FA,s}} \cdot \left(\frac{d_p}{6}\right)^2 < \frac{1}{12n} \tag{S27}$$

S5. External heat transfer limitation

The reaction enthalpy ($\Delta_r H$) for FA oxidation ($CH_2O + O_2 \rightarrow CO_2 + H_2O$) at 155°C and 1 atm was calculated as -116.11 kJ mol^{-1} using the enthalpy of formation ($\Delta_f H$) values for the reactants and products (Equation S28). The A, B, and C coefficients for calculating the $\Delta_f H$ values for CO_2 and H_2O are listed in [10]. The $\Delta_f H$ value for O_2 at 155°C was calculated as 3.9 kJ mol^{-1} by integrating its heat capacity (c_p) from 298.15 to 428.15 K (Equation S29). The $\Delta_f H$ for FA was -120 kJ mol^{-1}. The A, B, C, D, and E coefficients for calculating the c_p values for O_2 and N_2 are listed in [10]. c_p for FA was 119.2 J mol^{-1} K^{-1}. The fluid c_p ($c_{p,f}$) was calculated as 29.6 J mol^{-1} K^{-1} using Equation S30. The fluid thermal conductivity (λ_f) was calculated as 0.134 W m^{-1} K^{-1} using Equations S31-S33. The A, B, and C coefficients for calculating the λ values for O_2 and N_2 are listed in [10]. The λ for FA was 0.026 W m^{-1} K^{-1}. The Prandtl number (Pr) was calculated to be 7.2E-04 using Equation S34. The Nusselt number (Nu) was calculated to be 2.03 using Equation S35. Note that Equation S35 is valid for a Re between 0.1 and 100. The bulk-catalyst heat transfer coefficient (α) was calculated as 6.77 kW m^{-2} K^{-1} using Equation S36. The activation energy (E_a) for the FA oxidation reaction was assumed as 100 kJ mol^{-1}. Using the above-calculated values, the LHS value (4.6E-04) of the external heat transfer criterion was less than the RHS value (4.57) to satisfactorily satisfy it (Equation S37). Hence, the external heat transfer gradients do not significantly occur for the given experimental conditions.

$$\Delta_f H_i = A_i + B_i \cdot T + C_i \cdot T^2 \tag{S28}$$

$$c_{p,i} = A_i + B_i \cdot T + C_i \cdot T^2 + D_i \cdot T^3 + E_i \cdot T^4 \tag{S29}$$

$$c_{p,f} = \sum_{i=1}^{n} x_i c_{p,i} \tag{S30}$$

$$\lambda_i = A_i + B_i \cdot T + C_i \cdot T^2 \tag{S31}$$

$$\lambda_f = \sum_{i=1}^{n} \frac{x_i \lambda_i}{\sum_{j=1}^{n} x_j \Phi_{ij}} \tag{S32}$$

$$\Phi_{ij} = \frac{\left(1 + \sqrt{\frac{\lambda_i}{\lambda_j}} \cdot \left(\frac{m_j}{m_i}\right)^{\frac{1}{4}}\right)^2}{\sqrt{8} \cdot \sqrt{1 + \frac{m_i}{m_j}}} \tag{S33}$$

$$\mathrm{Pr} = \frac{c_{p,f} \mu_f}{\lambda_f M_{av}} \tag{S34}$$

$$Nu = 2 + 1.1 \cdot Re^{0.6} Pr^{0.33} \tag{S35}$$

$$\alpha = \frac{Nu \lambda_f}{d_p} \tag{S36}$$

$$\frac{r_{v,FA}^{obs} |\Delta_r H| d_p}{\alpha} < 0.3 \cdot \frac{RT^2}{E_a} \tag{S37}$$

S6. Internal heat transfer limitation

The catalyst particle thermal conductivity (λ_p) was calculated as 0.214 W m^{-1} K^{-1} using Equation S38. Note that the catalyst particle solid phase thermal conductivity ($\lambda_{p,s}$) was assumed to be 2.2 W m^{-1} K^{-1} [11]. Using the above-calculated values, the LHS value (5.8E-05) of the internal heat transfer criterion was less than the RHS value (45.7), satisfying the criterion (Equation S39). Hence, internal heat transfer gradients do not occur significantly for the given experimental conditions.

$$\frac{1}{\lambda_p} = \frac{1-\varepsilon_p}{\lambda_{p,s}} + \frac{\varepsilon_p}{\lambda_f} \tag{S38}$$

$$\frac{r_{v,FA}^{obs}|\Delta_r H| d_p^2}{\lambda_p} < 3 \cdot \frac{RT^2}{E_a} \tag{S39}$$

S7. Radial heat transfer limitation

The volumetric fraction of inert material in the catalyst bed (b) was calculated as 0.71 using Equation S40. The diluent mass (m_{dil}) and density (ρ_{dil}) values were 600 mg and 1,540 mg cm^{-3}, respectively. The volumetric average particle thermal conductivity in the bed (λ_s) was calculated as 0.537 W m^{-1} K^{-1} using Equation S41. The value for the diluent thermal conductivity (λ_{dil}) was assumed as 1.4 W m^{-1} K^{-1}. The static contribution ($\lambda_{er,0}$) to the effective radial bed thermal conductivity (λ_{er}) was calculated as 0.392 W m^{-1} K^{-1} using Equation S42. For the radial heat transfer evaluation, the Re was calculated as 7.71 using Equation S43. λ_{er} was calculated as 0.392 W m^{-1} K^{-1} using Equation S44. Using the above-calculated values, the LHS value (0.031) of the radial heat transfer criterion was less than the RHS value (24.4), satisfying the criterion (Equation S45). Hence, radial heat transfer gradients do not occur significantly for the given experimental conditions.

$$b = \frac{\frac{m_{dil}}{\rho_{dil}}}{\frac{m_{cat}}{\rho_p} + \frac{m_{dil}}{\rho_{dil}}} \tag{S40}$$

$$\frac{1}{\lambda_s} = \frac{1-b}{\lambda_p} + \frac{b}{\lambda_{dil}} \tag{S41}$$

$$\lambda_{er,0} = \varepsilon_b \cdot \lambda_f + (1 - \varepsilon_b) \cdot \lambda_s \cdot \frac{2.44\sqrt{\lambda_f \lambda_s}}{\lambda_f + \lambda_s} \tag{S42}$$

$$Re = \frac{\mu_0 d_t \rho_f}{\mu_f} \tag{S43}$$

$$\lambda_{er} = \lambda_{er,0} + \frac{0.123 \cdot Re \cdot Pr . \lambda_f}{1 + 46\left(\frac{d_p}{d_t}\right)^2} \tag{S44}$$

$$\frac{r_{v,FA}^{obs} |\Delta_r H| (1-\varepsilon_b)(1-b) d_t^2}{\lambda_{er}} < 1.6 \cdot \frac{RT^2}{E_a} \tag{S45}$$

S8. Calculation of turnover frequency

The turnover frequency (TOF) was estimated to compare the per-atom efficiency of the catalysts. For literature catalysts where the average particle diameter was reported, the Ag dispersion (D_{Ag}) was first calculated. The TOF was then determined by dividing the Ag-mass-normalized reaction rate by the dispersion. The dispersion is related to the volume-area mean diameter (d_{VA}) of the spherical particles. The volume-area mean diameter is defined in Equation S46.

$$d_{VA} = \frac{\sum n_i d_i^3}{\sum n_i d_i^2} \tag{S46}$$

Where n_i is the number of particles with diameter d_i. The relationship between dispersion (D_{Ag}), the volume-area mean diameter (nm), the surface area occupied by an Ag atom (a_m = 8.75 Å^2), and the volume occupied by an Ag atom in the bulk metal (v_m = 17.06 Å^3) is given by Equation S47 [12].

$$D_{Ag} = \frac{6}{d_{VA}} \cdot \frac{v_m}{a_m} \tag{S47}$$

For Ag, this simplifies to Equation S48. For the catalysts in this study, experimental dispersion data were not available. Therefore, a conservative assumption of 100% dispersion was made, which provides a lower-bound estimate for the TOF.

$$D_{Ag} = \frac{1.17}{d_{VA}} \tag{S48}$$

S9. Tables and Figures

Table S1. Physical properties of the tested materials.

Order	Sample	BET surface area	Pore volume[a]	Actual content (wt.%)[b]
		**$m^2 g^{-1}$**	**$cm^3 g^{-1}$**	**Ag**
1	PbO	2.4 ± 0.02	0.003	-
2	0.02%-Ag/PbO	2.1 ± 0.04	0.002	0.02
3	0.2%-Ag/PbO	1.6 ± 0.01	0.002	0.21
4	0.9%-Ag/PbO	1.5 ± 0.04	0.002	0.9

[a] BJH adsorption cumulative volume of pores between 0.1 nm and 300 nm in diameter

[b] Determined by ICP-OES

Table S2. O_S concentration (under TPR reductive conditions) for the materials used and values for RSSQ per heating rate with average error (% error in brackets).

Material	n_{O*} (μmol_{O*})	C_{O*} ($mmol_{O*}\ kg_{cat}^{-1}$)	β ($K\ min^{-1}$)	RSSQ (10^{-4} a.u.^{2})	Average error (%.)
PbO	181.9 ± 11.0	3.01 ± 0.19	1.6	1.61	2.25
			4	1.80	1.10
			10	1.83	0.54
0.9%Ag/PbO	181.8 ± 14.3	3.00 ± 0.23	1.6	0.25	0.89
			4	1.03	0.85
			10	1.72	0.54
0.9%Ag/PbO-R	211.1 ± 3.6	3.51 ± 0.06	1.6	2.56	1.76
			4	3.76	0.99
			10	5.24	0.64

Table S3. Kinetic parameters for the H_2-TPR data of the PbO support.

Peak	β (K min^{-1})	Signal (%)	T_{max} (K)	A_i (s^{-1})	E_i (kJ mol^{-1})
1	1.6	25.5	335.6 ± 3.2	(6.97 ± 0.35) 10^{+13}	105.1 ± 2.7
	4	29.9	343.2 ± 0.5		
	10	24.4	351.8 ± 0.4		
2	1.6	52.6	379.8 ± 5.1	(2.00 ± 0.50) 10^{+3}	45.7 ± 0.8
	4	51.8	401.4 ± 2.5		
	10	53.2	426.9 ± 0.9		
3	1.6	21.9	404.4 ± 1.7	(3.90 ± 0.16) 10^{+2}	36.6 ± 0.6
	4	18.3	434.3 ± 1.2		
	10	22.6	470.4 ± 1.3		

Table S4. Kinetic parameters for the H_2-TPR data of the 0.9%-Ag/PbO catalyst.

Peak	β (K min^{-1})	Signal (%)	T_{max} (K)	A_i (s^{-1})	E_i (kJ mol^{-1})
1	1.6	34.6	344.1 ± 2.5	(7.36 ± 0.12) 10^{+7}	69.8 ± 0.4
	4	33.2	356.5 ± 1.0		
	10	34.7	369.7 ± 0.7		
2	1.6	44.7	386.3 ± 1.3	(5.08 ± 0.24) 10^{+2}	42.5 ± 0.4
	4	44.9	411.2 ± 1.4		
	10	44.8	438.6 ± 1.3		
3	1.6	20.6	414.7 ± 0.5	(1.22 ± 0.01) 10^{+2}	41.3 ± 0.1
	4	21.9	443.8 ± 0.4		
	10	20.5	476.6 ± 1.3		

Table S5. Kinetic parameters for the H_2-TPR data of the 0.9%-Ag/PbO-R catalyst.

**Peak**	**β (K min^{-1})**	**Signal (%)**	**T_{max} (K)**	**A_i (s^{-1})**	**E_i (kJ mol^{-1})**
1	1.6	50.3	386.4 ± 4.7	(9.82 ± 0.39) 10^{+4}	58.4 ± 0.3
	4	49.9	404.9 ± 3.2		
	10	50.8	424.8 ± 1.3		
2	1.6	43.1	419.8 ± 0.6	(4.97 ± 0.64) 10^{+4}	61.5 ± 0.9
	4	43.4	440.8 ± 1.3		
	10	43.8	462.8 ± 0.4		
3	1.6	6.6	429.3 ± 1.0	(1.54 ± 0.04) 10^{+1}	36.1 ± 0.5
	4	6.7	463.6 ± 0.5		
	10	5.4	504.7 ± 0.8		

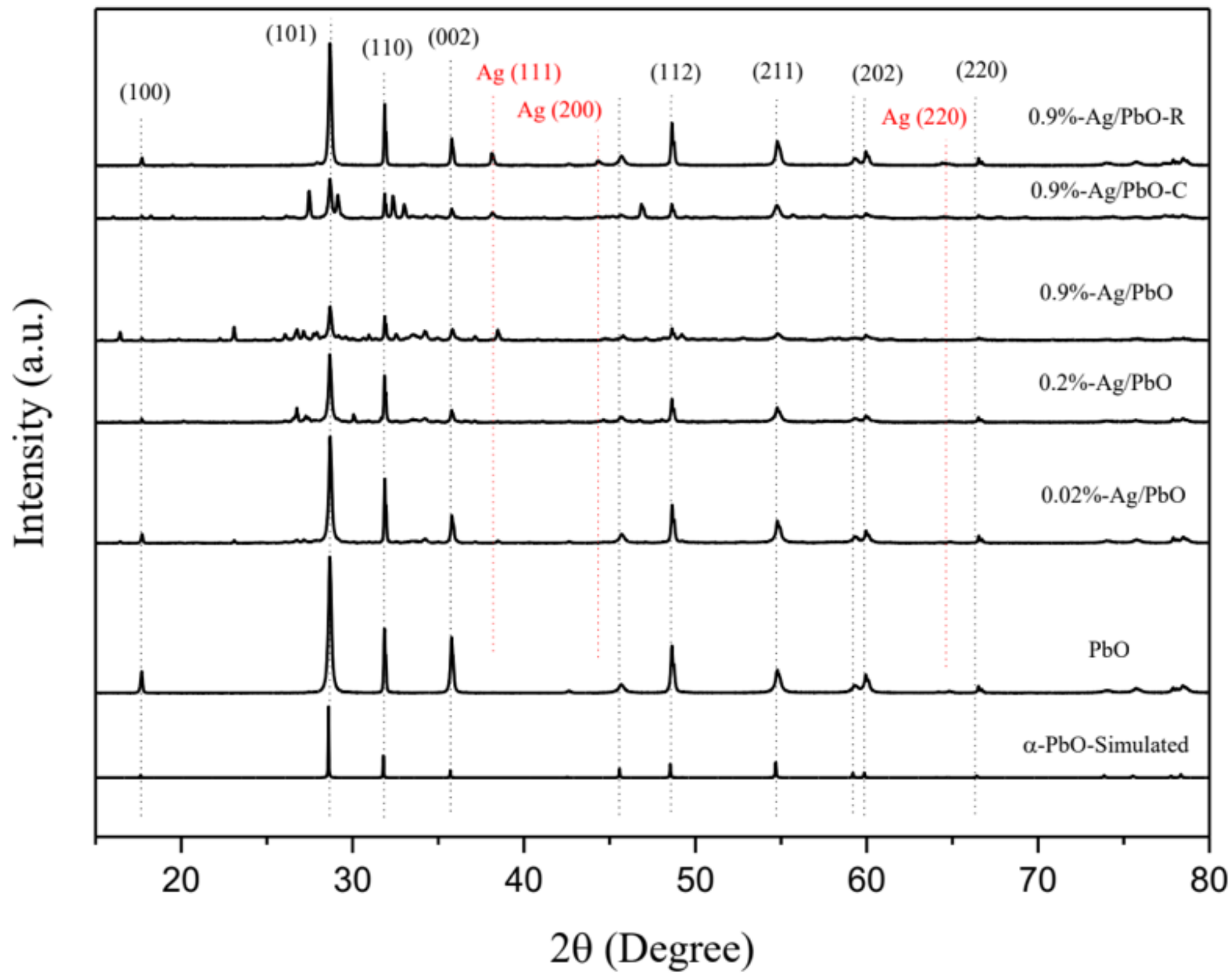

Figure S1. PXRD patterns of the tested materials. Note that 0.9%-Ag/PbO-C is the conditioned catalyst.

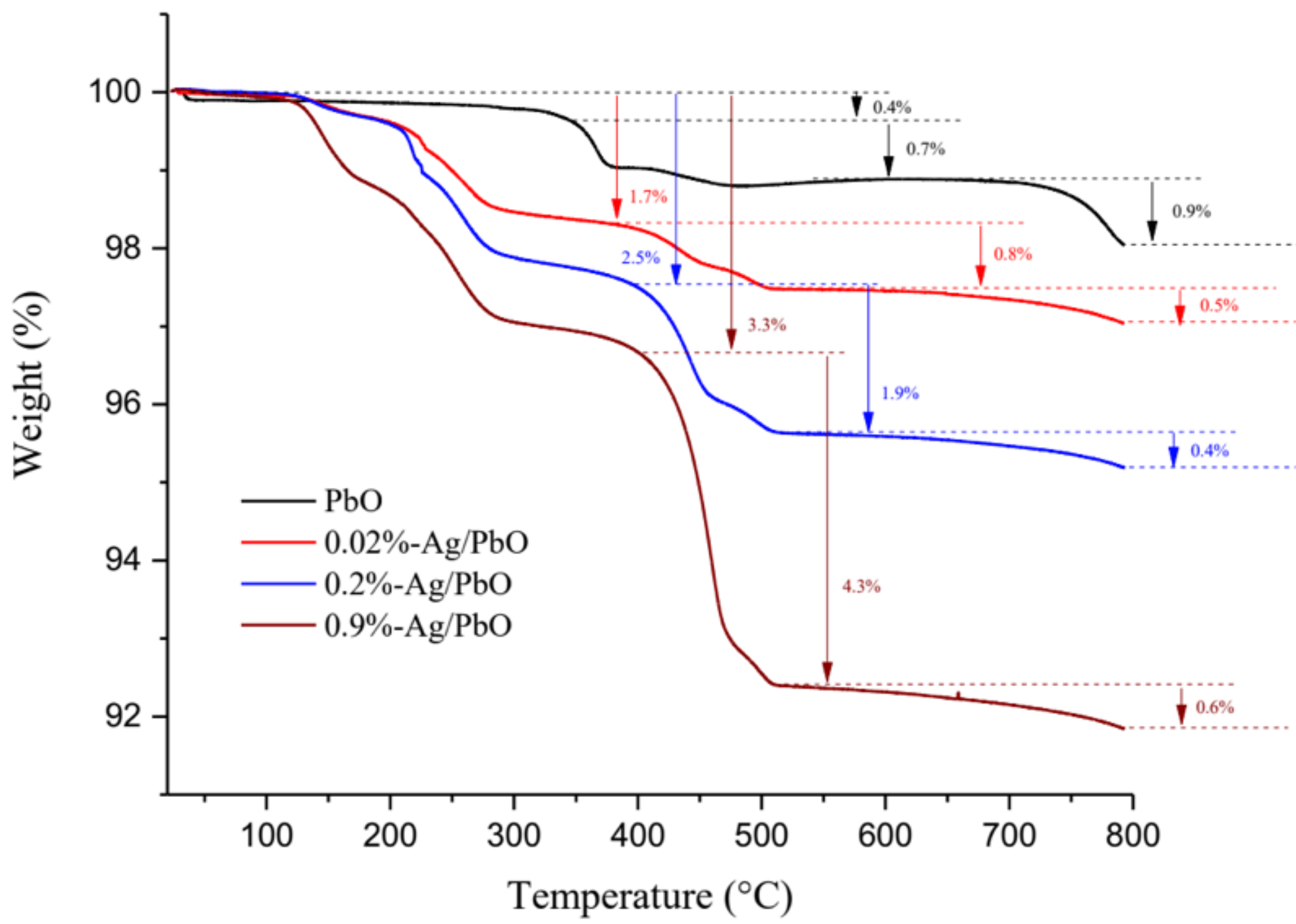

Figure S2. TGA profiles of the tested materials.

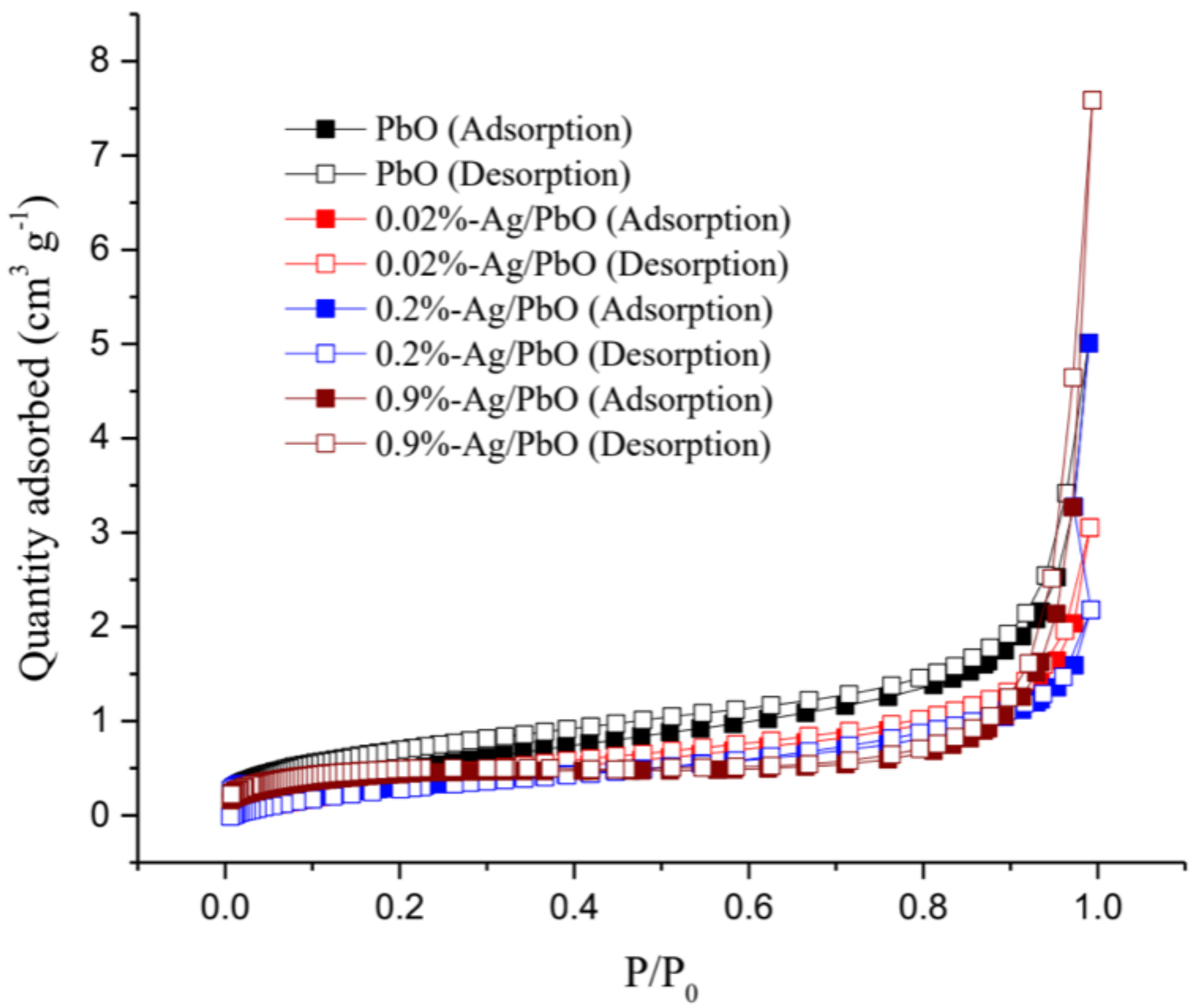

Figure S3. N_2 adsorption-desorption isotherms of the tested materials.

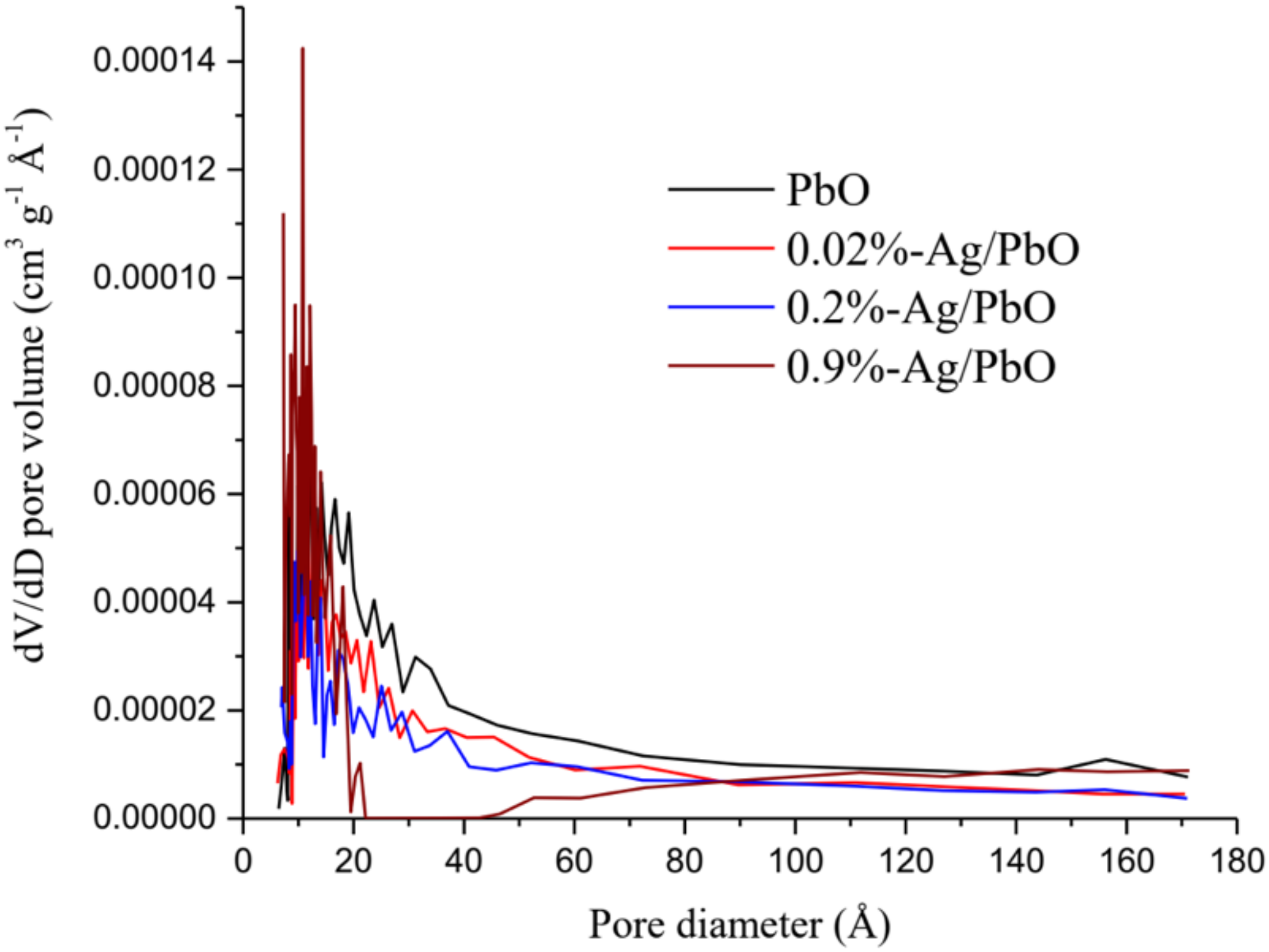

Figure S4. Pore size distribution of the tested materials.

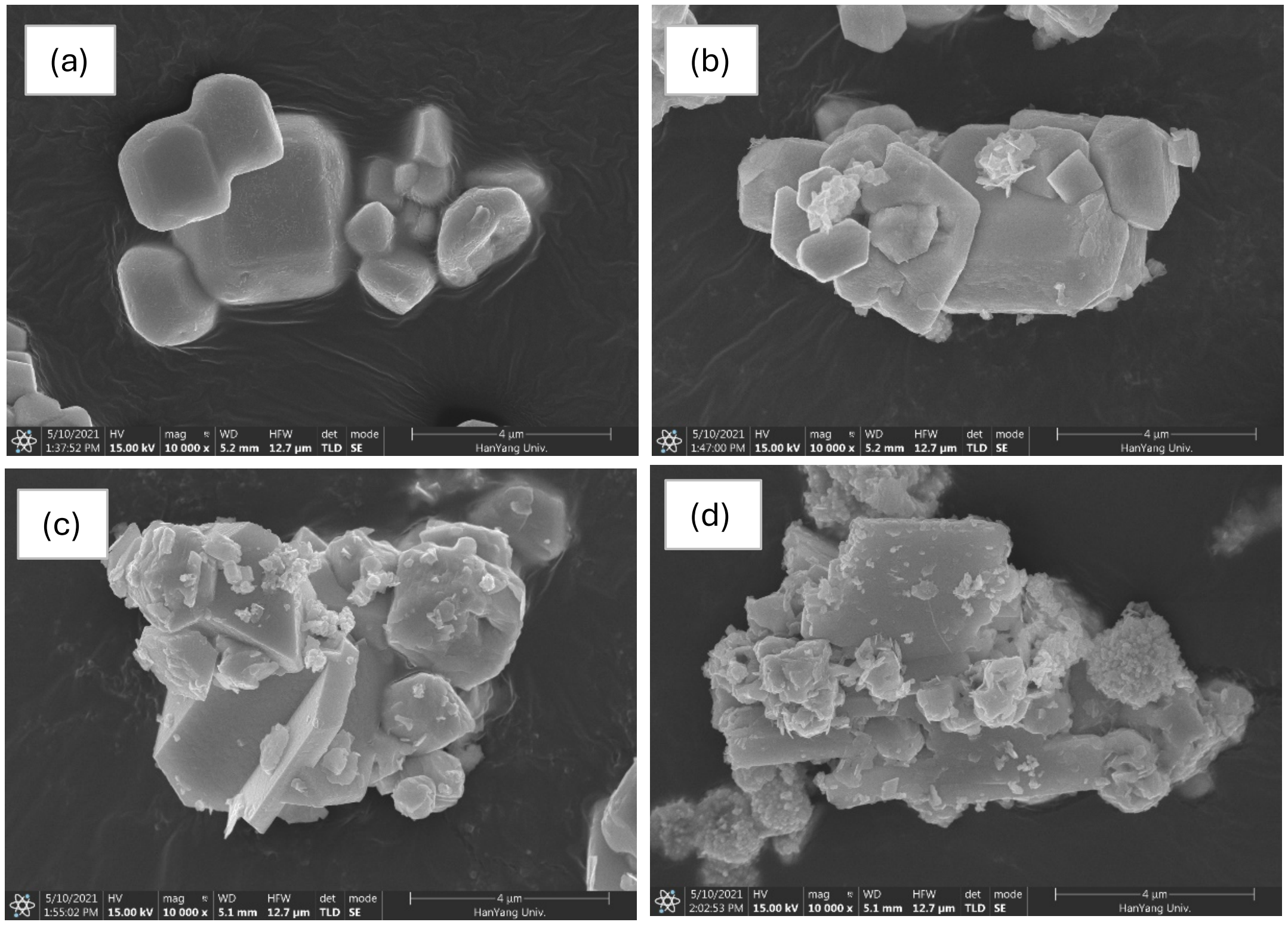

Figure S5. SEM images of the tested materials. Panel (a) PbO. Panel (b) 0.02%-Ag/PbO. Panel (c) 0.2%-Ag/PbO. Panel (d) 0.9%-Ag/PbO.

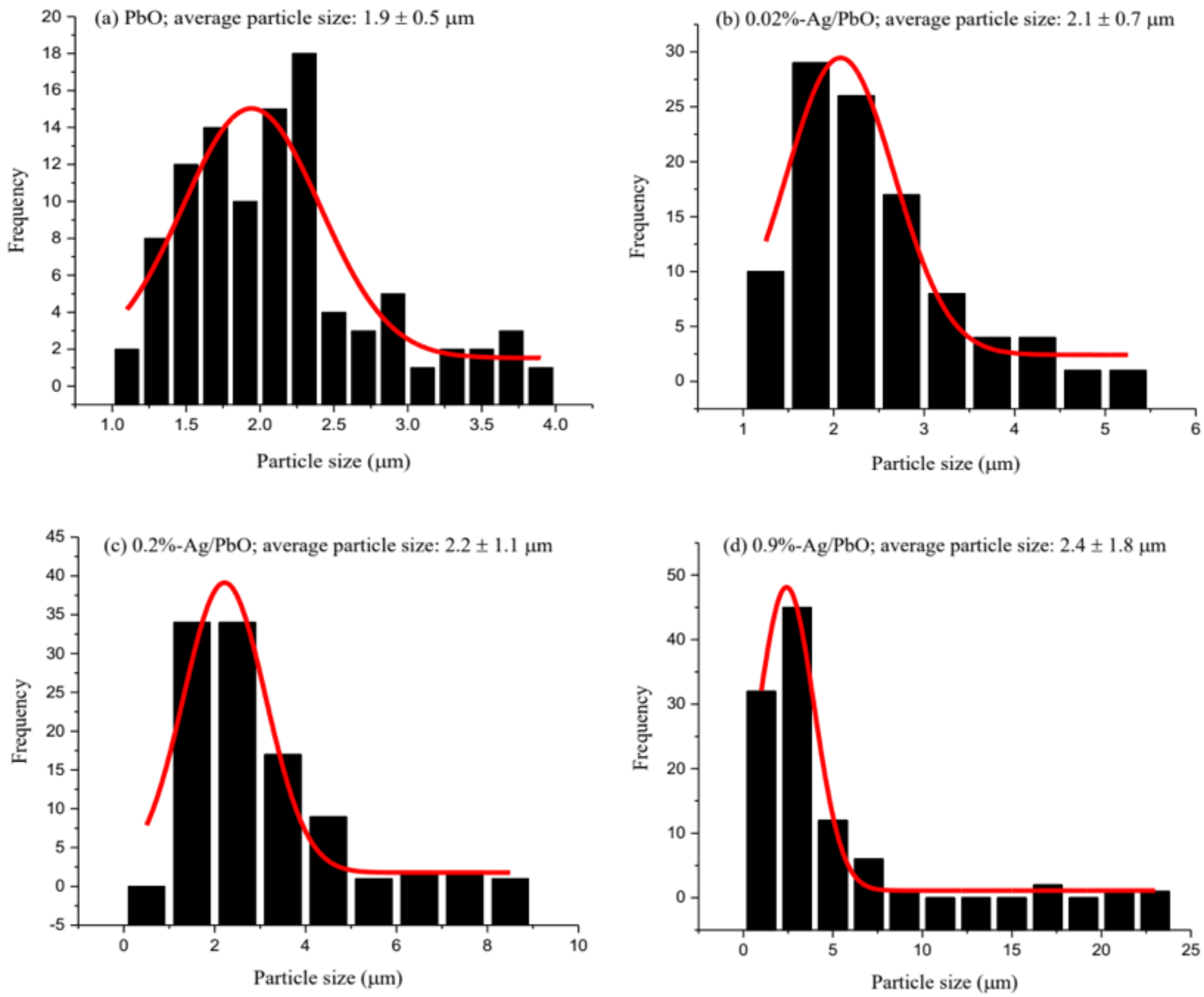

Figure S6. Particle size distribution of the tested materials. Panel (a) PbO. Panel (b) 0.02%-Ag/PbO. Panel (c) 0.2%-Ag/PbO. Panel (d) 0.9%-Ag/PbO.

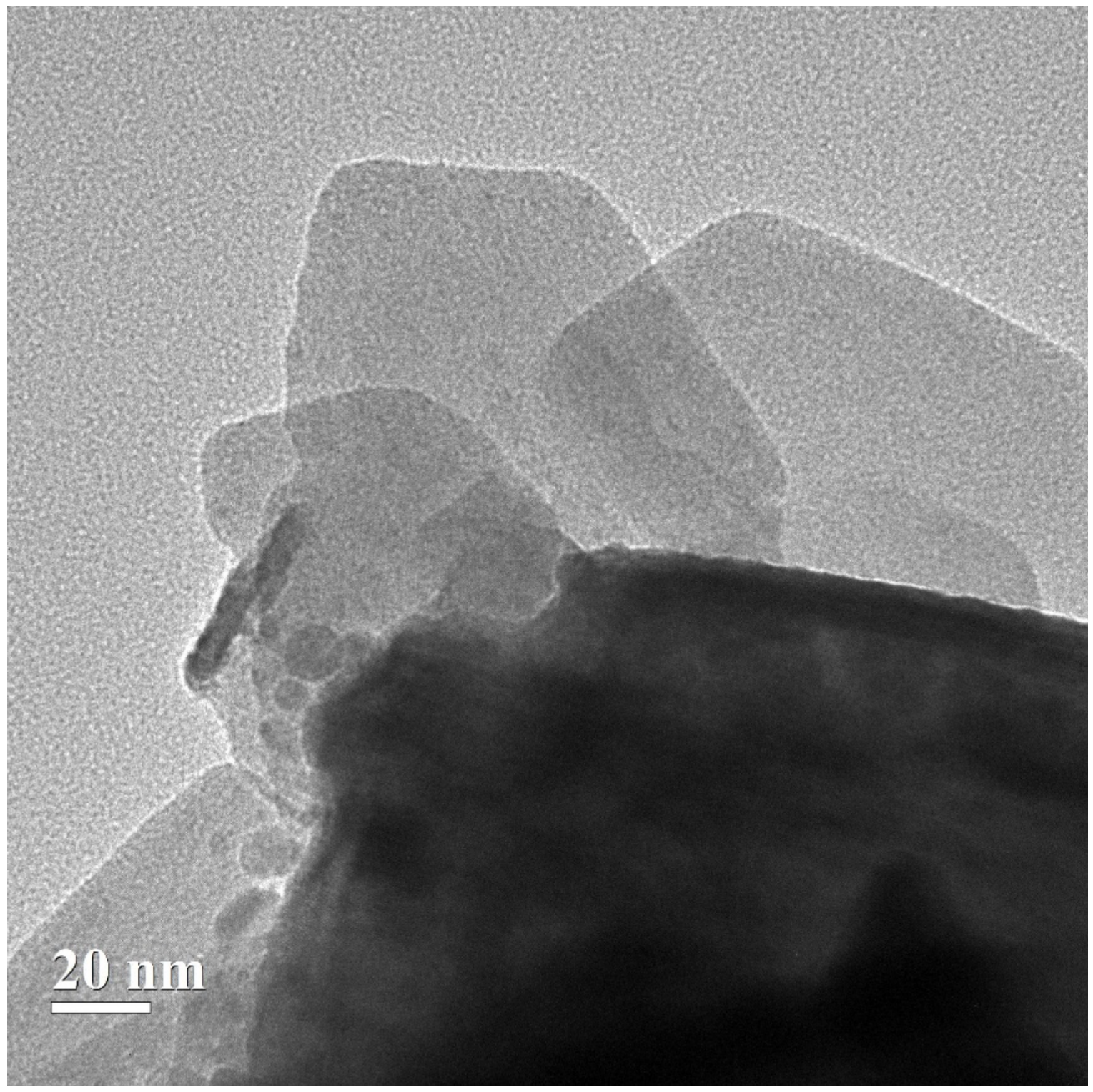

Figure S7. TEM image of PbO.

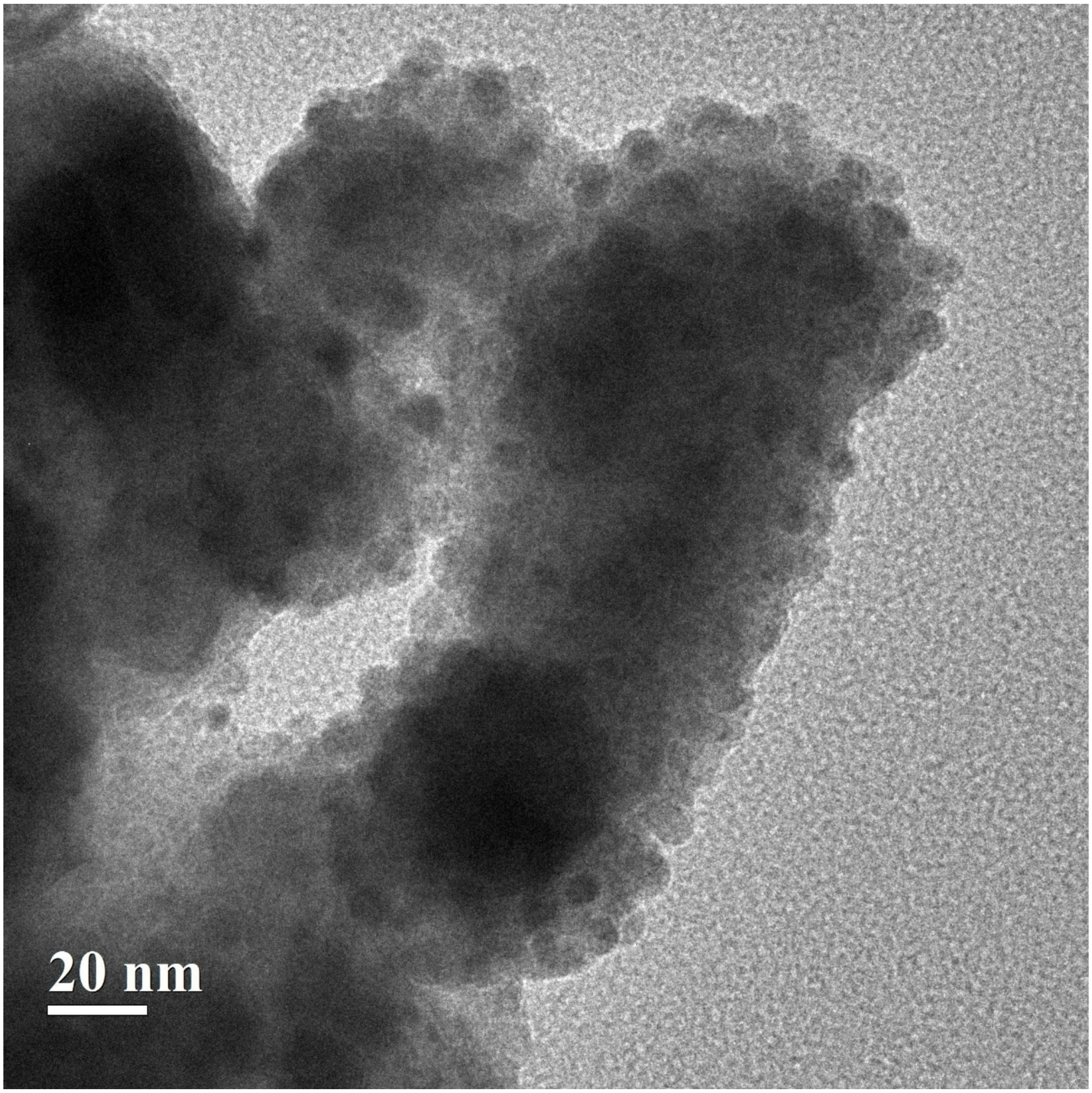

Figure S8. TEM image of 0.9%-Ag/PbO-C.

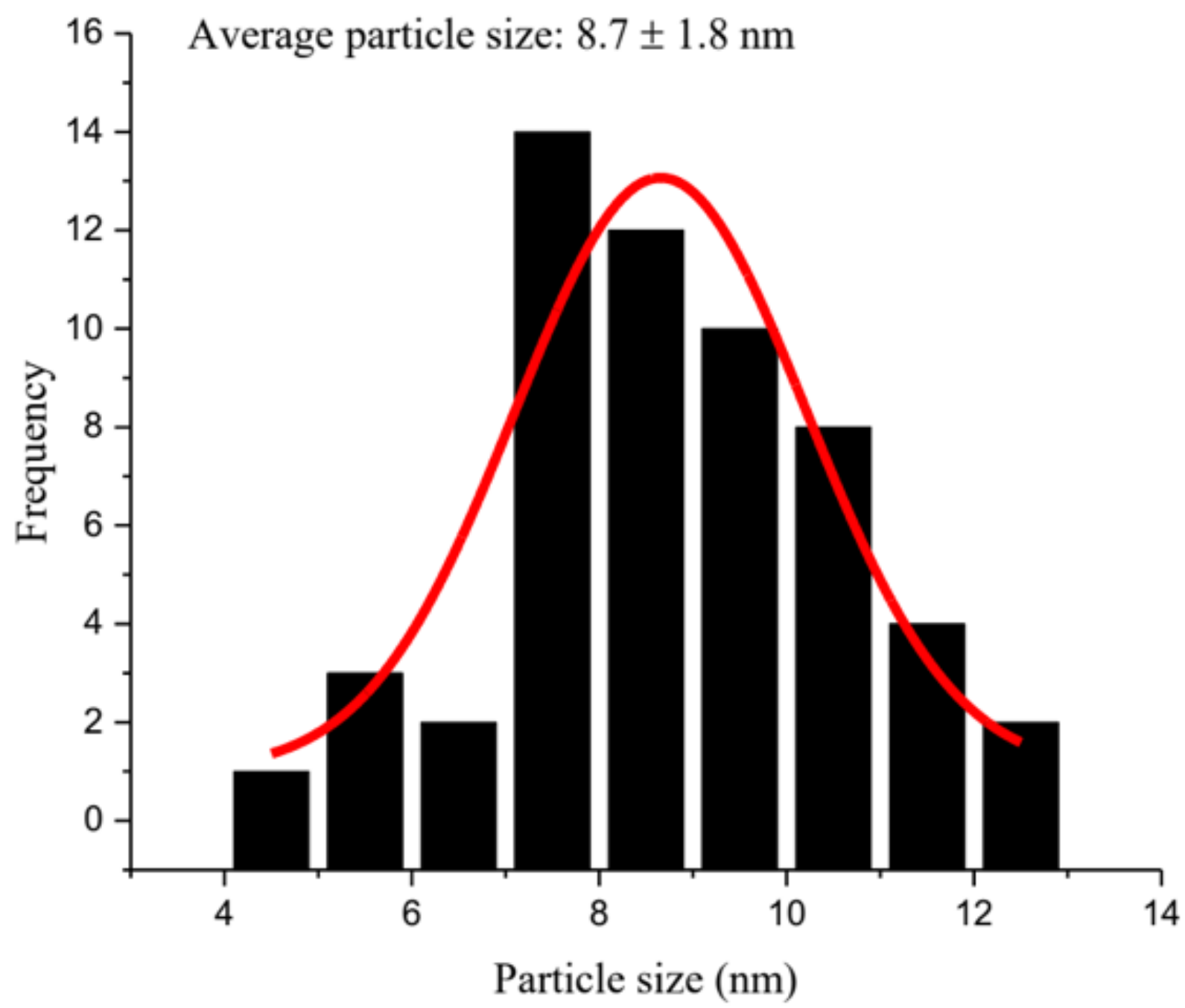

Figure S9. AgNP size distribution of 0.9%-Ag/PbO-C.

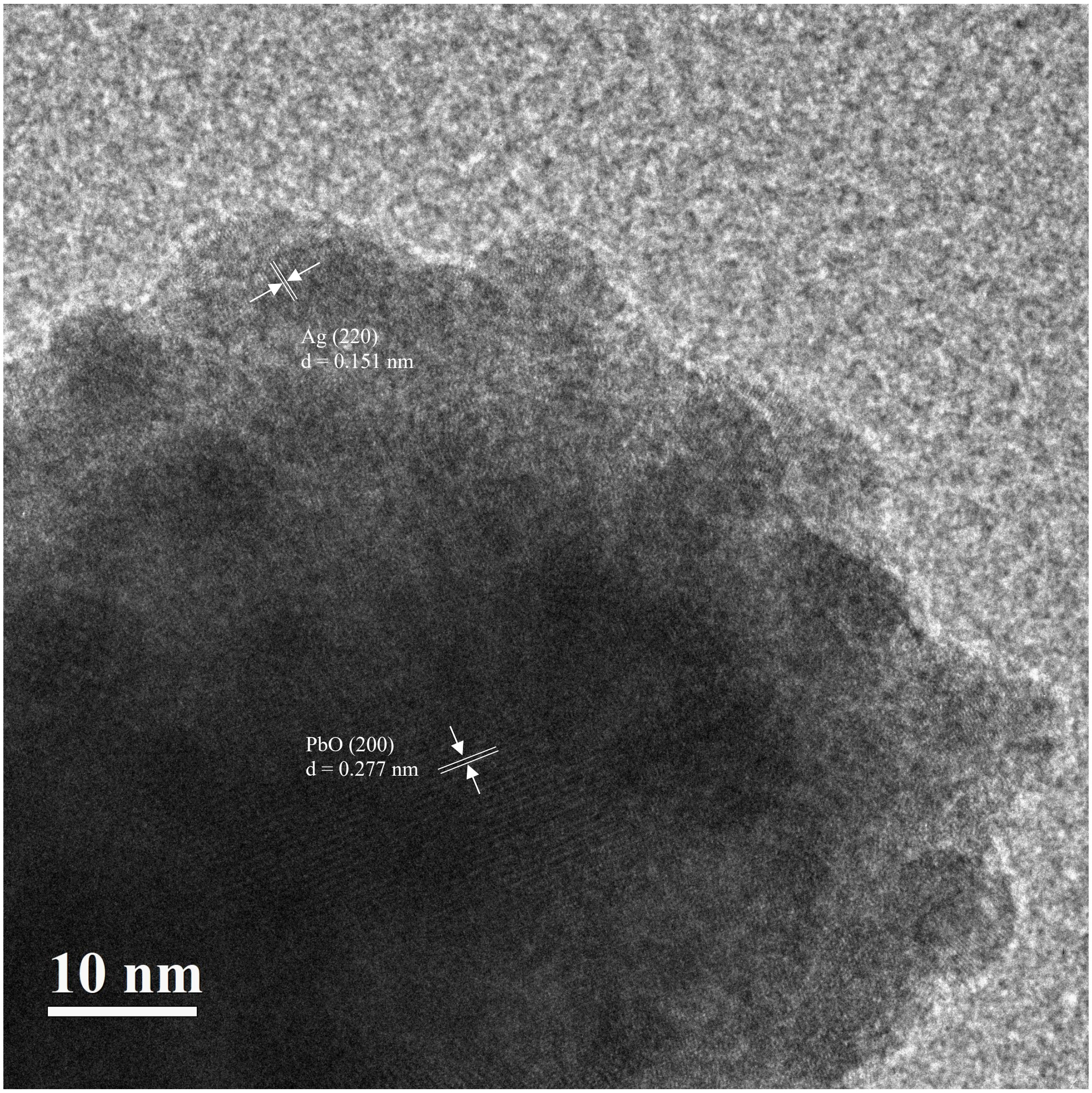

Figure S10. Magnified TEM image of 0.9%-Ag/PbO-C.

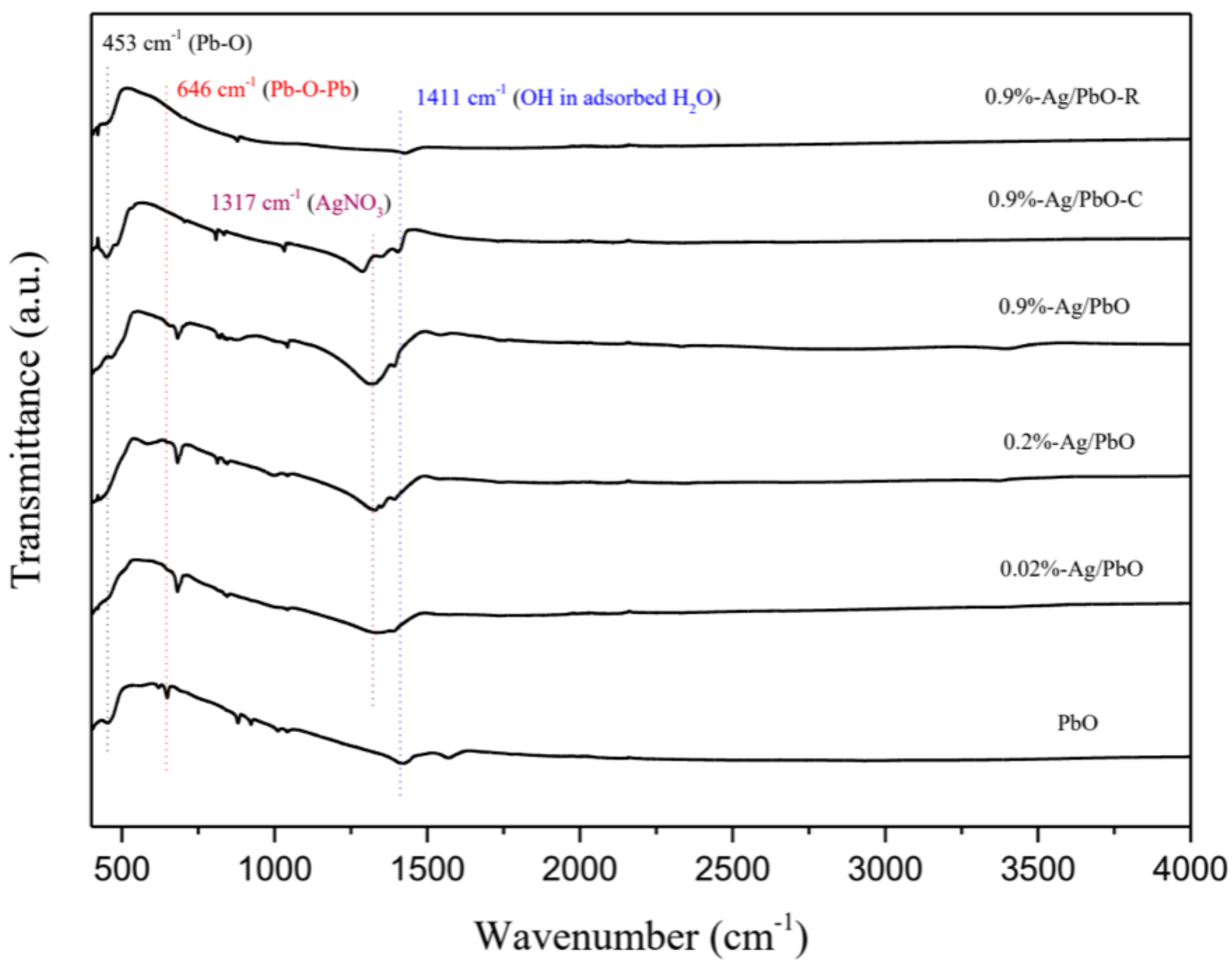

Figure S11. FTIR spectra of the tested materials.

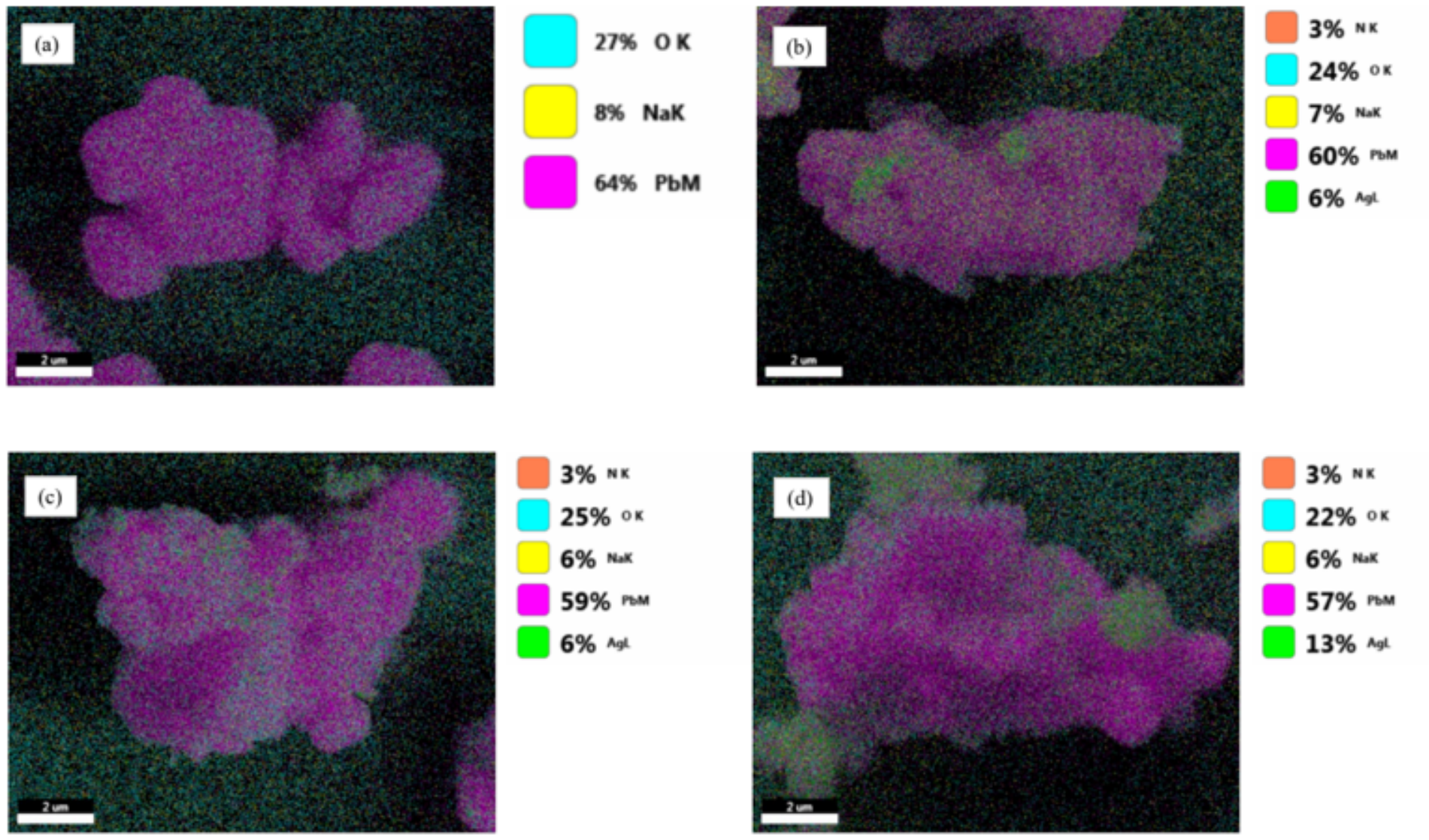

Figure S12. EDX elemental mapping of the tested materials. Panel (a) PbO. Panel (b) 0.02%-Ag/PbO. Panel (c) 0.2%-Ag/PbO. Panel (d) 0.9%-Ag/PbO.

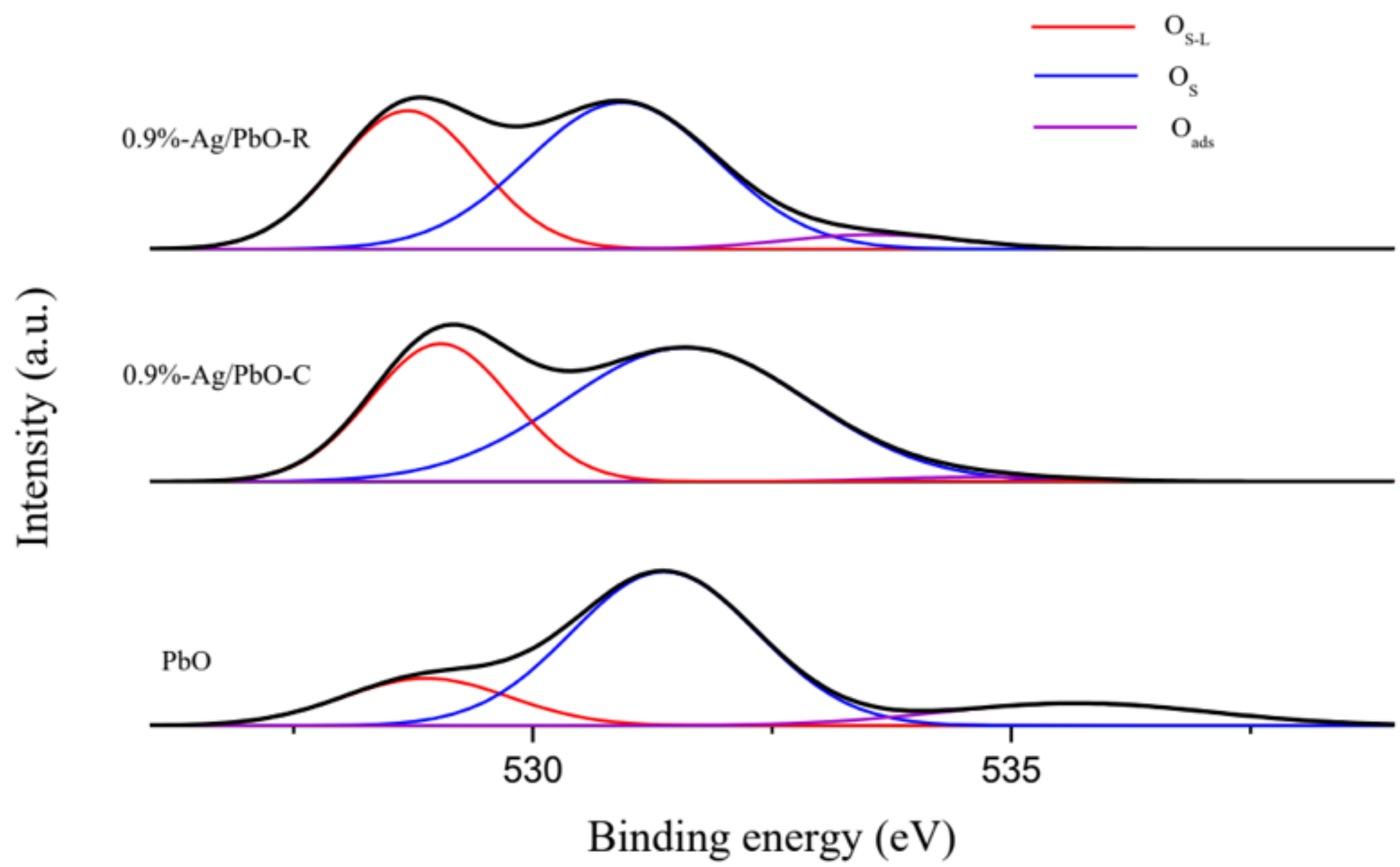

Figure S13. Deconvoluted core-level O 1s XPS spectra of the tested materials.

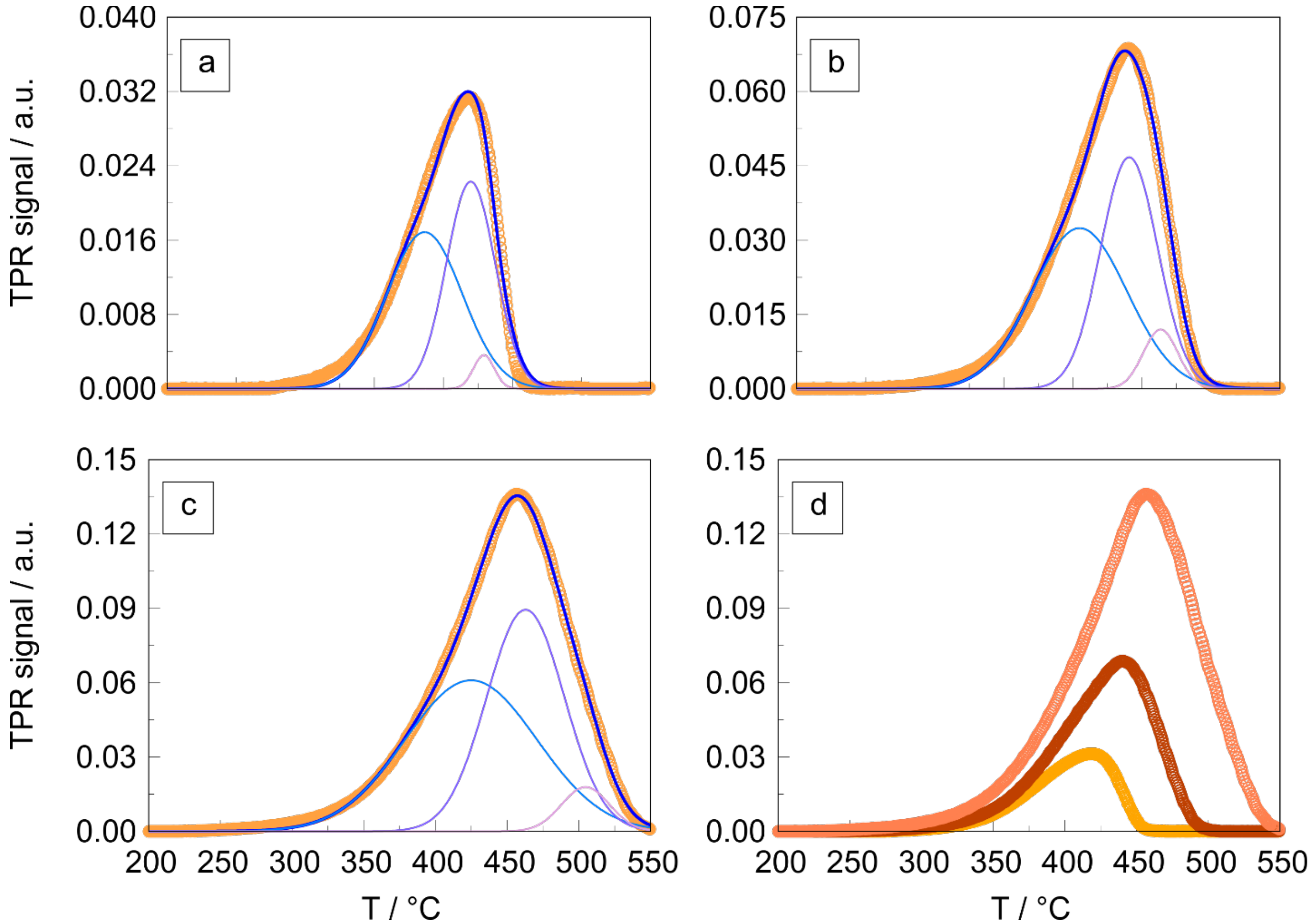

Figure S14. H_2-TPR profile for 0.9%-Ag/PbO-R at different heating rates. (a) $\beta = 1.6$ K min^{-1}, (b) $\beta = 4$ K min^{-1}, (c) $\beta = 10$ K min$^{-1,}$ and (d) all experimental data (o) $\beta = 1.6$ K min^{-1}, (o) $\beta = 4$ K min^{-1} and (o) $\beta = 10$ K min^{-1}. Gaussian model for (—) peak 1, (—) peak 2, (—) peak 3, and (—) total calculated signal in part (a) to (c).

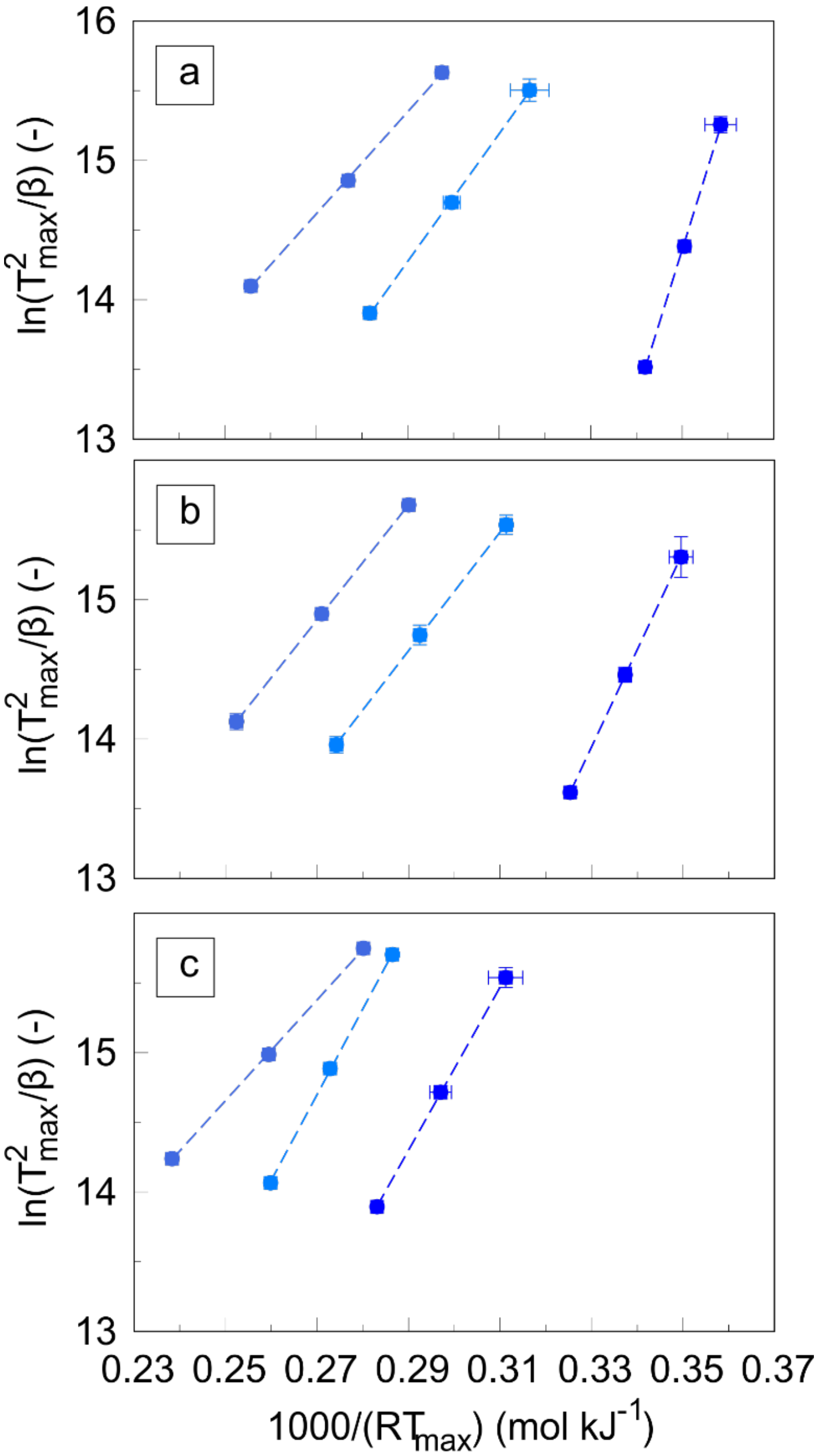

Figure S15. Modified data couples (T_{max}, β) for (●) peak 1, (●) peak 2, and (●) peak 3. Dashed lines represent the linear regression per peak. (a) PbO, (b) 0.9%-Ag/PbO, and (c) 0.9%-Ag/PbO-R.

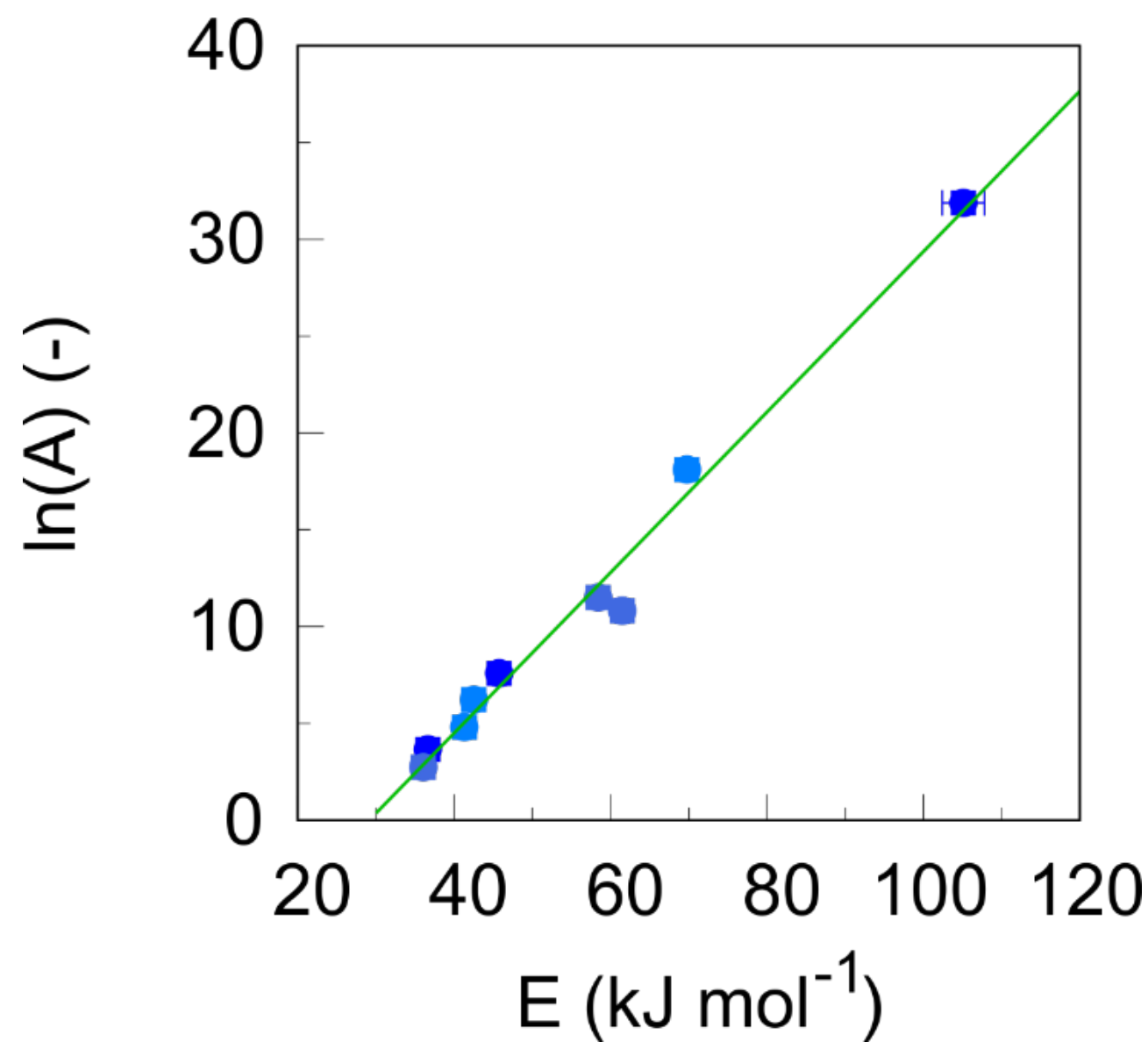

Figure S16. Compensation plot for the catalysts used. (●) PbO, (●) 0.9%-Ag/PbO, and (●) 0.9%-Ag/PbO-R. Full line (—) represents a simple linear regression.

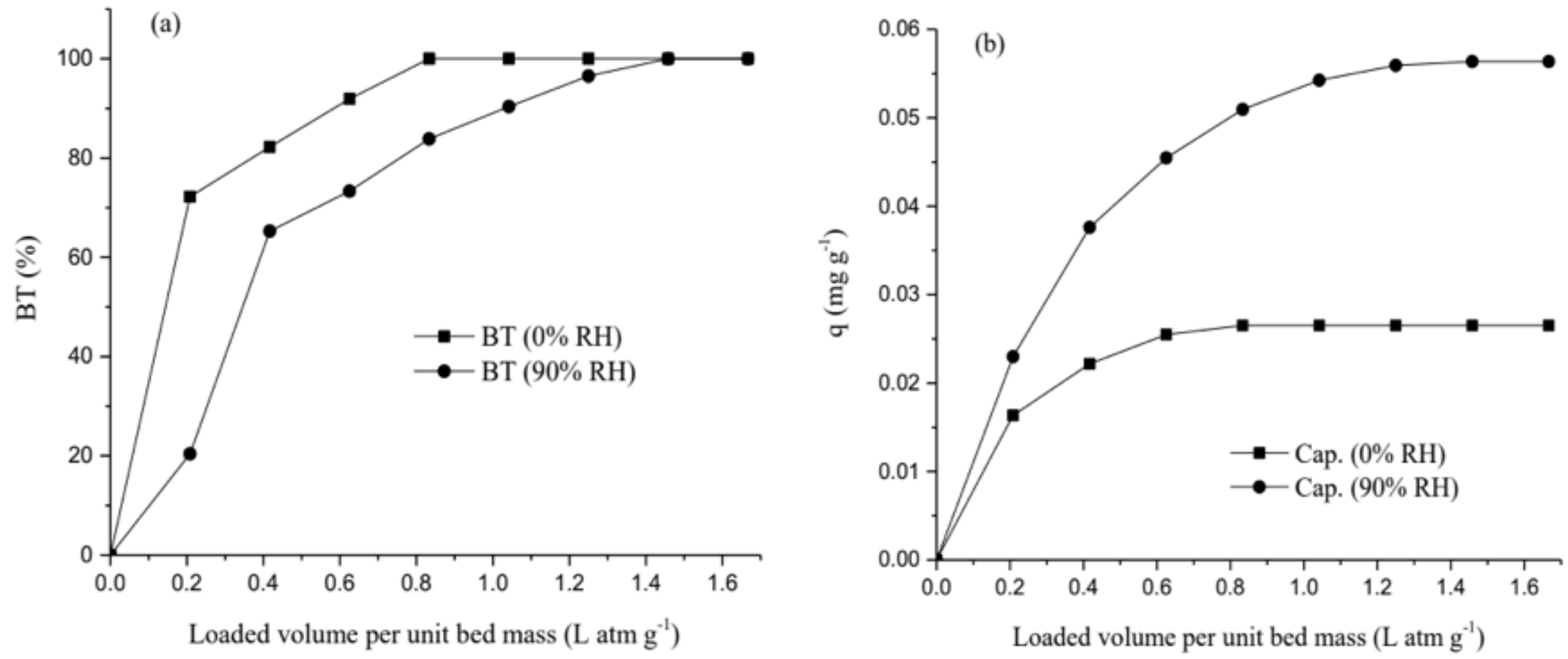

Figure S17. FA adsorption performance of PbO as a function of RH. Panel (a) BT. Panel (b) q. Note that 'Cap.' stands for 'Capacity.'

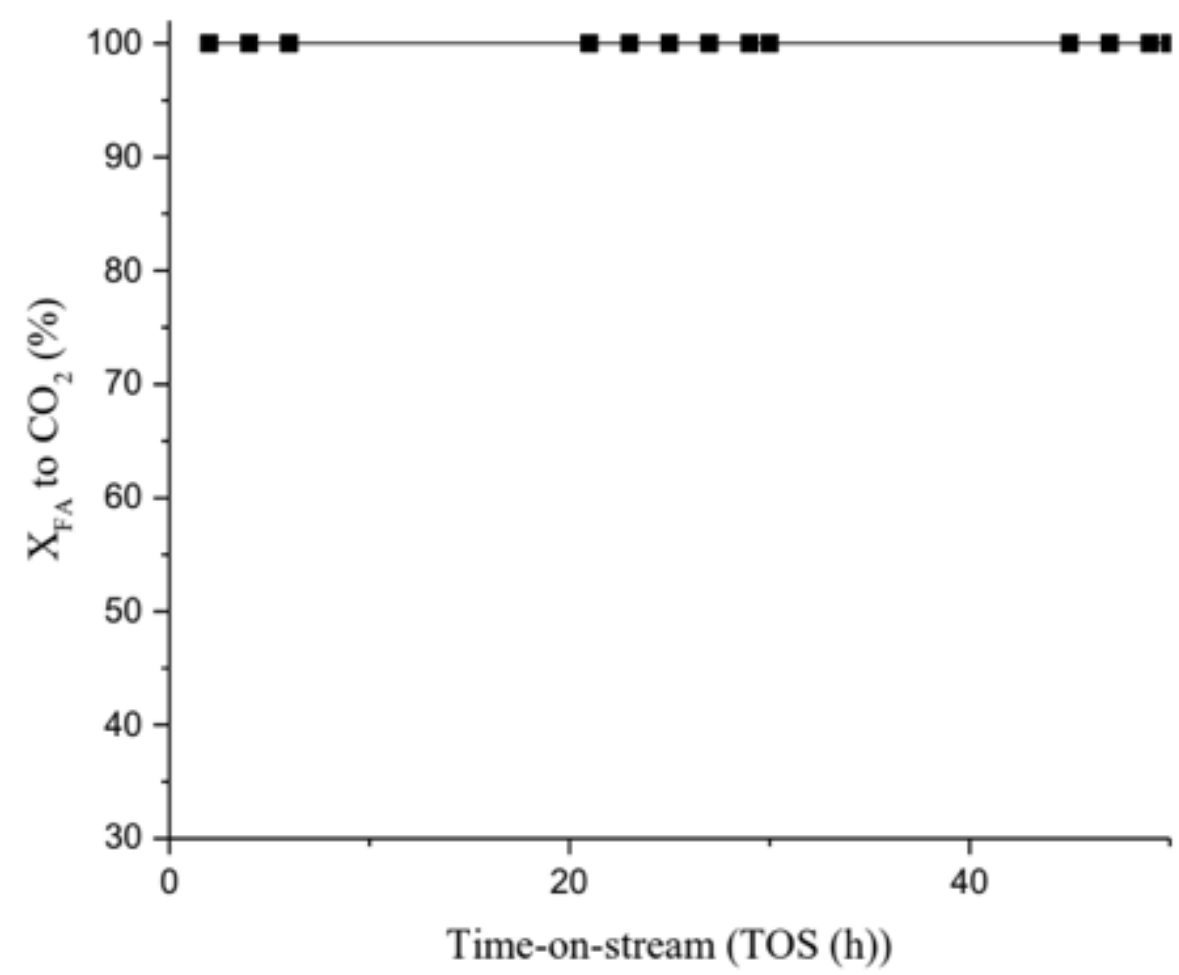

Figure S18. Time-on-stream (TOS) stability test for 0.9%-Ag/PbO (FA concentration: 100 ppm, catalyst mass = 300 mg, flow rate = 25 mL min^{-1}, and RH = 90%).

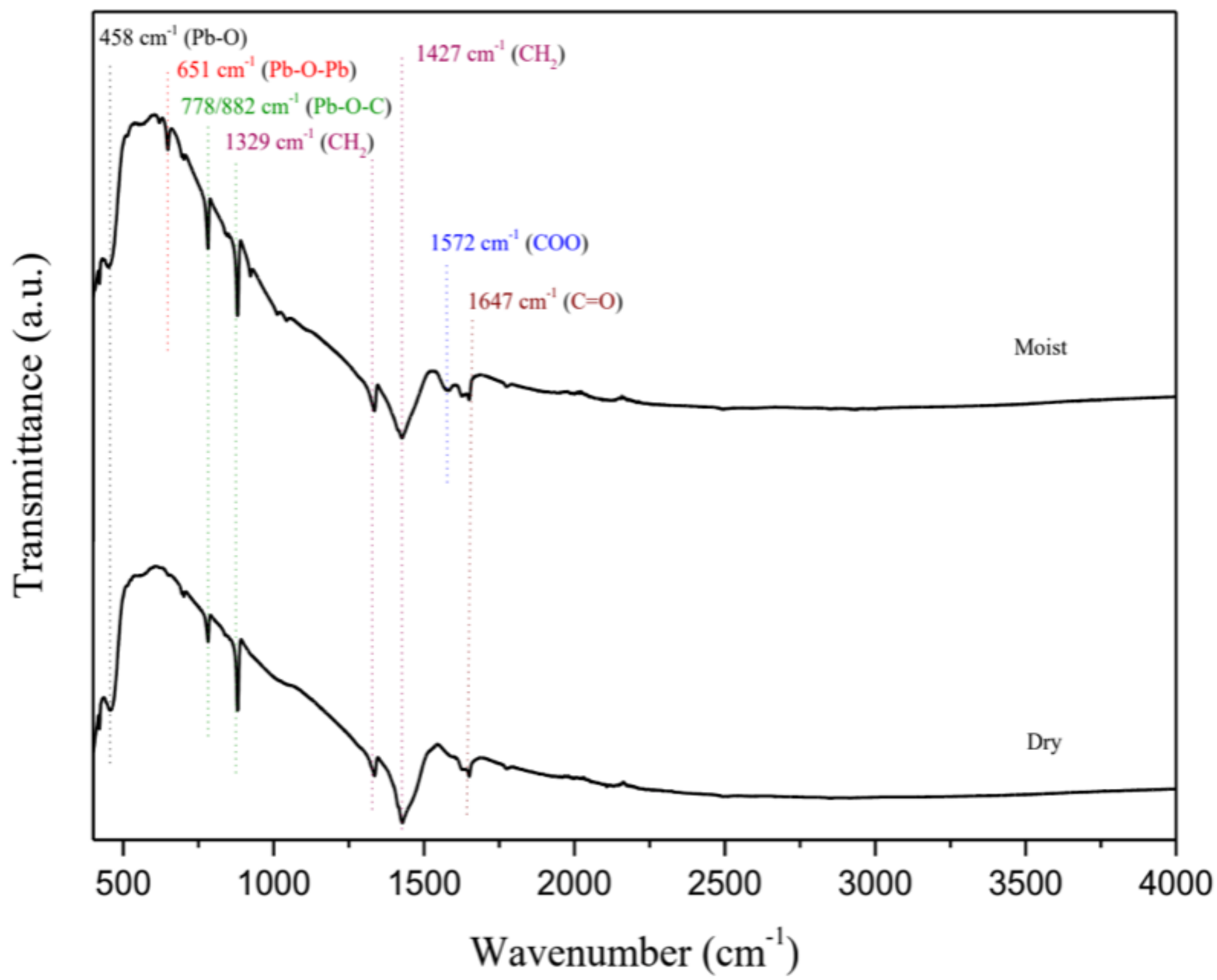

Figure S19. FTIR spectra of PbO after FA adsorption under dry and moist conditions.

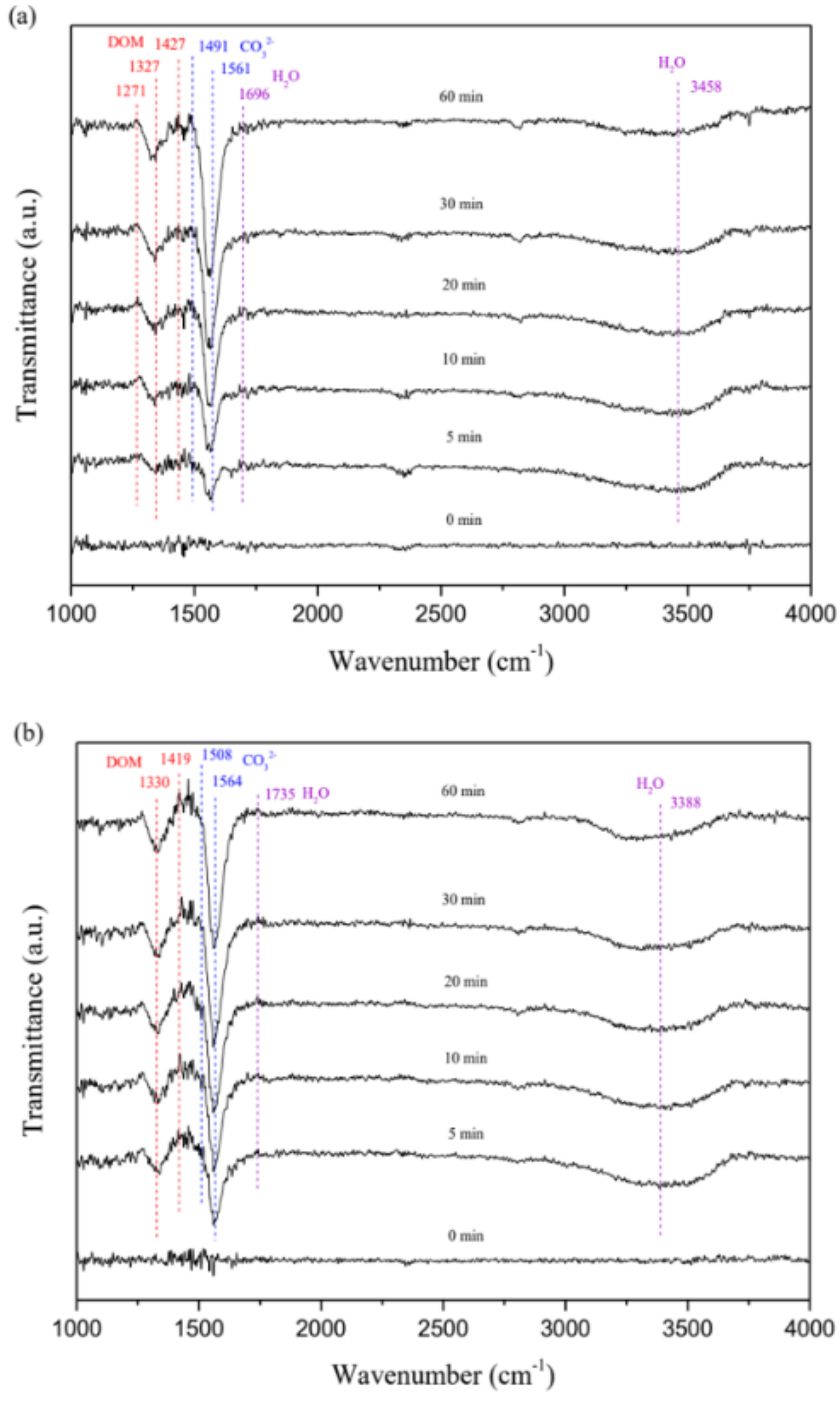

Figure S20. *In-situ* DRIFTS spectrum (100°C) for 0.9%-Ag/PbO-R. Panel (a) FA + air. Panel (b) FA + N_2.

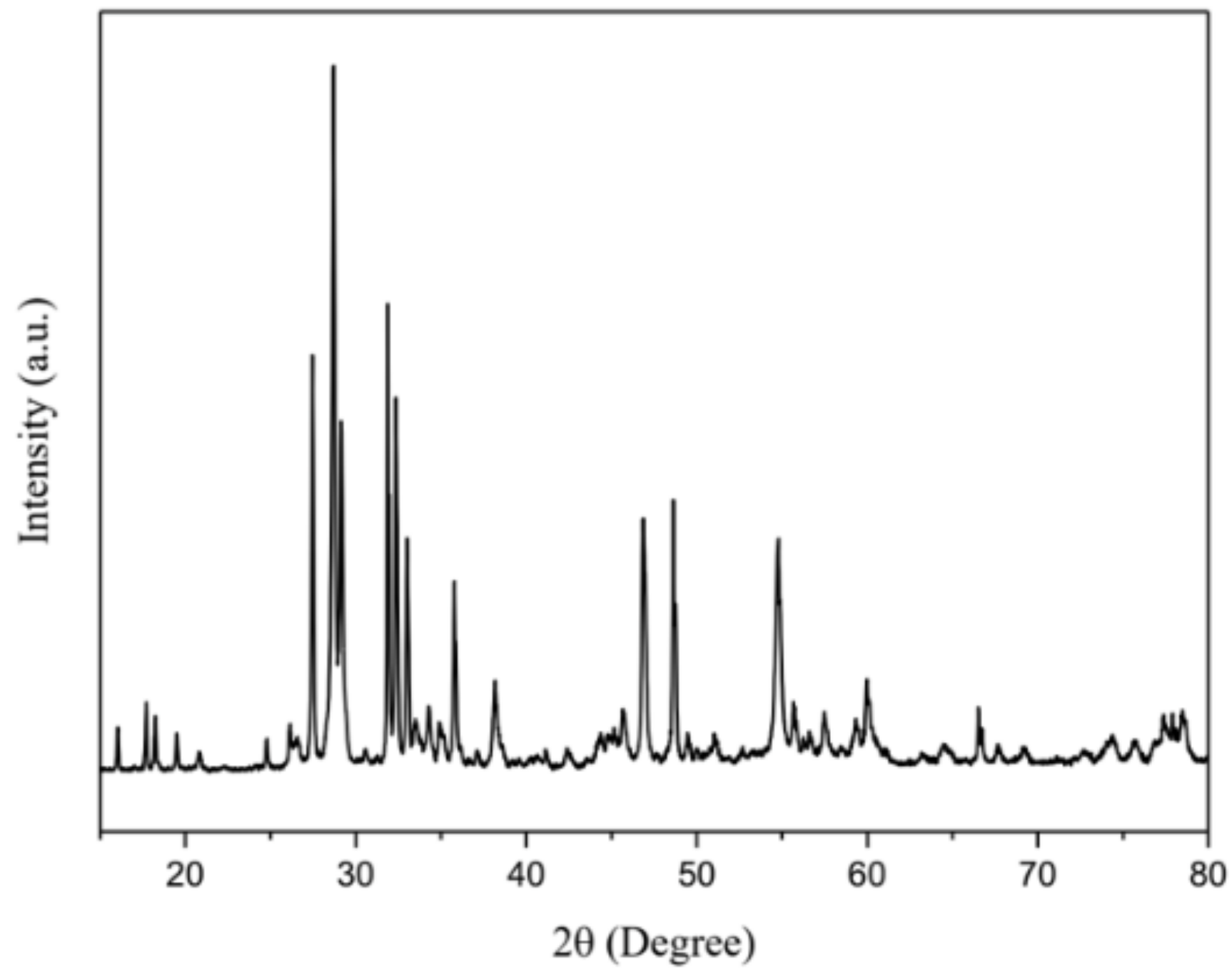

Figure S21. 0.9%-Ag/PbO-C PXRD pattern after the FA oxidation reaction.

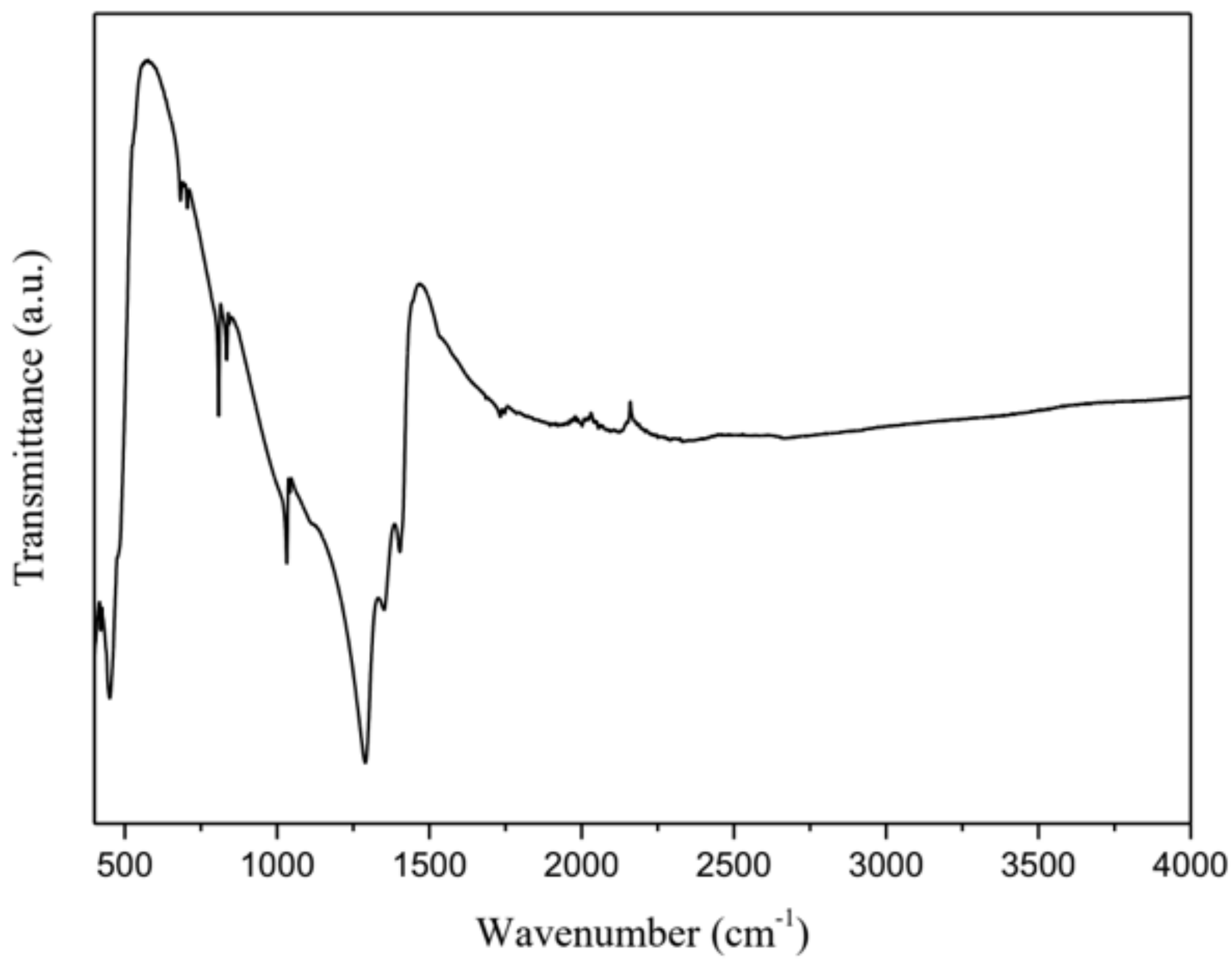

Figure S22. 0.9%-Ag/PbO-C FTIR spectrum after the FA oxidation reaction.

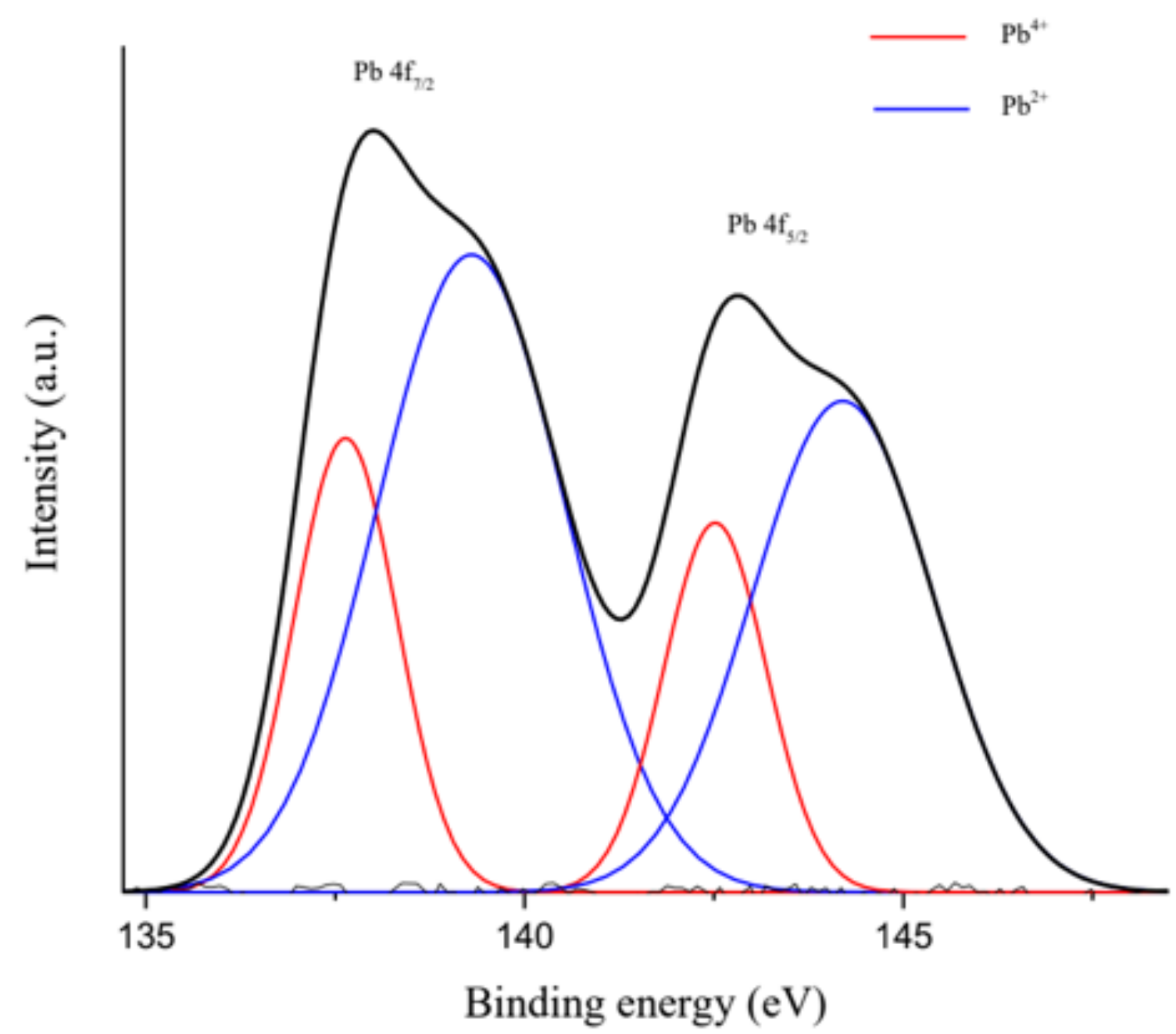

Figure S23. 0.9%-Ag/PbO-C deconvoluted Pb 4f XPS spectrum after the FA oxidation reaction.

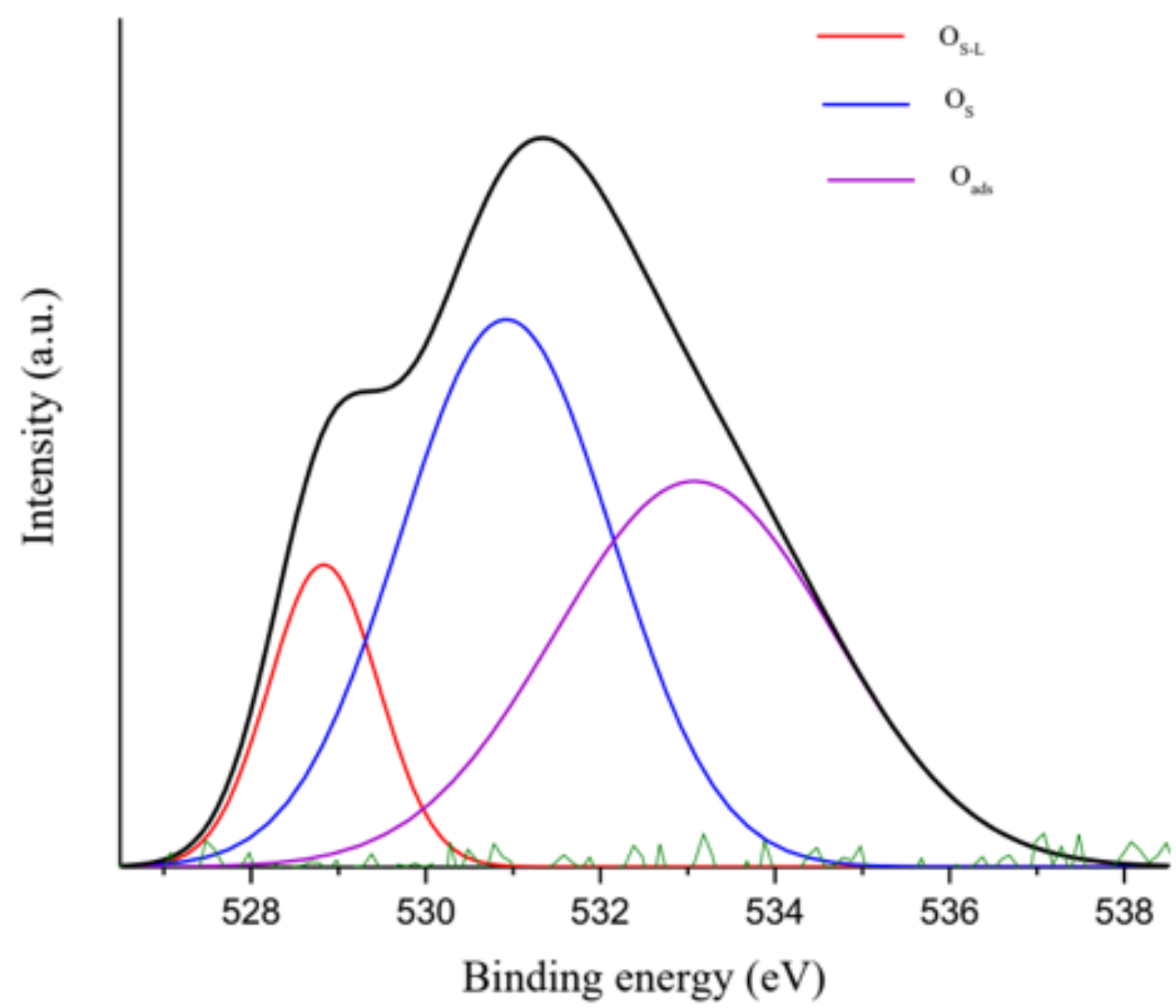

Figure S24. 0.9%-Ag/PbO-C deconvoluted O 1s XPS spectrum after the FA oxidation reaction.

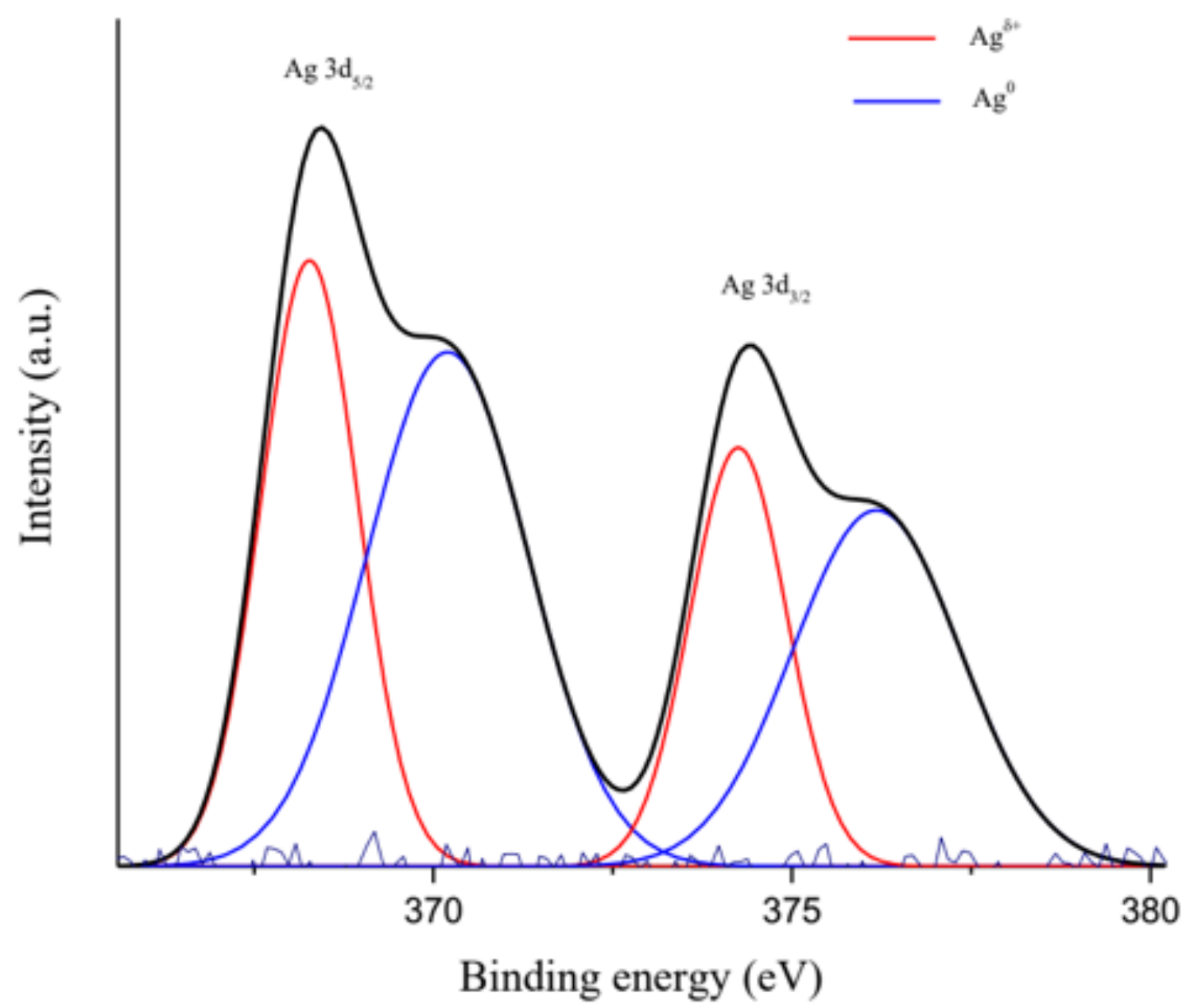

Figure S25. 0.9%-Ag/PbO-C deconvoluted Ag 3d XPS spectrum after the FA oxidation reaction.

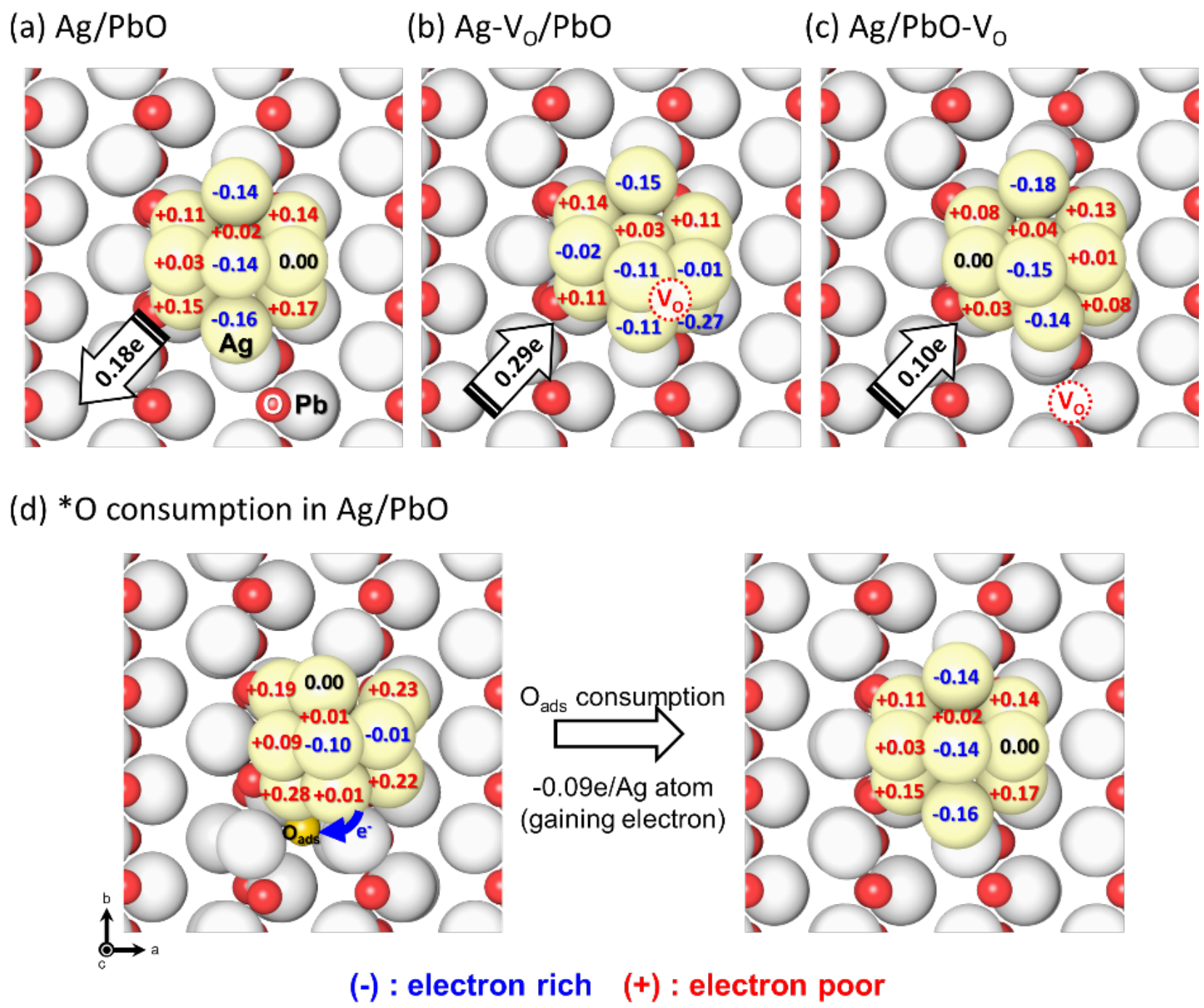

Figure S26. DFT simulation results. Panels (a)-(c) show the charge state distribution of Ag by the location of V_O based on Ag^0. Panel (d) illustrates the change with the consumption of O_{ads}.

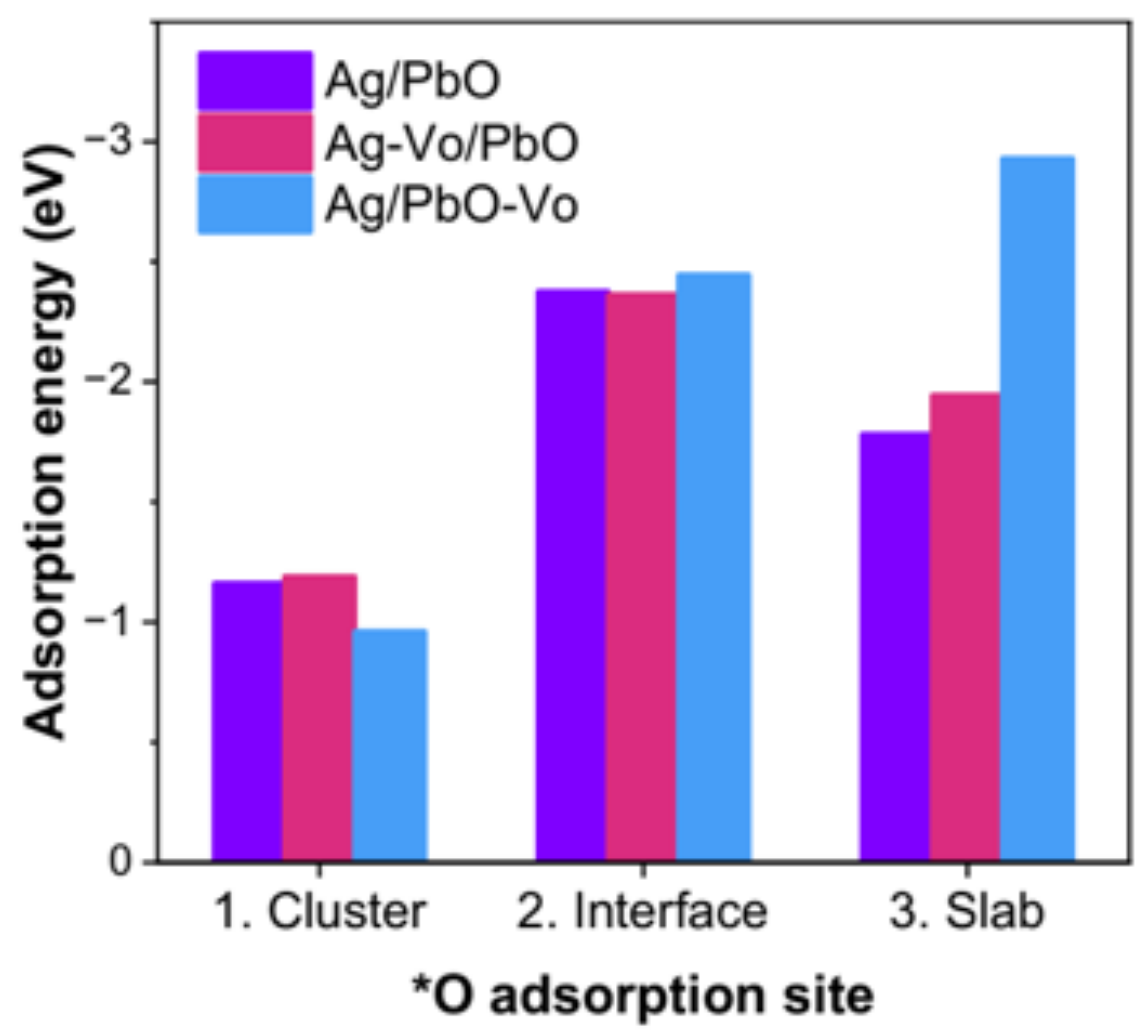

Figure S27. Adsorption properties of oxygen on each reaction site. The dotted line indicates the oxygen adsorption energy on the PbO slab.

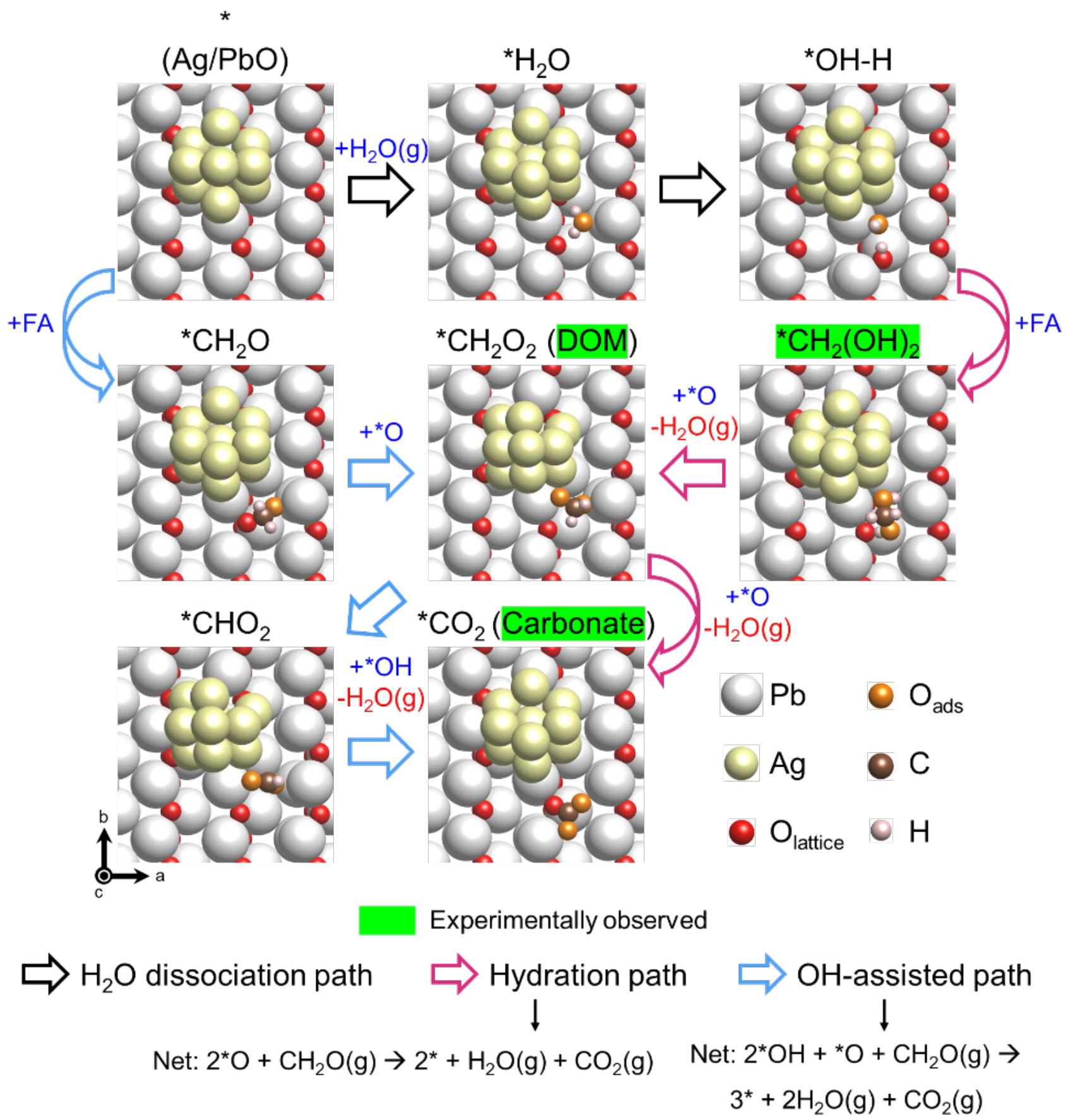

Figure S28. Reaction pathway and geometries (top view) of FA oxidation on Ag/PbO.

S10. References

[1] K. Momma, F. Izumi, *VESTA 3* for three-dimensional visualization of crystal, volumetric and morphology data, Journal of Applied Crystallography 44(6) (2011) 1272-1276.

[2] C.A. Schneider, W.S. Rasband, K.W. Eliceiri, NIH Image to ImageJ: 25 years of image analysis, Nature Methods 9(7) (2012) 671-675.

[3] S. Rudd, R. Mahjoub, E. Switalska, C. Bassell, N. Schmerl, A.-A. Cavallaro, N. Stanford, D.R. Evans, Surface Doping in Poly(3,4-ethylenedioxythiophene)-Based Nanoscale Films: Insights for Polymer Electronics, ACS Applied Nano Materials 5(9) (2022) 12143-12153.

[4] P. Malet, A. Caballero, The selection of experimental conditions in temperature-programmed reduction experiments, Journal of the Chemical Society, Faraday Transactions 1: Physical Chemistry in Condensed Phases 84(7) (1988) 2369-2375.

[5] P.A. Redhead, Thermal desorption of gases, Vacuum 12(4) (1962) 203-211.

[6] P.M. Heynderickx, J.W. Thybaut, H. Poelman, D. Poelman, G.B. Marin, The total oxidation of propane over supported Cu and Ce oxides: A comparison of single and binary metal oxides, Journal of Catalysis 272(1) (2010) 109-120.

[7] K. Vikrant, K.-H. Kim, F. Dong, P.M. Heynderickx, D.W. Boukhvalov, Low-temperature oxidative removal of gaseous formaldehyde by an eggshell waste supported silver-manganese dioxide bimetallic catalyst with ultralow noble metal content, Journal of Hazardous Materials 434 (2022) 128857.

[8] G.F. Froment, The kinetics of complex catalytic reactions, Chemical Engineering Science 42(5) (1987) 1073-1087.

[9] R. de Levie, Estimating Parameter Precision in Nonlinear Least Squares with Excel's Solver, Journal of Chemical Education 76(11) (1999) 1594.

[10] A.K. Coker, Appendix C: Physical Properties of Liquids and Gases, in: A.K. Coker (Ed.), Ludwig's Applied Process Design for Chemical and Petrochemical Plants (Fourth Edition), Gulf Professional Publishing, Burlington, 2007, pp. 827-862.

[11] N.I. Anisimova, G.A. Bordovsky, V.A. Bordovsky, V.I. Seldayev, Electrical and thermal properties of Bi_2O_3/PbO and mixed oxides of Bi_2O_3-PbO system, Proceedings of the 2004 IEEE International Conference on Solid Dielectrics, 2004. ICSD 2004., 2004, pp. 141-142 Vol.1.

[12] G. Bergeret G., P. Gallezot P. Particle Size and Dispersion Measurements. Handbook of Heterogeneous Catalysis, 2, Wiley-VCH, pp.738-765, 2008.
